# X-ray multiscale 3D neuroimaging to quantify cellular aging and neurodegeneration postmortem in a model of Alzheimer’s disease

**DOI:** 10.1007/s00259-022-05896-5

**Published:** 2022-07-19

**Authors:** Giacomo E. Barbone, Alberto Bravin, Alberto Mittone, Alexandra Pacureanu, Giada Mascio, Paola Di Pietro, Markus J. Kraiger, Marina Eckermann, Mariele Romano, Martin Hrabě de Angelis, Peter Cloetens, Valeria Bruno, Giuseppe Battaglia, Paola Coan

**Affiliations:** 1 Department of Medical Physics, Faculty of Physics, Ludwig-Maximilians-Universität München, Am Coulombwall 1, 85748 Garching, Germany; 2 Department of Clinical Radiology, Faculty of Medicine, Ludwig-Maximilians-Universität München, Munich, Germany; 3 ESRF, The European Synchrotron, Grenoble, France; 4 Department of Physics, University Milano Bicocca, Milan, Italy; 5 CELLS-ALBA Synchrotron, Cerdanyola del Vallès, Spain; 6 Department of Molecular Pathology, I.R.C.C.S. Neuromed, Pozzilli, Italy; 7 Present Address: Department of Medicine, Surgery and Dentistry, “Scuola Medica Salernitana”, University of Salerno, Baronissi, Italy; 8 Institute of Experimental Genetics, German Mouse Clinic, Helmholtz Zentrum München, German Research Center for Environmental Health, Neuherberg, Germany; 9 Institut Für Röntgenphysik, Georg-August-Universität Göttingen, Göttingen, Germany; 10 Chair of Experimental Genetics, TUM School of Life Sciences, Technische Universität München, Freising, Germany; 11 German Center for Diabetes Research, Helmholtz Zentrum München, German Research Center for Environmental Health, Neuherberg, Germany; 12 Department of Physiology and Pharmacology, University Sapienza, Piazzale Aldo Moro 5, 00185 Rome, Italy

**Keywords:** Neurodegeneration, Alzheimer’s disease, Metabotropic glutamate receptors, Nano-imaging, Micro-CT, Neuro-radiology

## Abstract

**Purpose:**

Modern neuroimaging lacks the tools necessary for whole-brain, anatomically dense neuronal damage screening. An ideal approach would include unbiased histopathologic identification of aging and neurodegenerative disease.

**Methods:**

We report the postmortem application of multiscale X-ray phase-contrast computed tomography (X-PCI-CT) for the label-free and dissection-free organ-level to intracellular-level 3D visualization of distinct single neurons and glia. In deep neuronal populations in the brain of aged wild-type and of 3xTgAD mice (a triply-transgenic model of Alzheimer’s disease), we quantified intracellular hyperdensity, a manifestation of aging or neurodegeneration.

**Results:**

In 3xTgAD mice, the observed hyperdensity was identified as amyloid-β and hyper-phosphorylated tau protein deposits with calcium and iron involvement, by correlating the X-PCI-CT data to immunohistochemistry, X-ray fluorescence microscopy, high-field MRI, and TEM. As a proof-of-concept, X-PCI-CT was used to analyze hippocampal and cortical brain regions of 3xTgAD mice treated with LY379268, selective agonist of group II metabotropic glutamate receptors (mGlu2/3 receptors). Chronic pharmacologic activation of mGlu2/3 receptors significantly reduced the hyperdensity particle load in the ventral cortical regions of 3xTgAD mice, suggesting a neuroprotective effect with locoregional efficacy.

**Conclusions:**

This multiscale micro-to-nano 3D imaging method based on X-PCI-CT enabled identification and quantification of cellular and sub-cellular aging and neurodegeneration in deep neuronal and glial cell populations in a transgenic model of Alzheimer’s disease. This approach quantified the localized and intracellular neuroprotective effects of pharmacological activation of mGlu2/3 receptors.

**Supplementary Information:**

The online version contains supplementary material available at 10.1007/s00259-022-05896-5.

## Background

Alzheimer’s disease (AD), the leading cause of age-related dementia in humans, occurs due to the convergence of diverse and still ill-understood pathological processes [1, 2]. Toxic protein deposition, synapse loss, and microglial infiltration precede neuronal death, leading to progressive cognitive decline. As with many neurodegenerative diseases, AD occurs secondary to neuronal protein misfolding. Aggregates of misfolded oligomerized amyloid-β (Aβ) protein accumulate in both dense mesoscale extra-cellular plaques and intracellular fibrils. In addition, hyper-phosphorylated microtubule-associated tau (p-tau) cytoskeletal protein aggregates into dense intra-neuronal neurofibrillary tangles (NFTs). Moreover, cellular systems that regulate protein folding (e.g., chaperone proteins) and degradation (e.g., proteasomal and lysosomal systems) are also known progressively become altered in brain cells during aging [3] (e.g., in lipofuscin granula, see Suppl. Introduction).

An in-depth characterization of AD neurodegeneration has been achieved using established immunohistochemistry (IHC) approaches [4], and extensive PET-tracer development is helping effective differential diagnosis [5]. Still, modern imaging tools come short of delivering the volumetric cellular-level visualizations, which would be necessary to reliably detect and differentiate individual subtle protein-based cellular lesions or abnormal age-related intracellular accumulations in vivo, especially in the likely crucial pre-symptomatic early phases of AD. Moreover, brain-wide cellular-level monitoring of cellular aging and AD neurodegeneration progression is especially challenging for neuroimaging. An unequivocal AD diagnosis still can only be reached postmortem via histopathologic demonstration of the two hallmark protein lesions (Aβ and p-tau) in nervous system tissue. And even in the context of postmortem small animal imaging, current cutting-edge experimental neuroimaging methods lack an unbiased high-throughput 3D imaging technology sensitive to neuronal proteopathy that allows cellular resolution, full-organ brain coverage, and unbiased detection mechanisms (see Suppl. Introduction). This limited diagnostic power significantly hinders our monitoring of normal neuronal aging processes, and especially our understanding of early-stage AD etiology and our ability to discover disease-modifying drugs for AD.

Amongst novel neuroimaging techniques, X-ray phase-contrast computed tomography [6] (X-PCI-CT) represents a set of 3D microscopy techniques, offering enhanced image contrast compared to traditional absorption CT [7] and thereby enable label-free soft-tissue imaging for diverse biomedical investigations [8]. Its simplest implementation, propagation-based X-PCI-CT [9, 10], exploits the physical mechanisms of coherent X-ray refraction, propagation, and Fresnel diffraction to determine local electron density within probed samples. Applied postmortem, different X-PCI-CT methods deliver semi-quantitative to fully quantitative label-free and dissection-free density-based 3D morphological neuroimaging complementary to other brain mapping techniques [11–13]. Modern synchrotron-radiation setups reach the spatial resolution needed to impact mesoscale neuroimaging [14–17], permitting the volumetric exploration of intracellular cyto-architecture within single neurons in deep rodent brain regions [13, 18, 19]. Furthermore, they can be used to carry out unbiased anatomically dense high-throughput quantifications of cellular and vascular structure within large nervous-tissue samples at histological resolution [20–22]. With regard to the detection of neurodegeneration, X-PCI-CT was shown to be sensitive to extra-cellular Aβ build-up in several AD animal models postmortem [23–29]. Still, previous studies have come short of a multiscale and multimodal characterization of early-stage Aβ and p-tau-driven NFT build-up in intracellular compartments. Furthermore, prior studies have fallen short of applying X-PCI-CT within experimental neuroscience protocols.

Here, we present a postmortem X-PCI-CT-based multiscale organ-level to cellular-level analysis of brain cellular hyperdensity, focusing on intracellular abnormal protein and metal accumulation in aging and AD animal models. We performed micro-X-PCI-CT to nano-X-PCI-CT and X-ray fluorescence microscopy on extracted brain samples from aged wild-type (WT) and triple-transgenic 3xTgAD mice [30], an experimental AD model that develops both amyloid and tau pathology [31]. After analyzing multiscale X-PCI-CT results by comparison to more-established neuroimaging modalities, including MRI, TEM, and immunohistochemistry (IHC), we used this methodology to quantify differences in intracellular hyperdensity within key AD-linked hippocampal and cortical brain cell layers.

Neuroprotective effects, which counteract the degeneration in aging and diseased neuro populations, are being intensely investigated. Group II metabotropic glutamate (mGlu2 and mGlu3) receptors, for example, are interesting potential pharmacological targets, as they do not mediate but modulate glutamatergic neurotransmission. These receptors are located in presynaptic terminals where they negatively modulate adenylate cyclase with ensuing inhibition of glutamate release [32]. mGlu3 receptors are also expressed in astrocytes, and activate mitogen-activated protein kinase and phosphatidylinositol-3-kinase pathways with ensuing increased production of neurotrophic factors such as transforming growth factor-β, glial-derived neurotrophic factor, nerve growth factor, and S-100β protein [33]. Activation of astrocytic mGlu3 receptors also upregulates the expression of the glutamate transporter 1, which contributes to a reduction in extra-cellular glutamate concentrations, thus limiting excitotoxicity [34]. Here, we tested the hypothesis that chronic pharmacological activation of mGlu2/3, by using the selective orthosteric agonist LY379268 [35], could exert a neuroprotective effect in 3xTgAD mice. By collecting X-PCI-CT data from aged WT and 3xTgAD mice, systemically treated with saline or LY379268, we demonstrated for the first time a proof-of-principle application of X-PCI-CT for the morphologic and quantitative evaluation of local and intracellular neurodegeneration/neuroprotection processes.

## Method summary

A full description of methodology can be found in the Suppl. Methods section.

### Ethical compliance

All experimental procedures on animals were performed following national and international regulations.

### Animals

Eight symptomatic 11-month-old male B6/129 wild-type (WT) mice (Charles-River Laboratories) and seven male 3xTgAD [30] mutant mice (B6;129-Tg (APPSwe, tauP301L) 1Lfa *Psen1*^*tm1Mpm*^/Mmjax, Stock No: 34830-JAX) were treated with either saline or the selective agonist of group II metabotropic glutamate receptors LY379268 by means of subcutaneously implanted osmotic mini-pumps (Alzet) for 28 days (continuous rate of 1 mg/kg/day). One month after treatment, all mice were sacrificed, and their brains dissected, fixed in Carnoy solution overnight, and embedded in paraffin. One asymptomatic 4-month-old WT mouse was included as a young animal control.

### X-PCI-CT acquisitions

3D postmortem multiscale X-PCI-CT was performed on mouse half brain samples embedded in minimal paraffin. The 3.0^3^, 0.7^3^, and 0.3^3^ µm^3^ effective voxel size CT scans were performed on unsectioned samples in a PBS bath within cylindrical plastic tubes. The 0.1^3^ µm^3^ voxel scans were performed in air on ~ 2 × 2 × 4 mm^3^ tissue biopsies of CTX and HIP brain areas. The 3.0^3^ µm^3^ voxel size was chosen to image the full volume of a mouse brain. The 0.3^3^ µm^3^ and 0.1^3^ µm^3^ voxel sizes were selected to visualize respectively cellular and sub-cellular size features, and the 0.7^3^ µm^3^ to obtain an intermediate view of tissue-level morphology.

The 3.0^3^ and 0.7^3^ µm^3^ voxel micro-X-PCI-CT data were collected via single-distance propagation-based imaging (PBI) setups of the ID17 Biomedical beamline [14] of the ESRF, using a 30 keV X-ray beam or a pink X-ray beam with peak at 40 keV and a ~ 20 keV broad spectrum, respectively. Both setups used a sCMOS PCO.Edge 5.5 (PCO AG, Germany) detector and a YAG-based scintillator, coupled to a × 1:2 optic system to obtain the 3.0^3^ µm^3^ effective voxel size, and a × 1:10 optic system to obtain the 0.7^3^ µm^3^ effective voxel size.

The 0.3^3^ voxel micro-X-PCI-CT data were collected via the single-distance PBI imaging setup of the TOMCAT beamline [15, 36] of the Swiss Light Source using a 21-keV X-ray beam, a sCMOS PCO.Edge 5.5 detector, and a 20-µm-thick LuAG:Ce scintillator, coupled to an Optique Peter microscope at 20 × magnification.

The 0.1^3^ voxel nano-X-PCI-CT data were collected via the X-ray nano-holotomography (XNH) imaging setup of the ESRF ID16A beamline [13, 16, 17, 37] using a 17-keV X-ray beam and a CCD camera (FReLoN, ESRF) at a fixed focal-plane-to-detector distance of ~ 1.2 m, coupled to magnifying optics and a 23-µm-thick GGG:Eu scintillator.

X-PCI-CT scan parameters are listed in the Suppl. Methods section.

### X-PCI-CT data processing

Micro-CT data tomographic reconstructions were obtained via filtered-back projection after application of Paganin’s single-distance phase-retrieval algorithm [38]. XNH data was reconstructed after application of a 4-distance contrast-transfer-function-based algorithm for phase-retrieval [39, 40], including Wiener regularization [41]. Cupping artifacts were removed via normalization, ring artifacts via an ESRF in-house post-processing tool [42]. Maximum intensity projections (MIPs) were computed by summing 20–100 consecutive CT images via the z-projection function in ImageJ [43]. 3D renderings were obtained using the commercial software VG Studio Max 3.2 (Volume Graphics GmbH).

### X-PCI-CT data quantification

Automatic threshold-based segmentation (maximum entropy auto-threshold algorithm [44] in ImageJ) of hyperdense (HD) particles in X-PCI-CT data was applied to systematically quantify cell-like HD within ventral and dorsal HIP and CTX volumes-of-interest (VoI) in 4 experimental animal groups (13 months old): (1) WT mice treated with saline (*n* = 4), (2) WT mice treated with LY379268 (*n* = 4), (3) 3xTgAD mice treated with saline (*n* = 3), and (4) 3xTgAD mice treated with LY379268 (*n* = 4). VoI were analyzed via the “3D Object Counter” ImageJ plugin [45] and the HD particle 3D tissue load was computed as the ratio between HD volume over total volume. Extracted particle size distributions were fitted with a 3-component Gaussian mixture model (fitgmdist function in MATLAB R2018a, The MathWorks, Inc.) to compute the mean sizes of each of the three cell populations (nucleoli of normal cells, glial cells, HD cells), as well as population proportions within the VoI. 90 virtual-histological 3D tissue VoI were analyzed via this automated analysis pipeline.

### XFM measurements

Half brain samples were sliced into 6-µm-thick coronal sections with a Leica RM2265/LN22 ultra-microtome (Leica Microsystems) and loaded onto 7 × 7 mm^2^ silicon nitride window frames. 2D raster scanning nano-XFM measurements were performed in vacuum at room temperature conditions using the setup of the ID16A beamline [17, 46, 47] and a 17-keV X-ray beam. Quantitative elemental area density maps for K, S, P, Zn, Fe, Cu, Ca, and Br (in ng/mm^2^) were obtained from individual point spectra by fitting and normalizing the data using the PyMCA-software for XFM spectral analysis [48]. Mean intracellular 2D elemental density within 22 “normal” and 11 “ICHD-bearing” cells (labels assigned by manual inspection of morphology and cell gray levels in phase maps) was measured after manual selection of intracellular areas in normalized and calibrated XRF maps.

### High-field MRI measurements

A preclinical 9.4-T MRI scanner (BioSpec 94/21 USR; Bruker) was used for MRI data collection on half brain samples first embedded in 2% agarose only (non-contrast-enhanced), and then following a contrast-enhancement approach [49] based on incubation with Dotarem (0.5 mmol Gd/mL; Guerbet). Coronal brain images were collected using a transceiver cryogenic quadrature RF surface probe and 3D FISP sequences.

### TEM measurements

The 2 × 2 × 4 mm^3^ biopsies were dehydrated, substituted with a mixture of resin and ethanol, and embedded in Embed 812 resin (EPON substitute, EMS). Ultrathin sections (~ 200 nm) were cut with a Leica EM UC7 ultra-microtome (Leica Microsystems), collected on formvar-coated copper grids, and counterstained with uranyl acetate 2% and lead citrate 2%. Images were obtained using a Tecnai12 G2 Spirit Bio Twin (ThermoFisher) microscope operating at 120 kV and an Orius SC1000 CCD camera (Gatan).

### Immunohistochemistry

The 10 µm coronal sections were cut on a microtome and stained with Thioflavin-S (Sigma-Aldrich). For immunohistochemistry, sections were deparaffinized, rehydrated by standard procedures, and incubated with the following primary antibodies: anti-β-amyloid 17–24 clone 4G8 made in mouse (1:100; Biolegend; RRID: AB_2564633), anti-phospho-tau pSer404 made in rabbit (1:100, Sigma-Aldrich; RRID: AB_261759), anti-NeuN made in rabbit (1:200, Millipore; RRID: AB_10807945), and anti-NeuN made in mouse (1:200, Millipore; RRID: AB_2298772) for double immuno-labeling. Various conjugated secondary antibodies were applied, and slices, mounted with Vectashield with Dapi (Vector Laboratories), were examined under a Zeiss 780 confocal laser scanning microscope.

### Statistical analysis

All statistical analyses were performed using the SAS software (SAS Studio 2.7, SAS Institute Inc.). All graphs were then produced with GraphPad Prism (GraphPad Prism 8.0.0, GraphPad Software Inc.).

## Results

The study was designed in three consecutive parts, as schematized in Suppl. Figure 1.

### Part I

#### X-PCI-CT detects intracellular hyperdensity in aged WT and aged 3xTgAD mouse brains

Using unsectioned paraffin-embedded half brain samples and a 0.3^3^ µm^3^ voxel single-distance propagation-based X-PCI-CT setup [15], we analyzed cortical (CTX) and hippocampal (HIP) regions in the brains of one 4-month-old WT mouse, of two 13-month-old WT mice, and of two 13-month-old transgenic 3xTgAD mice. To better access intracellular detail, ~ 2 × 2 × 4 mm^3^ brain tissue biopsies, cut out from the original half brain samples, were additionally analyzed with a 0.1^3^ µm^3^ voxel X-ray nano-holotomography setup [37].

**Fig. 1:**
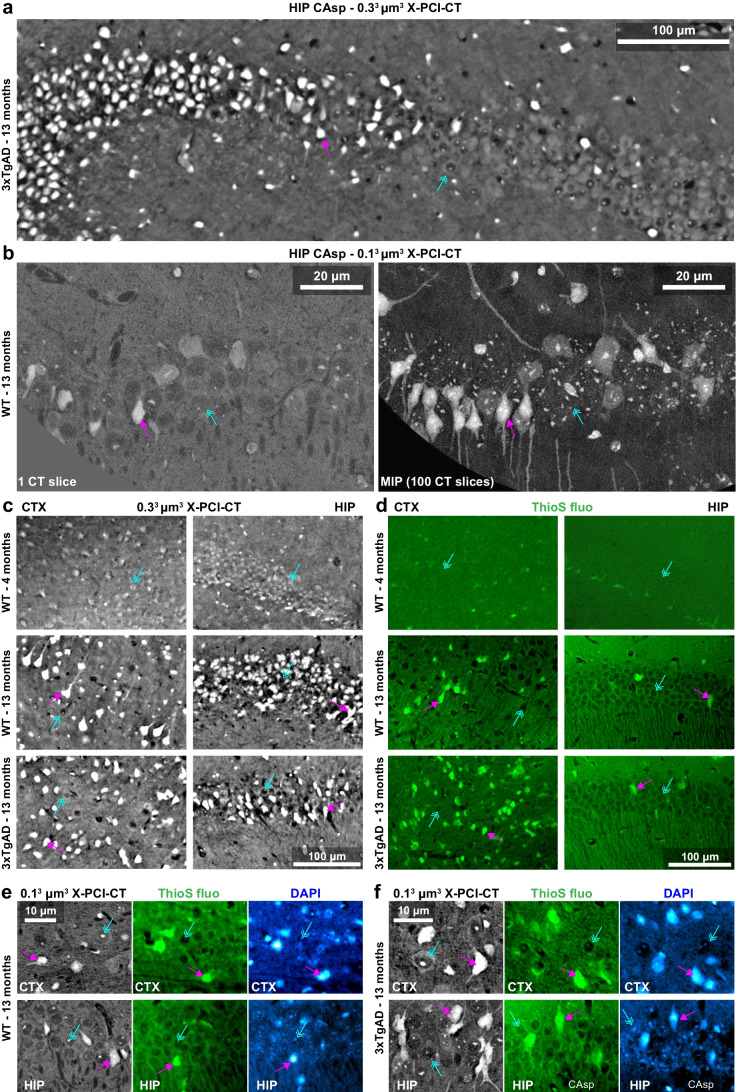
X-PCI-CT-detected hyperdensity matches intracellular Thioflavin-S fluorescence. Representative **a** 0.3^3^ µm^3^ voxel and **b** 0.1^3^ µm^3^ voxel 3D X-PCI-CT (including a 100-slice maximal intensity projection/MIP) of Ammon’s horn hippocampal pyramidal neurons in aged 13-month-old 3xTgAD and WT mice. **c** 0.3^3^ µm^3^ voxel X-PCI-CT of deep cortical (CTX) and hippocampal (HIP) layers within extracted paraffin-embedded brain samples from a 4-month-old WT mouse vs. a 13-month-old WT vs. a 13-month-old 3xTgAD mouse. In **a**-**c**, bright image gray levels represent hyperdense (HD) tissue areas, while dark gray levels represent hypodense tissue areas. Compared to normal nearby neurons (azure arrows), HD cell-shaped particles (magenta arrows) are present in the 13-month-old WT and 3xTGAD samples. Few to no HD particles are visible in tissues at 4 months (**c**). **d** Thioflavin S fluorescence microscopy (ThioS fluo) data of CTX and HIP tissues comparable to the ones in **c**, collected from contralateral brain hemispheres. **e**–**f** 0.1^3^ µm^3^ voxel CTX and HIP X-PCI-CT data vs. ThioS and DAPI fluorescence data from contralateral CTX and HIP tissue samples, collected from 13-month-old **c** WT and **d** 3xTgAD animals. In **e**–**f**, same-cell co-localization of ThioS and DAPI fluorescence can be clearly observed, suggesting intracellular localization of the ThioS-positive deposits. In **c** vs. **d** and in **e**–**f**, ThioS-positive deposits (magenta arrows) in fluorescence datasets show morphological traits and distribution patterns similar to the patterns of HD particles (magenta arrows) in X-PCI-CT images, whereas ThioS-negative cellularity (azure arrows) in fluorescence datasets matches normal hypodense cellularity (azure arrows) in X-PCI-CT images. Small intracellular nucleoli are marked by the ThioS dye and appear as intra-nuclear HD features in X-PCI-CT maps

**Fig. 2:**
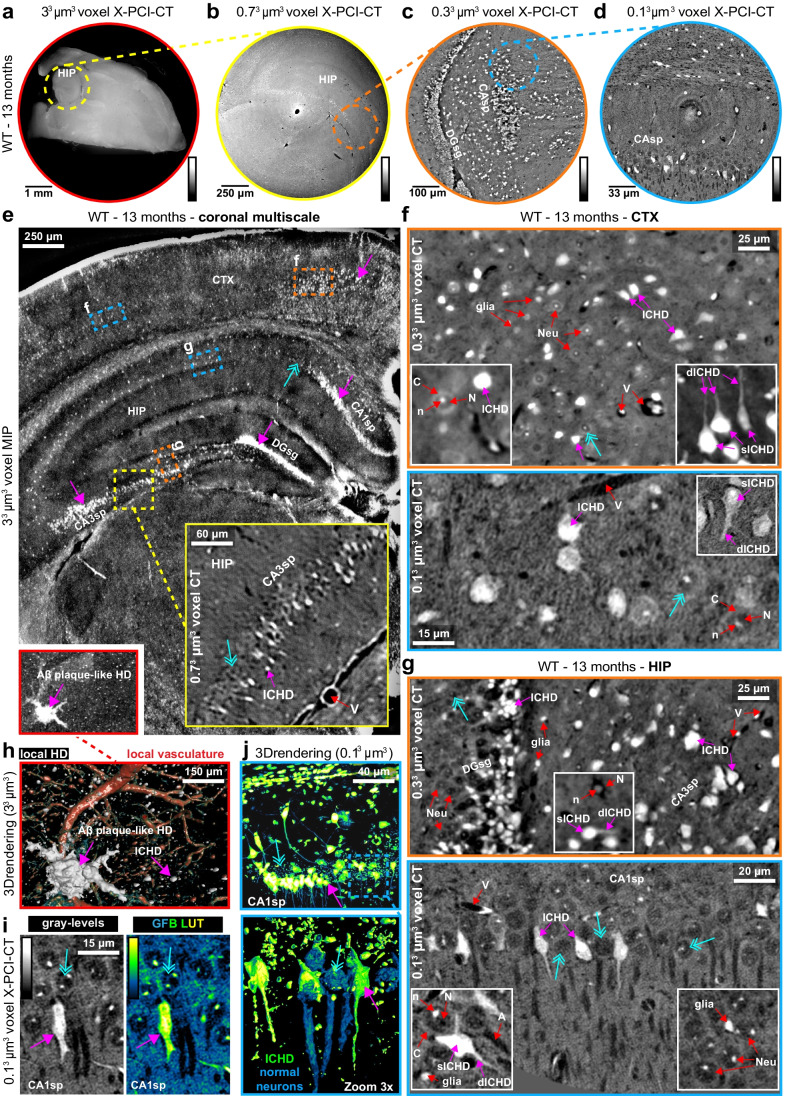
Multiscale X-PCI-CT of brains from aged WT mice. **a**-**d** Organ-level to cellular-level X-PCI-CT neuroimaging of the brains of 13-month-old WT mice, at increasingly smaller effective voxel sizes: **a** 3.0^3^, **b** 0.7^3^, **c** 0.3^3^, and **d** 0.1^3^ µm^3^. Lower pre-cellular resolution CTs were used to aim the higher-resolution ones. The latter allowed cellular-level explorations of angio-architecture and cyto-architecture within selected deep brain regions. Calibration bars specify low-to-high gray-level encoding of tissue density. Brain samples underwent dissection into ~ 2 × 2 × 4 mm^3^ rods before 0.1^3^ µm^3^ voxel X-PCI-CTs in **d**, **f**-**g**. **e** 3.0^3^ µm^3^ voxel coronal maximal intensity projection (MIP), enhancing the visibility of intracellular hyperdensity (ICHD) within neuronal populations in CTX, HIP CA1sp/CA3sp, and DGsg layers. **f**-**g** 0.3^3^ and 0.1^3^ µm^3^ voxel X-PCI-CTs of **f** CTX and **g** HIP layers visualize deep vasculature (V, hypodense), individual normal neurons (Neu), non-neuronal glial cells (glia), and intra-neuronal structure (cytoplasm (C), hypodense nuclei (N), hyperdense nucleoli (n), hypodense axons (A)). ICHD-bearing neurons contain somatic (sICHD) and dendritic/axonal (dICHD) ICHD. **h** 3D rendering of a macroscopic HD object with amyloid (Aβ) plaque-like morphology from **e**, alongside ICHD (white) and vasculature (red). **i** Gray-level coloring vs. fluorescence-like green-fire-blue (GFB) LUT recoloring of 0.1^3^ µm^3^ voxel X-PCI-CT data, highlighting a single ICHD-bearing CA1sp neuron within a population of normal neurons. **j** GFB-recolored 3D rendering of CA1sp ICHD-bearing neurons. Zoom 3 × portrays individual ICHD-bearing (green) vs. normal (blue) neurons. In **e**-**j**, magenta arrows point to example of ICHD, azure arrows to normal neurons

Interestingly, the collected 3D imaging data (Fig. 1a-c, Suppl. Fig. 2) showed widespread cell-shaped hyperdense (HD) particles within CTX and HIP layers in the brains of the 13-month-old animals. HD particles were also observed in the same neuronal layers of adjacent hypodense neuron-like structures, and their prevalence varied greatly between tissue locations (Suppl. Figure 3). By contrast, no HD particles were observed in the brain of the young 4-month-old WT mouse (Fig. 1c). Preliminary characterization of the biological content within HD particles was obtained by side-by-side comparison of the X-PCI-CT data with fluorescence microscopy of Thioflavin S (ThioS)-stained tissue sections collected from contralateral hemispheres (Fig. 1). ThioS fluorescence, which nonspecifically highlights cross-β-sheet architecture within both amyloid and p-tau protein lesions [50–52], showed strongly ThioS-positive cell-shaped clusters within sections from 13-month-old mice, whereas only very low to no ThioS signal in sections from 4-month-old WT mice. Patterns of ThioS fluorescence tissue marking appear analogous to HD particle distributions in X-PCI-CT density-maps (Fig. 1c vs. 1d, e–f, Suppl. Figure 2), suggesting that HD particles may contain Aβ and p-tau protein deposits, especially in 3xTgAD model tissues. The observation that ThioS fluorescence co-localizes with DAPI fluorescence (Fig. 2c-f, Suppl. Figure 2) suggests that the ThioS signal, and by extension the HDs observed in the X-PCI-CT data, originate from within intracellular compartments. These observations suggest that the presence of intracellular HD particles (ICHD) within CTX and HIP tissues from both WT and 3xTgAD mouse brains at 13 months might be related to aging or neurodegenerative processes.

### Part II

#### Multiscale X-PCI-CT map organ-level to cellular-level intracellular hyperdensity in aged brains

To systematically localize and characterize intracellular hyperdensity (ICHD) within mouse half brain samples, we applied a multiscale approach to X-PCI-CT-based neuroimaging: Consecutive same-sample X-PCI-CT experiments at increasing spatial resolution were performed using different synchrotron-radiation setups. We acquired datasets with effective voxel sizes of 3.0^3^, 0.7^3^, 0.3^3^, and 0.1^3^ µm^3^ (see the “Method summary” section), a sequence of measurements, which permitted organ-level inspection of tissue-level 3D structure followed up by local cellular-level examinations of deep single-neuron 3D morphology (Fig. 2a, Suppl. Figure 4, Suppl. Videos 1–4).

**Fig. 3:**
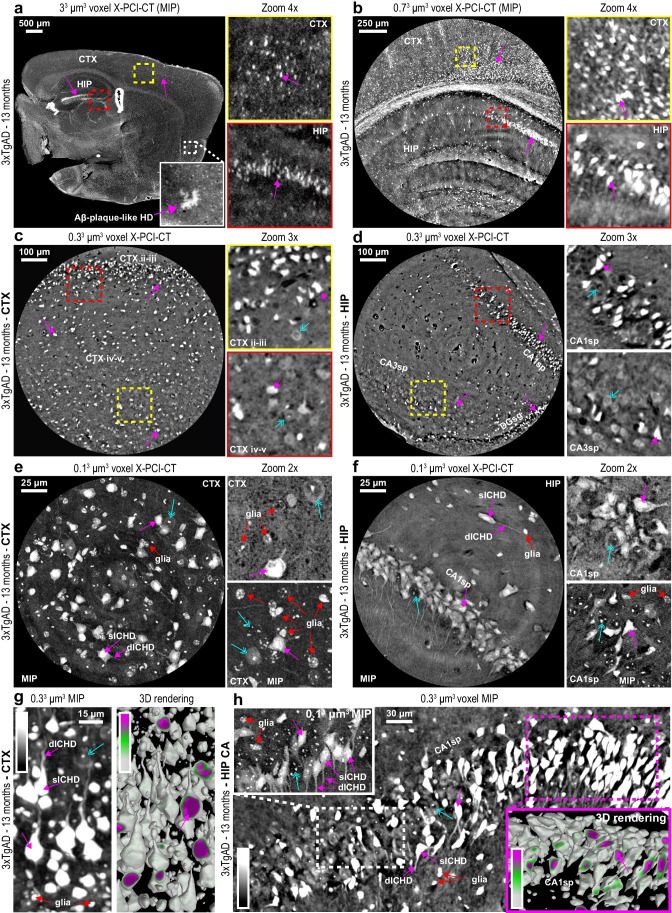
Multiscale X-PCI-CT of brains from aged 3xTgAD mice. **a**-**f** Organ-level to cellular-level X-PCI-CT neuroimaging of the brains of 13-month-old 3xTgAD mice, at increasingly smaller effective voxel sizes: **a** 3.0^3^, **b** 0.7^3^, **c**-**d** 0.3^3^, and **e**–**f** 0.1^3^ µm^3^. Note widespread ICHD within **a**-**b** cortical (CTX) and hippocampal (HIP) layers, preferential deposition in **c**-**d** ii^nd^-iii^rd^ and iv^th^-v^th^ CTX and CA1sp layers, and some deposition in HIP DGsg and CA3sp layers. Sub-cellular datasets (**e**–**f**) visualize individual ICHD-bearing cells with bright possibly condensed somas (sICHD), and bright dendritic/axonal components (dICHD), compared to more hypodense somas and small very dense intra-nuclear particles (nucleoli) within normal neurons. Intermediate size mildly HD glial cellular populations (glia) are also visible. **g**-**h** 0.3^3^ and 0.1^3^ µm^3^ voxel MIP maps and 0.3^3^ µm^3^ voxel 3D renderings, obtained after threshold-based gray-level segmentation, of **g** CTX and **h** HIP CA1sp layers. Note evident dendritic and axonal ICHD involvement in hyper-intense branch-like extensions (dICHD) of ICHD somas (sICHD). 3D renderings demonstrate that the highest gray-level voxel values (rendered magenta, see calibration bars), and thus the highest local densities, can be found within internal cellular compartments of ICHD-bearing neurons, and especially cytoplasmic and nuclear one. Normal neuron somas, having intracellular gray levels lower than the chosen threshold, are not rendered in 3Ds in **g**-**h**. In **a**-**h**, differently shaped ICHD-bearing pyramidal and bipolar neurons can be recognized, magenta arrows point to example of ICHD, and azure arrows to normal neurons

Overall, we obtained multiscale neuroimaging datasets on fifteen brain hemispheres extracted from eight WT (selected results in Fig. 2) and seven 3xTgAD mice (selected results in Fig. 3), all aged to 13 months either under control conditions or after treatment with LY379268. The 3.0^3^ µm^3^ voxel maps were used to measure gross pre-cellular neuroanatomy within entire half brain samples without sectioning of tissue (Figs. 2a, 3a), which served as scout images for subsequent higher-resolution CT acquisitions. Local-tomography 0.7^3^ and 0.3^3^ µm^3^ voxel scans (Figs. 2b-c, 3b-d), also performed dissection-free, were used to bridge the spatial resolution gap between pre-cellular and cellular-level investigations. After a sample dissection step, 0.1^3^ µm^3^ voxel nano-holotomography was performed on tissue biopsies to probe sub-cellular-scale data in dorsal CTX and HIP layers (Figs. 2d, 3e-f). Higher-resolution acquisitions led not only to increasingly smaller volumes of brain tissue being measured, but also to increasingly more detailed visualizations of the 3D cyto-architecture, of deep pyramidal and granular neurons supplied by their local microvasculature, of bipolar HIP interneurons [53], and of different glial populations. Via this multiscale approach, we could target key brain regions in AD pathology [54], e.g. the CTX (Figs. 2e-f, 3c, 3e, Suppl. Videos 5–6) and HIP (Figs. 2e, 2g, 3d, 3f, Suppl. Videos 7–8), as well as specific layers, e.g. pyramidal-neuron layers within Ammon’s horn (CA1sp/CA3sp) and granulate-neuron layers within the dentate gyrus (DGsg).

In 13-month-old WT mice, cell-shaped ICHD was observed in specific brain layers (Fig. 2e-g), as well as very few macroscopic extra-cellular HD deposits with plaque-like morphology (Fig. 2e, h), likely due to physiological brain aging. The acquired cellular resolution data allowed the visualization of individual hyperdense cells and the recognition of sub-cellular density variations (Fig. 2f-g): ICHD-bearing neurons appeared filled with—likely protein-rich—condensation products, and HD was located in both cell somas (sICHD) and dendritic/axonal compartments (dICHD). Nearby normal neurons were instead distinguishable due to their comparatively average-density cytoplasms, spherical hypodense—likely euchromatin-rich—nuclei, small hyperdense nucleoli, and hypodense axons. By pseudo-fluorescent recoloring of 2D X-PCI-CT slices (Fig. 2i) and 3D renderings (Fig. 2j) of ICHD-bearing cell layers, we emphasized the similarity between an X-PCI-CT-based nonspecific detection of ICHD bio-deposits to ThioS fluorescence-based nonspecific detection of protein clumping. ICHD in WT mice are likely due to physiological aging processes.

In all 3xTgAD brains, sparse macroscopic extra-cellular HD aggregates with 3D-morphology reminiscent of Aβ-plaques (Fig. 3a) could be recognized alongside much smaller and widespread cell-shaped HD. The detected HD was predominantly intracellular (ICHD), in line with the description of the 3xTgAD mouse model reported in its foundational papers [30, 31]. Unlike other models of AD (e.g., the APP/PS1), intracellular Aβ accumulation should be the earliest (starting at 6 months) and preponderant pathological manifestation in 3xTgAD mice, while later on (e.g., at 15 months) both hippocampal and coronal tissues should start presenting evident extra-cellular Aβ plaques and tau pathology. In this work, the focus was set on the characterization and quantification of the intracellular deposits, e.g. on early-onset alterations. Highlighted via maximal intensity projections (MIP) of consecutive CT slices, the ICHD presented spatial patterns and morphology typical of AD-linked intracellular protein accumulations (Fig. 3a-f, Suppl. Figure 5): ICHD in 3xTgAD mice was, in fact, preferentially deposited within neurons of notoriously AD-linked locations, including CTX’s ii^nd^-iii^rd^ and iv^th^-v^th^ layers and the ENT layer ii Fig. 3a-c, as well as HIP CA1sp/Ca3sp and DGsg layers (Fig. 3d). In the 0.1^3^ µm^3^ voxel datasets (Fig. 3e-f, Suppl. Figure 6), glial involvement in the vicinity of ICHD cells could be resolved, and the presence of different glial populations was observed: High-density glial cells likely pertain to oligodendrocytes and microglia, denser due to their characteristic nuclear chromatin clumps, and low-density ones, likely astrocytes with pale irregularly shaped somas. These contrast patterns match known glia density-based contrast patterns traditionally visible via light microscopy in toluidine-blue-stained semi-thin sections [55, 56].

To exploit the volumetric nature of the collected data, easy-to-implement mixed semi-automatic threshold-based and manual region-growing segmentation approaches (Suppl. Figure 7) were applied to selectively extract and 3D-visualize ICHD-bearing cells, normal neurons, and hypodense vasculature within extended tissue volumes (Figs. 2–3, Suppl. Figures 4, 7). Compared to hypodense nearby normal neuron parenchyma, 3D renderings of ICHD-bearing multi-polar (pyramidal) neurons in HIP (Figs. 2j, 3g) and CTX (Fig. 3h) layers portray near-nuclear cytoplasmatic somatic compartments as the densest intracellular locations, and evident involvement also of dendritic, axon-hillock, and axonal compartments.

Even though neurons presenting ICHD were observed in both aged WT and aged 3xTgAD brain samples (Figs. 2–3), morphological comparison of equivalent CAsp-layer neurons in one WT vs. one 3xTgAD brain sample showed evident morphological differences between ICHD cells in the two different animal types (Suppl. Fig. 8). Specifically, widespread cyto-structural signs typical of cellular neurodegeneration leading to cell death (i.e., somatic cell-blebbing and shrinkage) were only visible in the data pertaining to the 3xTgAD mouse, whereas somatic ICHD in WT mice appeared well-rounded or pyramidal and thus more likely related to normal neuronal aging processes (more in Suppl. Results).

#### ICHD correlates to Aβ and p-tau IHC, i.e. to cellular neurodegeneration, in 3xTgAD mice

**Fig. 4:**
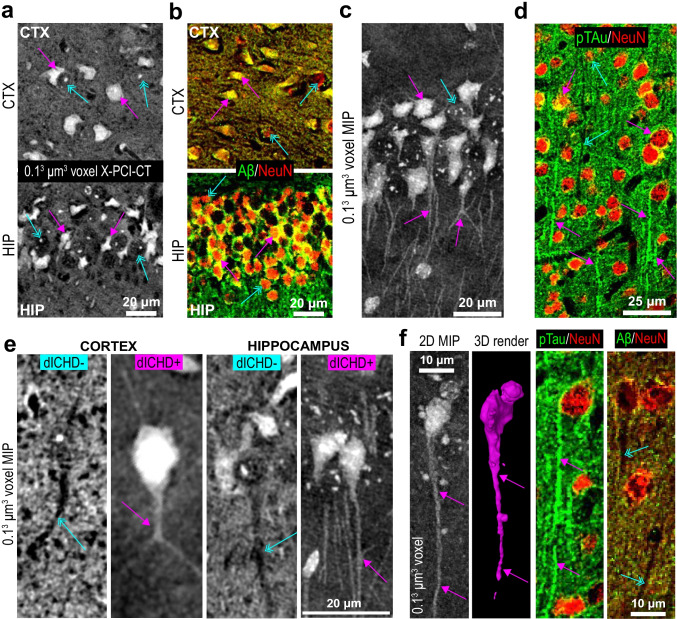
Label-free X-PCI-CT detection of ICHD vs. IHC for Aβ and p-tau. **a**-**b** Representative **a** X-PCI-CT data vs. **b** contralateral-hemisphere doubly stained Aβ/NeuN IHC (anti-β-amyloid 4G8 fluorescence in green, anti-NeuN in red), of comparable CTX/HIP tissues from 13-month-old 3xTgAD mice. Note somatic co-localization of Aβ-positive and NeuN-positive signals (yellow pixels in **b**), forming cytoplasmic ring-like patterns, but no dendritic/axonal Aβ-positive signal. Somatic Aβ deposition (in **b**) resembles X-PCI-CT-detected somatic IHCD (in **a**). Non-ICHD-bearing normal cells in **a** are similar to Aβ-negative cell somas in **b**. **c**-**d** Representative **c** X-PCI-CT MIPs vs. **b** contralateral-hemisphere doubly stained p-tau/NeuN IHC (anti-p-tau pSer404 fluorescence in green, anti-NeuN in red), of comparable tissues from 13-month-old 3xTgAD mice. Note little somatic co-localization of p-tau- and NeuN-positive signals (yellow pixels in **d**), whereas high dendritic/axonal p-tau-positive (green) fluorescent signal in long branch-like structures extending from the mildly p-tau-positive and NeuN-positive cell somas. Axonal p-tau deposition resembles X-PCI-CT-detected axonal ICHD out-branching from somatically ICHD-bearing cells. **e** Hyperdense (dICHD^+^) vs. hypodense (dICHD^−^) dendritic/axonal processes observed via nano-X-PCI-CT in 13-month-old 3xTgAD CTX and HIP tissues. dICHD^+^ processes connect to somatically ICHD-bearing cells, dICHD^−^ processes to normal hypodense cell somas. **f** 2D MIP vs. 3D rendering of a neuron bearing a dICHD^+^ axon extending from its ICHD^+^ cell soma. IHCs show respectively examples of p-tau-positive and Aβ-negative axons. In **a**-**f**, magenta arrows point to example of either ICHD-bearing or Aβ/p-tau-positive somatic and axonal cell compartments, azure arrows to either normally dense non-ICHD-bearing or Aβ/p-tau-negative somatic and axonal cell compartments

To confirm the biological nature and the cellular localization of the ICHD aggregates detected via X-PCI-CT, fluorescence (ThioS) and IHC (primary antibodies anti-Aβ 17–24 clone 4G8, anti-p-tau pSer404, and anti-NeuN) were collected on CTX and HIP tissues of contralateral hemispheres (Fig. 1, Fig. 4, Suppl. Figure 9). ThioS fluorescence showed strongly ThioS-positive cell-shaped clusters in CTX and HIP tissues of 13-month-old 3xTgAD mice, similar but less widespread staining patterns in WT animals of the same age, and very low or no ThioS staining in the same brain regions in 4-month-old WT and 3xTgAD mice (Suppl. Figure 9a-b). Immunoreactivity for Aβ was highly detectable in the cerebral cortex and hippocampus of 13-month-old 3xTgAD mice, and double IHC for Aβ and NeuN showed intra-neuronal perisomatic cytoplasmic Aβ deposition in 3xTgAD mouse brains (Suppl. Figure 9c-d). No sign of axonal Aβ fluorescence was visible. Immunoreactivity for p-tau was detectable only in the cerebral cortex of 13-month-old 3xTgAD mice, with the double IHC for p-tau and NeuN showing a very low immunoreactivity in the neuronal soma, but stronger p-tau-immunoreactivity within neuron dendrites and axons (Suppl. Figure 9e). Only a very low level of immunoreactivity for Aβ was observed in WT mouse brains.

Direct comparison of cellular-level X-PCI-CT data to the Aβ and p-tau IHC (Fig. 4) showed good morphological overlap between the two signals (more in Suppl. Results). The intra-neuronal perisomatic cytoplasmic Aβ amyloid deposition in 3xTgAD cells resembles the X-PCI-CT-detected somatic ICHD (Fig. 4a-b) and the p-tau IHC patterns match the ICHD observed via X-PCI-CT in dendritic and axonal compartments of somatically dense neurons (Fig. 4c-f). The collected IHC thus suggests that in 13-month-old 3xTgAD mice the dense cell-like particles likely correspond to intracellular Aβ deposits and p-tau agglomerations. The detected low levels of somato-dendritic ICHD and ThioS fluorescence in the 13-month-old WT mice instead likely arise due to normal neuron aging processes, such as lipofuscin granula formation (notoriously auto-fluorescent).

#### X-PCI-CT vs. X-ray fluorescence microscopy, MRI, and TEM

**Fig. 5:**
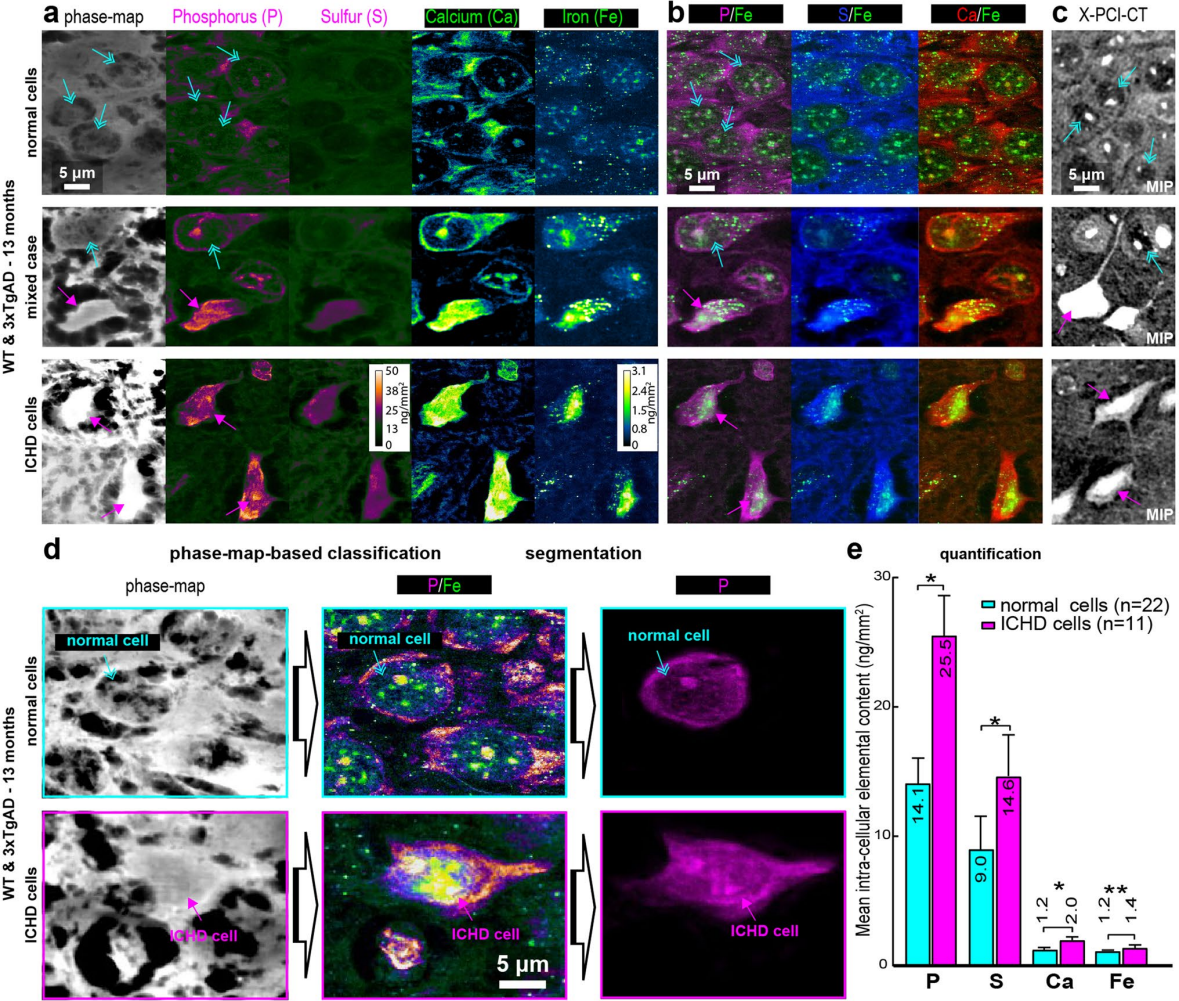
X-PCI-CT vs. XFM. **a** Recolored cellular XFM data pertaining to 6-µm-thick CTX and HIP brain sections representative of 13-month-old both WT and 3xTgAD mice (calibration bars in ng/mm^2^). Maps visualize intracellular phase and elemental distributions for phosphorus (P) and sulfur (S), calcium (Ca), and iron (Fe). **b** Co-localization XFM maps of the same cells as in **a**, with P, S, or Ca distributions alternatively superimposed to Fe distributions. **c** MIPs of 0.1^3^ µm^3^ voxel X-PCI-CTs, showing deep neuronal cell groups, with intracellular morphology and density patterns, which morphologically match intracellular patterns in the XFM-measured cell groups (**a**-**b**). In **a**-**b** vs. **c**, magenta arrows point respectively to elemental hyper-accumulation or ICHD-bearing neurons, azure arrows to normally compartmentalized low-density elemental accumulations or normal non-ICHD-bearing neurons. **d** 33 neurons within the XFM maps were manually classified, based on the morphology and intensity visible in their phase maps, as either ICHD-bearing (*n* = 11, magenta arrows) or normal (*n* = 22, azure arrows) neurons. Representative phase, P/Fe co-localization, and segmented P XFM maps of two cells are shown here as demonstration. Each classified cell was manually segmented, and masks were used to measure average intracellular elemental content within each cell. **e** Quantification of XFM-measured intracellular elemental content in ICHD-bearing (magenta) vs. normal (azure) neurons within one WT and one 3xTgAD animal at 13 months. Graph shows group mean ± SD. All elemental distributions (P, S, Ca, Fe) show significant differences in mean intracellular elemental content (*p < .001, **p < .005) between the two cell groups, compared by unpaired two-sided two-sample Wilcoxon testing

To evaluate the elemental composition of the intra-neuronal ICHD deposits detected via X-PCI-CT, we performed nano-scale X-ray fluorescence microscopy [57] (XFM, see the “Method summary” section) on 6-µm-thick histological sections from one WT and one 3xTgAD brain sample. We collected 2D phase maps and 2D trace-element distribution maps within cells, quantifying key bio-elements, including phosphorus, sulfur, calcium, and iron. These measurements covered tens of individual neurons, and their sub-cellular compartments in CTX and HIP cell layers (representative data in Fig. 5a). The ICHD-bearing vs. normal neuron dichotomy observed via X-PCI-CT (Fig. 5c) is also present in the XFM data. On one side, normal neurons show limited somatic involvement of the measured elements, and on the other side, neurons more densely filled with P, S, and especially Ca and Fe were observed. This was well visualized in the superimposed elemental distributions of co-localization XFM maps (Fig. 5b). Since histological processing has an impact on the retention of the measured elements, especially in the case of Ca and Fe [58, 59], the relevance of this quantification lies especially in its comparative value across the two identified cell groups (more in Suppl. Results).

Based on the average intensity of their phase maps, we classified all XFM-measured cells as either ICHD-bearing or normal, segmented them (Fig. 5d), and quantified the mean intracellular elemental content in the two cell groups (Fig. 5e). ICHD-bearing cells showed patterns of increased diffuse accumulation of P, S, and Ca deposition in cytoplasmic, nuclear, and even dendritic/axonal compartments, compared to normal cell somas, which instead presented milder cytoplasmic and dendritic/axonal levels of P, S, and Ca, and relatively little nuclear involvement. Interestingly, also diffuse Fe deposition was noticeable in somatic compartments of ICHD-bearing cells, whereas both ICHD-bearing and normal neuron XFM maps contained small globular deposits in near-nuclear regions, likely contained within nucleoli, cell organelles, or neurovesicles [60]. Trace quantities of K, Zn, Cu, and Br were also detected. Notably, the mean level of intracellular elemental content (2D density) was found to be significantly higher within ICHD cells (*n* = 11) than within normal non-ICHD-bearing cells (*n* = 22) for P, S, Ca, and Fe (Suppl. Figure 8d). Since increased metal content can be a sign of degenerative cellular processes (e.g., cell condensation, apoptosis/necrosis, dystrophic mineralization of cell bodies or ferrugination, hyper-phosphorylation of p-tau fibrils or Fe-trapping by Aβ oligomers), this observation reinforces an interpretation of the neuronal ICHD measured via X-PCI-CT as intracellular protein and metal accumulations due to either neuron aging or to AD-linked neurodegeneration respectively in WT and 3xTgAD animals (more in Suppl. Results).

The observed sensitivity of X-PCI-CT to ICHD was compared to that of a complementary morphological 3D neuroimaging technique, namely high-field 9.4-T MRI data with ~ 20^3^ µm^3^ voxel size. One 13-month-old 3xTgAD brain sample was scanned before (Suppl. Figure 10) and after postmortem application of a gadolinium-based MRI contrast agent (Suppl. Fig. 11a-b) and compared to 3.0^3^ µm^3^ voxel X-PCI-CT data (Suppl. Figure 11c). The higher spatial resolution of X-PCI-CT permitted a more precise rendition of the sub-cellular lesions. In CTX layers, sparse individual ICHD particles, visible via X-PCI-CT, were hardly recognizable as isolated hypodense voxels in MR images at similar locations (Suppl. Figure 11b). Instead, extended abnormal layer-like hypo-density regions were observed within key hippocampal layers (e.g., CAsp) by MRI, correlating with X-PCI-CT-based measurements of layer-like HD at similar locations (Suppl. Figure 11a-c). Given the known correlation between MRI hypo-intensity, Aβ, and Fe deposition [61], the good agreement we observed here between MRI hypo-intensity and X-PCI-CT ICHD signal further ties the biological origin of the ICHD measured in 3xTgAD mice to known AD-linked processes of cellular neurodegeneration, i.e. intracellular proteopathy with metal co-localization [62] (more in Suppl. Results).

We also compared X-PCI-CT to TEM (Suppl. Figure 11d-i), the gold-standard technology for morphological studies of sub-cellular structure that is sensitive (much like X-PCI-CT) to local differences in electron density (more in Suppl. Results). While TEM results feature higher (sub-nanometric) spatial resolving power compared to that of nano-X-PCI-CT, normal HIP CAsp neuron morphology visualized via X-PCI-CT nano-imaging correlated very well with TEM imaging of similar cell groups (Suppl. Figure 11d-f). In 3xTgAD mice, neurons bearing somato-dendritic ICHD presented signs of pathological cell morphology visible both via X-PCI-CT and via TEM (Suppl. Figure 11 g-i): Ill-shaped cell-membrane outlines and cytoplasmic blebbing disrupted nuclear lamina with tubular invaginations, and dense fibrillary lesions within dendritic and axonal compartments. These ultrastructural observations of ICHD-bearing neurons in 3xTgAD animals correlate with cellular aging or intracellular accumulation of protein deposits (e.g., aging [63]-induced or tauopathy [64]-induced nuclear membrane laminopathy), and with cell death (e.g., cytoplasmic blebbing is typical of cellular apoptosis [65]).

### Part III

#### X-PCI-CT 3D virtual histology to quantify AD-linked cellular neurodegeneration after systemic pharmacological treatment

After establishing (“Part II” section) X-PCI-CT-detected ICHD in aged rodent brains as a postmortem biomarker of intracellular aging and neurodegenerative processes, we applied it in a proof-of-principle study on AD neurodegeneration. X-PCI-CT was used (“Part III” section) as a density-based 3D virtual histology of deep neuronal cell populations for the quantification of brain-wide volumetric levels of cellular lesions. For 1 month, both aged WT and aged 3xTgAD mice were chronically treated with LY379268, known to induce neuroprotection in experimental models [33, 35]. The collected X-PCI-CT data could serve as the basis for a preliminary evaluation of this experimental pharmacological treatment.

First, to quantitatively measure cellular aging and neurodegeneration label-free, HD particles were extracted from 3.0^3^ to 0.1^3^ µm^3^ voxel X-PCI-CT 3D datasets of CTX and HIP layers via an automatic threshold-based segmentation algorithm (see the “Method summary” section). Annotations of X-PCI-CT-based MIP maps and segmentation masks (Suppl. Figure 12a-d, Suppl. Figure 13) made clear that extracted cell-sized HD particles could be divided, based on size, in three main cellular populations: ICHD-bearing neurons (the largest HD particles), hyperdense glial cells (particles of intermediate size), and nucleoli within hypodense normal neurons (the smallest HD particles). A small amount (< 10 objects per half brain sample) of larger extra-cellular HD clusters with senile plaque-like morphology (diameter ~ 100 µm, mostly of thalamic and brain-stem origins, Suppl. Figure 13) was also extracted by the auto-threshold algorithm. To focus on early intracellular AD pathology, though, the plaque-like HD (still relatively scarce in our 13-month-old 3xTgAD animals) was omitted from the main analysis. Finally, quantification of the data was achieved by application of 3D object-counting particle size-measuring algorithms to the segmented HD particle volumes. This approach, applied to 0.7^3^, 0.3^3^, and 0.1^3^ µm^3^ voxel size X-PCI-CT data, afforded tri-modal particle size distributions (Suppl. Figure 12e), in good agreement with a 3-population model for HD particles (ICHD-bearing neurons vs. glia vs. nucleoli of normal neurons). The 3.0^3^ µm^3^ voxel size X-PCI-CT data only produced bimodal distributions, since such an optical system is unable to spatially resolve HD nucleoli that are only a few microns in diameter.

**Fig. 6:**
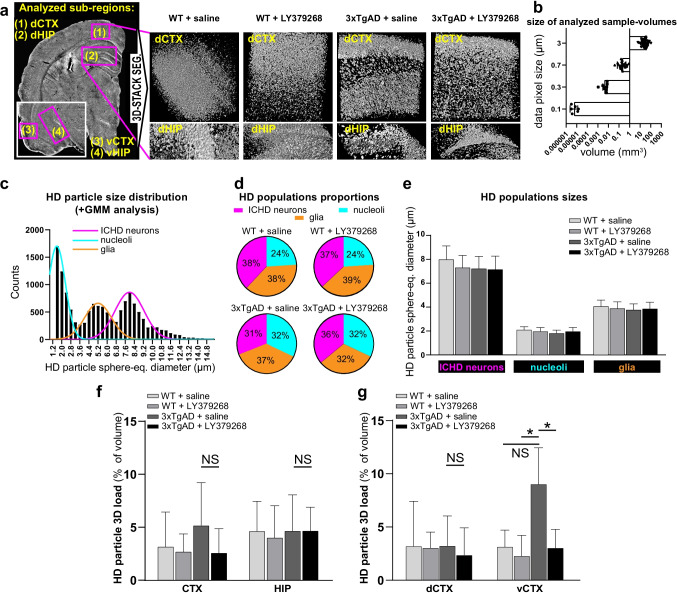
Proof-of-principle drug test with the group II metabotropic glutamate receptor agonist LY379268. **a** Experimental animal groups (13-month-old mice): WT treated with saline (*n* = 4), WT treated with LY379268 (*n* = 4), 3xTgAD treated with saline (*n* = 3), 3xTgAD treated with LY379268 (*n* = 4). Virtual-histological method: collection of 3.0^3^–0.1^3^ µm^3^ voxel multiscale X-PCI-CT, and detection, segmentation, and quantification of HD particles (as in Suppl. Figure 12), treated as markers of AD-linked cellular neurodegeneration. Analysis limited to 66 total sample volumes of interest (VOI) (representative 3D renderings in **a** within four brain regions: (1) dorsal CTX (dCTX), (2) dorsal HIP (dHIP), (3) ventral CTX (vCTX), and (4) ventral HIP (vHIP). **b** Sizes of analyzed brain tissue VOI at each imaging resolution. Data show volumetric size of individual VOI, and group mean ± SD. **c** Representative tri-modal HD particle size distribution, extracted from a 0.3^3^ µm^3^ voxel VOI, and Gaussian mixture model (GMM) fit, used to separate three HD particle populations based on size (ICHD-bearing vs. glia vs. normal neuron nucleoli). GMM analysis on all sample volume extracted **d** group HD population proportions (pie charts show group mean % of total HD particles) and **e** group mean particle size for each HD population (bar graph shows group mean HD particle size ± SD), group mean HD particle 3D load (bar graphs show group mean total HD particle volume % of total sample volume) in **f** CTX vs. HIP and **g** dCTX vs. vCTX sample volumes, for all four animal groups. No significant differences between animal groups were found in **d-f** and **g**, dCTX, whereas significant differences in 3D load between animal groups were found in **g** vCTX sample volumes. *P*-values in **d**-**g** calculated by one-way ANOVA testing with Turkey-Kramer’s multiple comparison post hoc test. *P < 0.05. NS, not significant

The drug test included multiscale X-PCI-CT brain data from all fifteen 13-month-old mice involved in the “Part II” and “Part III” sections of the study, seven WT and eight 3xTgAD, chronically treated with either saline or LY379268, and thus divided into four experimental groups (Fig. 6a). Quantifications were performed after the sampling of tissue volumes (66 VOI in total, see the “Method summary” section) within four AD-linked brain regions (dorsal and ventral CTX and HIP, Fig. 6a). Examined sample volumes ranged between 30 mm^3^ in size, using the largest-field-of-view 3.0 µm^3^ voxel data, and ~ 8000 µm^3^ in size, using the smallest-field-of-view 0.1 µm^3^ voxel data (Fig. 6b). Gaussian mixture model (GMM) analysis of the tri-modal particle size distributions (Fig. 6c), obtained as in Suppl. Figure 12 after HD particle segmentation and quantification within each sample volume (VOI), led to the demonstrative extraction of one population, one morphological, and one aging/neurodegeneration parameter, respectively proportions of HD particle populations, HD particle sizes, and 3D HD particle tissue load, all relevant to the study of aging and AD lesions.

The average equivalent-sphere diameter of ICHD-bearing neurons was quantified to almost 8 µm that of HD glial cells to almost 4 µm and that of nucleoli to almost 2 µm (Fig. 6e), with no significant difference between animal groups. Differences in HD population proportions were also found not to be significant (Fig. 6d). The quantification of HD particle load showed conspicuous load variability already between different sample volumes of the same animal (Suppl. Figure 14). More HD particle load was found, on average, within CTX layers of saline-treated 3xTgAD mice, but this difference was not significant (Suppl. Figure 14), even after limiting calculations to individual layers (CTX and HIP, Fig. 6f). After further restricting of the analysis to cortical areas only (Fig. 6g), the load within tissue samples from ventral CTX regions in untreated 3xTgAD mice was found to be significantly higher than that within tissue samples from ventral CTX regions in 3xTgAD mice and WT mice treated with LY379268 (more in Suppl. Results). In good agreement with previous observations on neuronal cultures [66], these proof-of-principle findings suggested possible mild LY379268-ligand-driven neuroprotective effects with sub-regional efficacy on the aging AD rodent brain with respect to untreated 3xTgAD mice and call for a larger-scale study.

## Discussion

We developed a mesoscale (organ-scale to cellular-scale) probing system for volumetric postmortem morphological neuroimaging based on state-of-the-art, multiscale synchrotron-based X-PCI-CT. X-PCI-CT image contrast is generated via a label-free mechanism and quantitatively describes intra-sample X-ray phase variations proportional to local electron density. Their freedom from labels and stains qualifies these images as anatomically dense and unbiased direct measurements of nervous-tissue structure. This 3D imaging tool was applied to the brain-wide study of cellular hyperdensity in aged WT and aged 3xTgAD mice, an experimental AD mouse model. It was shown that X-PCI-CT provides micro-resolution to nano-resolution 3D neuroanatomical representations of deep neuronal and glial populations within extended rodent brain tissue samples after little manipulation beyond standard sample fixation and paraffin embedding, with invasive sample sectioning necessary only for the 0.1^3^ voxel images. Therefore, high-resolution X-PCI-CT maps provide a means to virtually visualize 3D brain histology with electron density-based coloring.

The collected X-PCI-CT maps, in conjunction with morphological observations, enabled discrimination between different neuron types and different glial populations. Moreover, the X-PCI-CT technique was sensitive to small intra-neuronal density differences, enabling the detection of hyperdense neurons (ICHD) in brain tissues from both aged WT and aged 3xTgAD. The X-PCI-CT-based brain density maps located ICHD in somatic, nuclear, and dendritic/axonal cellular compartments of neurons in key AD-linked cell layers. The observation of ICHD was interpreted as abnormal intra-neuronal protein accumulation or abnormal lysosomal deposits likely arising due to cell aging-related processes (in WT mice) or neurodegeneration-related cellular processes (in 3xTgAD mice). This imaging method could thus be established as a tool for full-organ mapping of aging-associated and AD-associated intracellular lesions.

Since X-PCI-CT signal is biologically nonspecific, ICHD-bearing cells could represent a mixture of hyperdense normally aging or degenerated cell types. A biological characterization of the ICHD particles in 3xTgAD mice was achieved here via extensive multi-technique comparative analysis, involving fluorescence histology, IHC, XFM, MRI, and TEM. All results confirmed that the ICHD signal in 3xTgAD mice is highly likely representative of age-related AD-associated neurodegenerative processes of co-localized Aβ and p-tau intracellular deposition. Notably, both tissue-level and cellular-level patterns of ICHD positivity (Fig. 3) matched cellular markings in collected ThioS-dyed sections and patterns of amyloid and tau immunoreactivity in collected IHC sections (Figs. 1, 4). Somatic compartmentalization of Aβ and dendritic/axonal localization of p-tau fluorescent signal in IHC section suggests a spatially differentiated multi-peptidic agglomeration of AD-linked proteins in ICHD-bearing cells. Aged WT mice also presented ICHD (Fig. 2) and some level of ThioS fluorescence (Fig. 1). The very low levels of immunoreactivity for Aβ and p-tau in the WT mice (Suppl. Figure 9) suggest the ICHD observed in WT animals be likely related to normal neuron aging processes (e.g., lipofuscin granula formation).

Compared to high-field MRI (Suppl. Figures 10–11), which achieves pre-cellular spatial resolution [67] after tens of hours of scanning time in the absence of contrast agent, X-PCI-CT with brilliant synchrotron-radiation X-rays presents clear advantages in terms of spatial resolution (sub-cellular) and measurement durations (minutes to a few hours [37], label-free). The subtlest sub-cellular lesions and protein deposits typical of early AD phases, such as axonal p-tau NFT lesions, are, in fact, especially elusive to high-field MRI [68]. Compared to TEM, in practice a 2D imaging method for ultrathin sections, X-PCI-CT can capture 3D cellular neuroanatomy within extended un-sliced brain samples.

What stands out in the collected XFM data is the observed perisomatic near-nuclear Fe and Ca increased concentrations in ICHD cells compared to normal cells (Fig. 5). While the nuclear Fe aggregates in normal cells are consistent with anti-fibrillarin IHC nuclear staining of normal neurons [69], diffuse somatic hyper-accumulation of Ca and Fe metals within ICHD-labeled neurons represents a sign of likely cellular dysfunction, arising either in direct connection to toxic protein clumping (e.g., by protein chelation), or due to cell aging and cell death-related processes. Disturbances in iron metabolism have been coupled to several neurodegenerative diseases [70] and, indeed, iron, either directly bound to amyloid and tau lesions [71] or associated to cytoplasmic RNA [72], is known to play a toxic role in AD pathology [62], and cause oxidative damage and neurodegeneration. Aβ aggregates, in turn, can induce Ca dyshomeostasis [73] and lead to cellular synaptic dysfunction, neurodegeneration [74], and cell death (apoptosis [75], necrosis, or autophagy [76]). Overall, these multi-technique comparative analyses established a clear correlation between the ICHD signal, detected via X-PCI-CT, and multiple concurrent and possibly co-localizing intracellular processes related to aging in WT mice, and to amyloid-driven and tau-driven AD-linked cellular proteopathy and neurodegeneration in 3xTgAD mice.

The attained characterization of the ICHD signal as a nonspecific cellular biomarker of neuronal aging and degeneration allowed the application of multiscale X-PCI-CT for the postmortem brain-wide 3D detection and quantification of cellular hyperdensity within small animal brain samples, establishing a novel platform for quantitative cell-by-cell screening and quantification. As a proof-of-concept, we studied the response of aged WT and 3xTgAD mice to a systemic chronic treatment with LY379268, a potential neuroprotective drug. LY379268 activates mGlu2 and mGlu3 metabotropic glutamate receptors and, thus, may reduce excitatory synaptic transmission, and consequently excitotoxicity by reducing glutamate release and increasing glutamate clearance [34, 77, 78]. Moreover, mGlu3 receptors may induce neuroprotective effects by increasing the production of neurotrophic factors and enhancing glutamate clearance [33]. Moreover, chronic treatment with LY379268, leading to an increased production of glial-derived neurotrophic factor, does not lead to the development of tolerance, making this treatment optimal [79]. The analysis of X-PCI-CT data (Fig. 6) pointed out regional differences in ICHD and, most notably, significant lower levels of agglomerates in ventral CTX layers of 3xTgAD mice treated with the drug. Our estimates of lesion tissue load, in the 1–5% range, are in good agreement with a similar X-PCI-based AD-linked protein-deposit quantification performed on the 5xFAD genetic animal model, which reported neocortical amyloid load levels of around 2% in similarly aged animals [25]. Overall, these results are encouraging and provide a rationale for a future larger-scale study.

In summary, the multiscale characterization of intracellular hyperdensity, obtained by 3D micro-to-nano-imaging of deep neuronal populations vulnerable to proteopathy in key brain layers, cross-validated by other imaging modalities, represents a novel methodological advance of this study. The intracellular hyperdensity in cortical and hippocampal neurons of 3xTgAD mice was characterized as hyper-accumulation of Aβ and p-tau proteins, with the involvement of several key bio-elements, including calcium and iron. Furthermore, the obtained structural-morphological discrimination of glial vs. neuronal cells demonstrated that a precise focusing of the neuropathological evaluation is also possible via this methodology. The viability of this approach for an evaluation of experimental neuroprotective strategies was demonstrated by the efficient unbiased label-free screening of full-organ rodent brains achieved in the “Part III” section of this study.

The X-PCI-CT virtual-histological approach presents the evident benefit over traditional histology and IHC that full-organ structural analyses without regional bias can be performed in a sample-preserving fashion. State-of-the-art IHC approaches to study brain neuroanatomy, in fact, permit rather cumbersome 3D cellular imaging, but are limited in terms of organ coverage, involve sample sectioning and complicated reconstructions with stitching and aligning issues, and require the use of various intrinsically biased labels (histologic stains, immuno-labels, contrast agents). Recently emerging super-resolution 3D neuroimaging technologies, mainly based on tissue clearing [80] and expansion [81] or two-photon microscopy [82], enable brain-wide cellular resolution structural and functional investigations of entire cell populations [83], and down to single cells [84] and single intracellular molecules [85]. These methods can be used for organ-level transcriptomics and connectomics, and to investigate complex biological processes such as aging and neurodegeneration [86]. Still, they are based on fluorescence light microscopy, thereby falling short of delivering completely unbiased and anatomically dense visualizations of neural tissue [87], due to the notorious issue of sparse labeling [88] by means of antibodies or small molecule tags. Super-resolution electron microscopy (EM) techniques, such as transmission EM (TEM), serial block-face scanning EM (SBEM), or focused ion beam scanning EM (FIB-SEM), are based on label-free cyto-architecture-detection mechanisms and therefore enable anatomically dense characterization of both intra-neuronal amyloid and tau pathology at synaptic resolution by using heavy metal staining. However, these techniques rely on ultrathin sectioning and ablation, generate very large data sets [89] requiring alignment and stitching, have to overcome the hurdle of long acquisition times [90], and are limited to very small tissue volumes, far from rodent 3D whole-brain throughput capabilities.

The X-PCI-CT method, instead, affords 3D-morphological measurements comparable to what light microscopy and TEM approaches can provide on 2D thin sections. An evident benefit of a dissection-free multiscale imaging methodology is its ability to collect pre-cellular to cellular-level maps at variable spatial resolutions and from different deep brain regions, simply by running an appropriately aimed sequence of less invasive local CT scans, without having to go through a series of error-prone mechanical sample slicing operations. Furthermore, while one-shot histological workup requires strategic pre-planning before data collection, X-PCI-CT allows for repeated sample interrogations, admits unexpected observations, and can provide a morphological database on which to plan further data collection. Sample-preserving X-PCI-CT imaging in fact is conveniently compatible with most other postmortem neuroimaging analyses. Both the XFM and TEM analyses presented in our work, for example, were carried out on the same brain samples previously imaging via X-PCI-CT. In addition, though the histological and IHC analyses were performed on contralateral hemispheres for practical and time sparing reasons contingent to this multi-institutional study, X-PCI-CT is also compatible with post-imaging same-sample histological and IHC work [12, 29]. The only postmortem imaging technique that needs to precede X-PCI-CT is MRI, for which a fully hydrated sample is necessary.

The high radiation doses as well as the scan times, used in high-resolution (micro/nano-scale) X-PCI-CT in this work, are not compatible with the imaging of living humans. This imaging approach, though, can be readily applied to human tissues postmortem, e.g. by using autoptic brain samples, e.g. from hippocampus and cerebral cortex tissues. Bioptic material could also be studied if collectable, especially in other organ systems. This methodology could e.g. be used in postmortem human brain samples of people affected by Alzheimer’s disease who had been treated with drugs to reduce the neurodegeneration. This would allow quantifying the potential neuroprotective effect of drugs administered at earlier times in patients showing precocious symptoms of disease or mild cognitive impairment. Moreover, this approach could also be used e.g. in postmortem human brain samples of brain cancer patients to confirm and quantify the antineoplastic effect of anticancer drug treatments. As in the small animal case, human brain tissues should be analyzed after their extraction from the bony skull to avoid the formation of scattering artifacts and disturbances of the X-ray wave front (needed for optimal image quality). Sample size limitations become more important as the spatial resolution increases, just as in the case of the small animal samples used in this work. Tailored multiple local-tomography scan protocols may enable full human brain sample coverage and micrometric (3.0^3^ to 0.7^3^ micron voxel) resolution in the future. Dataset sizes may become the limiting factor in this case. The nano-holotomography setup instead currently limits the sample size to few cubic millimeters.

Summarizing, the multiscale X-PCI-CT approach adds the following information to a cellular-level analysis of an AD mouse brain sample: a volumetric mapping and quantification of sub-cellular-level neurodegenerative lesions in all brain regions (full-organ imaging), an unbiased load calculation, and a quantitative characterization of the sizes and the shapes of these lesions within a single tissue-preserving examination. A more in-depth discussion of the main themes of this study is included as supplementary material (see Suppl. Discussion).

## Conclusions

The complexity of biological systems, such as the brain, and of biological processes, such as aging and neurodegeneration, requires a multi-perspective and multi-technique approach to data acquisition. Multiscale X-PCI-CT complements established neuroimaging approaches in the quest for a better understanding of the intricate bio-mechanisms of the nervous system. The results presented here demonstrate that high-resolution X-PCI-CT imaging allows multiscale 3D morphological analyses on extended deep label-free neuronal populations. This method enables brain-wide quantitative detection of cellular aging and neuropathology associated with brain disorders and offers a versatile tool for unbiased in-depth evaluations of experimental neuroprotective strategies.

## Supplementary Information

Below is the link to the electronic supplementary material.

Supplementary file1 (PDF 7.86 MB)

Supplementary file2 (MP4 105750 KB)

Supplementary file3 (MP4 105750 KB)

Supplementary file4 (MP4 105750 KB)

Supplementary file5 (MP4 105750 KB)

Supplementary file6 (MP4 105750 KB)

Supplementary file7 (MP4 105750 KB)

Supplementary file8 (MP4 105750 KB)

Supplementary file9 (MP4 105750 KB)

## Data Availability

The original CT raw data collected in this research project are archived at the European Synchrotron (ESRF) and can all be accessed upon request of the corresponding author. Prior to publication, it is our intention to make this data openly available via the ESRF data catalog (data.esrf.fr) or directly via the ESRF DOI Portal (doi.esrf.fr). All processed data files used in the submitted article figures will also be included in the file repository.

## References

[1] Musiek ES, Holtzman DM (2015). Three dimensions of the amyloid hypothesis : time, space and ‘wingmen’. Nat Neurosci.

[2] Selkoe DJ. Resolving controversies on the path to Alzheimer’s therapeutics. Nat Med 2011;17 10.1038/nm.2460 21900936

[3] Mattson MP, Chan SL, Duan W (2002). Modification of brain aging and neurodegenerative disorders by genes, diet, and behavior. Physiol Rev.

[4] Braak H, Braak E (1991). Neuropathological staging of Alzheimer-related changes. Acta Neuropathol.

[5] Nordberg A, Rinne JO, Kadir A, Långström B (2010). The use of PET in Alzheimer disease. Nat Rev Neurol.

[6] Fitzgerald R. Phase-sensitive X-ray imaging. Phys. Today 2000;**7**

[7] Beltran MA (2011). Interface-specific x-ray phase retrieval tomography of complex biological organs. Phys Med Biol.

[8] Bravin A, Coan P, Suortti P (2013). X-ray phase-contrast imaging: from pre-clinical applications towards clinics. Phys Med Biol.

[9] Cloetens P, Barrett R, Baruchel J, Guigay J-P, Schlenker M (1996). Phase objects in synchrotron radiation hard x-ray imaging. J Phys D Appl Phys.

[10] Snigirev A, Snigireva I, Kohn V, Kuznetsov S, Schelokov I (1995). On the possibilities of x-ray phase contrast microimaging by coherent high-energy synchrotron radiation. Rev Sci Instrum.

[11] Pfeiffer F (2007). High-resolution brain tumor visualization using three-dimensional x-ray phase contrast tomography. Phys Med Biol.

[12] Barbone GE (2018). Micro-imaging of brain cancer radiation therapy using phase-contrast computed tomography. Int J Radiat Oncol Biol Phys.

[13] Khimchenko A (2018). Hard X-ray nanoholotomography: large-scale, label-free, 3D neuroimaging beyond optical limit. Adv Sci.

[14] Mittone A (2017). Characterization of a sCMOS-based high-resolution imaging system. J Synchrotron Radiat.

[15] Mader K (2011). High-throughput full-automatic synchrotron-based tomographic microscopy. J Synchrotron Radiat.

[16] Mokso R, Cloetens P, Maire E, Ludwig W, Buffiere J-Y (2007). Nanoscale zoom tomography with hard x rays using Kirkpatrick-Baez optics. Appl Phys Lett.

[17] Da Silva JC (2017). Efficient concentration of high-energy x-rays for diffraction-limited imaging resolution. Optica.

[18] Cedola A (2017). X-ray phase contrast tomography reveals early vascular alterations and neuronal loss in a multiple sclerosis model. Sci Rep.

[19] Lathuilière A (2016). A subcutaneous cellular implant for passive immunization against amyloid-β reduces brain amyloid and tau pathologies. Brain.

[20] Töpperwien M, van der Meer F, Stadelmann C, Salditt T (2018). Three-dimensional virtual histology of human cerebellum by X-ray phase-contrast tomography. Proc Natl Acad Sci.

[21] Töpperwien M. et al. Three-dimensional mouse brain cytoarchitecture revealed by laboratory-based x-ray phase-contrast tomography. Sci Rep 2017;**7** 10.1038/srep42847 PMC5327439 28240235

[22] Dyer EL. et al. Quantifying mesoscale neuroanatomy using X-ray microtomography. eNeuro 2017;4(5) 10.1523/ENEURO.0195-17.2017 PMC5659258 29085899

[23] Noda-Saita K (2006). Quantitative analysis of amyloid plaques in a mouse model of Alzheimer’s disease by phase-contrast X-ray computed tomography. Neuroscience.

[24] Connor DM (2009). Computed tomography of amyloid plaques in a mouse model of Alzheimer’s disease using diffraction enhanced imaging. Neuroimage.

[25] Pinzer BR (2012). Imaging brain amyloid deposition using grating-based differential phase contrast tomography. Neuroimage.

[26] Astolfo A, Lathuiliere A, Laversenne V, Schneider B, Stampanoni M (2016). Amyloid-beta plaque deposition measured using propagation-based X-ray phase contrast CT imaging. J Synchrotron Radiat.

[27] Massimi L (2019). Exploring Alzheimer’s disease mouse brain through X-ray phase contrast tomography: From the cell to the organ. Neuroimage.

[28] Okamura N (2016). Advances in the development of tau PET radiotracers and their clinical applications. Aging Res Rev.

[29] Töpperwien M, van der Meer F, Stadelmann C, Salditt T (2020). Correlative x-ray phase-contrast tomography and histology of human brain tissue affected by Alzheimer’s disease. Neuroimage.

[30] Oddo S (2003). Triple-transgenic model of Alzheimer’s disease with plaques and tangles : intracellular Aβ and synaptic dysfunction. Neuron.

[31] Oddo S, Caccamo A, Kitazawa M, Tseng BP, Laferla FM (2003). Amyloid deposition precedes tangle formation in a triple transgenic model of Alzheimer’s disease. Neurobiol Aging.

[32] Nicoletti F (2011). Metabotropic glutamate receptors: from the workbench to the bedside. Neuropharmacology.

[33] Bruno V (2017). The impact of metabotropic glutamate receptors into active neurodegenerative processes: a “dark side” in the development of new symptomatic treatments for neurologic and psychiatric disorders. Neuropharmacology.

[34] Aronica E (2003). Expression and functional role of mGluR3 and mGluR5 in human astrocytes and glioma cells: opposite regulation of glutamate transporter proteins. Eur J Neurosci.

[35] Bruno V (2001). Metabotropic glutamate receptor subtypes as targets for neuroprotective drugs. J Cereb Blood Flow Metab.

[36] Stampanoni M (2007). TOMCAT: a beamline for tomographic microscopy and coherent radiology experiments. AIP Conf Proc.

[37] Pacureanu A, da Silva JC, Yang Y, Bohic S, Cloetens P. Nanoscale three-dimensional imaging of biological tissue with x-ray holographic tomography. in Proceedings of the SPIE 2018;10711, 107112B

[38] Paganin D, Mayo SC, Gureyev TE, Miller PR, Wilkins SW (2002). Simultaneous phase and amplitude extraction from a single defocused image of a homogeneous object. J Microsc.

[39] Bartels M, Krenkel M, Cloetens P, Möbius W, Salditt T (2015). Myelinated mouse nerves studied by X-ray phase contrast zoom tomography. J Struct Biol.

[40] Cloetens P (1999). Holotomography: quantitative phase tomography with micrometer resolution using hard synchrotron radiation x rays. Appl Phys Lett.

[41] Zabler S, Cloetens P, Guigay J-P, Baruchel J, Schlenker M (2005). Optimization of phase contrast imaging using hard x rays. Rev Sci Instrum.

[42] Lyckegaard A, Johnson G, Tafforeau P (2011). Correction of ring artifacts in X-ray tomographic images. Int J Tomogr Stat.

[43] Schneider CA, Rasband WS, Eliceiri KW (2012). NIH image to ImageJ: 25 years of image analysis. Nat Methods.

[44] Kapur JN, Sahoo PK, Wong AKC (1985). A new method for gray-level picture thresholding using the entropy of the histogram. Comput Vision Graph Image Process..

[45] Bolte S, Cordelières FP (2006). A guided tour into subcellular colocalization analysis in light microscopy. J Microsc.

[46] Sanchez-Cano C (2017). Synchrotron X-ray fluorescence nanoprobe reveals target sites for organo-osmium complex in human ovarian cancer cells. Chemistry.

[47] De Samber B (2018). Nanoscopic X-ray fluorescence imaging and quantification of intracellular key-elements in cryofrozen Friedreich’s ataxia fibroblasts. PLoS ONE.

[48] Solé VA, Papillon E, Cotte M, Walter P, Susini J (2007). A multiplatform code for the analysis of energy-dispersive X-ray fluorescence spectra. Spectrochim Acta Part B At Spectrosc.

[49] Ullmann JFP (2012). Segmentation of the C57BL/6J mouse cerebellum in magnetic resonance images. Neuroimage.

[50] Vallet PG (1992). A comparative study of histological and immunohistochemical methods for neurofibrillary tangles and senile plaques in Alzheimer’s disease. Acta Neuropathol.

[51] Honson NS (2007). Differentiating Alzheimer disease-associated aggregates with small molecules. Neurobiol Dis.

[52] Rauch JN, Olson SH, Gestwicki JE (2017). Interactions between microtubule-associated protein tau (MAPT) and small molecules. Cold Spring Harb Perspect Med.

[53] Cammalleri M, Bagnoli P, Bigiani A (2019). Molecular and cellular mechanisms underlying somatostatin-based signaling in two model neural networks, the retina and the hippocampus. Int J Mol Sci.

[54] Yankner BA, Lu T, Loerch P (2008). The aging brain. Annu Rev Pathol Mech Dis.

[55] Landfield PW, Baskin RK, Pitler TA (1981). Brain aging correlates: retardation by hormonal-pharmacological treatments. Science.

[56] Landfield PW, Braun LD, Pitler TA, Lindsey JD, Lynch G (1981). Hippocampal aging in rats: a morphometric study of multiple variables in semithin sections. Neurobiol Aging.

[57] Gramaccioni C (2020). Cryo-nanoimaging of single human macrophage cells: 3D structural and chemical quantification. Anal Chem.

[58] Wróbel PM (2020). Feasibility study of elemental analysis of large population of formalin fixed paraffin embedded tissue samples – preliminary results. Spectrochim Acta Part B At Spectrosc.

[59] Morris CM, Candy JM, Oakley AE, Bloxham CA, Edwardson JA (1992). Histochemical distribution of non-haem iron in the human brain. Acta Anat (Basel).

[60] Ortega R, Cloetens P, Devès G, Carmona A, Bohic S (2007). Iron storage within dopamine neurovesicles revealed by chemical nano-imaging. PLoS ONE.

[61] Meadowcroft MD, Connor JR, Smith MB, Yang QX (2009). MRI and histological analysis of beta-amyloid plaques in both human Alzheimer’s disease and APP/PS1 transgenic mice. J Magn Reson Imaging.

[62] Honda K, Casadeus G, Petersen RB, Perry G, Smith MA (2004). Oxidative stress and redox-active iron in Alzheimer’s disease. Ann N Y Acad Sci.

[63] Honavar M, Lantos PL (1987). Ultrastructural changes in the frontal cortex and hippocampus in the aging marmoset. Mech Aging Dev.

[64] Frost B, Bardai FH, Feany MB (2016). Lamin dysfunction mediates neurodegeneration in tauopathies. Curr Biol.

[65] Wang Y (2017). Myosin IIA-related actomyosin contractility mediates oxidative stress-induced neuronal apoptosis. Front Mol Neurosci.

[66] Caraci F (2011). Targeting group II metabotropic glutamate (mGlu) receptors for the treatment of psychosis associated with Alzheimer’s disease: selective activation of mGlu2 receptors amplifies β-amyloid toxicity in cultured neurons, whereas dual activation of mGlu2. Mol Pharmacol.

[67] Wei H (2016). Imaging whole-brain cytoarchitecture of mouse with MRI-based quantitative susceptibility mapping. Neuroimage.

[68] Johnson GA (2007). High-throughput morphologic phenotyping of the mouse brain with magnetic resonance histology. Neuroimage.

[69] Farley KI, Surovtseva Y, Merkel J, Baserga SJ (2015). Determinants of mammalian nucleolar architecture. Chromosoma.

[70] Sfera A, Bullock K, Price A, Inderias L, Osorio C (2018). Ferrosenescence: the iron age of neurodegeneration?. Mech Aging Dev.

[71] Smith MA, Harris PLR, Sayre LM, Perry G (1997). Iron accumulation in Alzheimer disease is a source of redox-generated free radicals. Proc Natl Acad Sci.

[72] Nunomura A (1999). RNA oxidation is a prominent feature of vulnerable neurons in Alzheimer’s disease. J Neurosci.

[73] Kuchibhotla KV (2008). Aβ plaques lead to aberrant regulation of calcium homeostasis in vivo resulting in structural and functional disruption of neuronal networks. Neuron.

[74] Mattson MP (2007). Calcium and neurodegeneration. Aging Cell.

[75] Loo DT (1993). Apoptosis is induced by beta-amyloid in cultured central nervous system neurons. Proc Natl Acad Sci.

[76] Pinton P, Romagnoli A, Rizzuto R, Giorgi C. Ca2+ signaling, mitochondria and cell death. Curr Mol Med 2008;8 10.2174/156652408783769571 18336292

[77] Gegelashvili G, Dehnes Y, Danbolt NC, Schousboe A (2000). The high-affinity glutamate transporters GLT1, GLAST, and EAAT4 are regulated via different signalling mechanisms. Neurochem Int.

[78] Yao H-H (2005). Enhancement of glutamate uptake mediates the neuroprotection exerted by activating group II or III metabotropic glutamate receptors on astrocytes. J Neurochem.

[79] Battaglia G (2015). Activation of mGlu3 metabotropic glutamate receptors enhances GDNF and GLT-1 formation in the spinal cord and rescues motor neurons in the SOD-1 mouse model of amyotrophic lateral sclerosis. Neurobiol Dis.

[80] Pan C (2016). Shrinkage-mediated imaging of entire organs and organisms using uDISCO. Nat Methods.

[81] Murakami TC (2018). A three-dimensional single-cell-resolution whole-brain atlas using CUBIC-X expansion microscopy and tissue clearing. Nat Neurosci.

[82] Economo MN (2016). A platform for brain-wide imaging and reconstruction of individual neurons. Elife.

[83] Kim Y (2017). Brain-wide maps reveal stereotyped cell-type-based cortical architecture and subcortical sexual dimorphism. Cell.

[84] Moffitt JR (2018). Molecular, spatial, and functional single-cell profiling of the hypothalamic preoptic region. Science.

[85] Bon P. et al. Self-interference 3D super-resolution microscopy for deep tissue investigations. Nat Methods 2018;15 10.1038/s41592-018-0005-3 29713082

[86] Masuda T (2019). Spatial and temporal heterogeneity of mouse and human microglia at single-cell resolution. Nature.

[87] Venkataramani V (2018). Enhanced labeling density and whole-cell 3D dSTORM imaging by repetitive labeling of target proteins. Sci Rep.

[88] Lichtman JW, Denk W (2011). The big and the small: challenges of imaging the brain’s circuits. Science.

[89] Mikula S, Denk W (2015). High-resolution whole-brain staining for electron microscopic circuit reconstruction. Nat Methods.

[90] Eberle AL (2015). High-resolution, high-throughput imaging with a multibeam scanning electron microscope. J Microsc.

