# Supplementary material for: X-ray multiscale 3D neuroimaging to quantify cellular aging and neurodegeneration postmortem in a model of Alzheimer’s disease
Source: Eur J Nucl Med Mol Imaging. 2022 Jul 19;49(13):4338–57. doi: 10.1007/s00259-022-05896-5 (PMC9606093; doi:10.1007/s00259-022-05896-5)
Supplement: Supplementary file 1 — Supplementary file1 (PDF 7.86 MB) [file 259_2022_5896_MOESM1_ESM.pdf]

Supplementary Information for

X-ray multiscale 3D neuroimaging to quantify cellular aging and neurodegeneration postmortem in a model of Alzheimer's disease

AUTHORS:
Giacomo E. Barbone (1,2), PhD, Dr., giacomo.barbone@physik.uni-muenchen.de
Alberto Bravin (3,$), PhD, Prof. Dr., alberto.bravin@unimib.it
Alberto Mittone (3,#), PhD, Dr., amittone@cells.es
Alexandra Pacureanu (3), PhD, Dr., joitapac@esrf.fr
Giada Mascio (4)**,** MSc., Ms., giada.mascio@neuromed.it
Paola Di Pietro (4,%), MSc., Ms., pdipietro@unisa.it
Markus J. Kraiger (5), PhD, Dr., markus.kraiger@helmholtz-muenchen.de
Marina Eckermann (1,&), MSc., Ms., marina.eckermann@phys.uni-goettingen.de
Mariele Romano (1), PhD, Dr., mariele.romano@physik.uni-muenchen.de
Martin Hrabě de Angelis (5,6,7), PhD, Prof. Dr., hrabe@helmholtz-muenchen.de
Peter Cloetens (3), PhD, Dr., cloetens@esrf.fr
Valeria Bruno (4,8), MD, PhD, Prof. Dr., valeria.bruno@uniroma1.it
***Giuseppe Battaglia** (4,8), MD, PhD, Prof. Dr., giuseppe.battaglia@uniroma1.it
***Paola Coan** (1,2) PhD, Prof. Dr., paola.coan@physik.uni-muenchen.de

***Shared Corresponding Authors/Last Authors:**
***Prof. Dr. Giuseppe Battaglia**giuseppe.battaglia@uniroma1.it; +390865915211
Department of Physiology and Pharmacology, University Sapienza
Piazzale Aldo Moro 5; 00185 Roma, Italy
***Prof. Dr. Paola Coan**
paola.coan@physik.uni-muenchen.de; +49(0)89 289-14062
Department of Medical Physics, Faculty of Physics, Ludwig-Maximilians-Universität München
Am Coulombwall 1, 85748 Garching, Germany

This PDF file includes:

Supplementary text
Suppl. Figures 1 to 14
Legends for Videos 1 to 8
SI References

Other supplementary materials for this manuscript include the following:

Videos 1 to 8

Supplementary Information Text

SUPPLEMENTARY INTRODUCTION:

More on protein aggregation in neurodegeneration:

Studies indicate that the formation of lipofuscin granula can be due to the oxidative alteration of lipid and protein macromolecules[1], leading to the formation of abnormal intracellular aggregates.

More on X-PCI-CT:

Extra-cellular amyloid plaques lead to local X-PCI-CT signal hyper-intensity, which could be correlated to Thiflavin-S fluorescence[2], to hyperdensity signal in transmission electron microscopy (TEM)[3], and to IHC for Aβ[4] and tau[5]. Cell-body mineralization or lipofuscin granula formation, may also concurrently contribute to the formation of the abnormal signal hyper-intensity consistently observed in X-PCI-CT data.

SUPPLEMENTARY METHODS:

Acronyms: X-ray phase-contrast computed tomography (X-PCI-CT); cortex (CTX); hippocampus (HIP); hyperdensity (HD); intracellular hyperdensity (ICHD); X-ray fluorescence microscopy (XFM); magnetic resonance imaging (MRI); transmission electron microscopy (TEM).

Ethical compliance: Experimental procedures on animals were performed following the Guidelines for Animal Care and Use of the National Institutes of Health (Authorization number: 93/2011-B), were approved by the local institute Ethical Committee and the Italian Ministry of Health and carried out according to the Italian (D.L. 26/2014) and European Union Directive

(2010/63/EU) on the protection of animals used for scientific purposes. All efforts were made to minimize the number of animals used and to alleviate their discomfort.

Materials: LY379268, i.e. (-)-2-oxa-4-aminocyclo[3.1.0]hexane-4,6-dicarboxylic acid, was purchased from Tocris Cookson Ltd (Bristol, U.K). All other chemicals were purchased from Sigma (Milano, Italy).

Animals:

B6/129 wild-type control mice (Charles-River, Calco, Italy) and transgenic 3xTgAD mice (B6;129-Tg(APPSwe,tauP301L)1Lfa *Psen1*^{tm1Mpm}/Mmjax, Stock No: 34830-JAX) were purchased from the Jackson Laboratories (Bar Harbour, ME). All mice were housed in a controlled-temperature room (21-23 °C, humidity 40-50%) and maintained on a 12-h light/dark cycle with food and water *ad libitum*. Eight symptomatic 11-month old male B6/129 wild-type (WT) mice and seven male 3xTgAD mutant mice (for a total of 15 mice) were treated either with saline or the selective agonist of group II metabotropic glutamate receptors LY379268 by means of subcutaneously implanted osmotic mini-pumps (Alzet, Cupertino, CA), which deliver the drug continuously at the rate of 1 mg/kg/day for 28 days, starting at the symptomatic phase. The nominal delivery of the pump was 250 nl/h, corresponding to a cumulative dose of 1 mg/kg/day of LY379268. Control mice received an equivalent volume of saline. One month after treatment, at the age of 13 months, all mice were sacrificed, brains were dissected out, fixed in Carnoy solution overnight, and finally embedded in paraffin blocks. We used one brain hemisphere for MRI, X-PCI-CT, XFM and TEM, the other hemisphere for histology and immunohistochemistry. In addition to the 13 month old WT and 3xTgAD mice, one asymptomatic 2 month old WT mouse was purchased from

Charles-River Laboratories (Les Oncins, France), and included to the study as young-animal control in the preliminary imaging vs. Thioflavin S histological study. This animal was not subject to any drug treatment before its sacrifice at 120 days (~ 4 months) of age (same brain extraction, fixation and embedding procedures as for the aged animals).

Experimental treatment groups:

The aged WT and 3xTgAD mice were treated with saline or LY379268 (1 mg/kg) to obtain four experimental groups: 1. WT mice treated with saline (n = 4), 2. WT mice treated with LY379268 (n = 4), 3. 3xTgAD mice treated with saline (n = 3), 4. 3xTgAD mice treated with LY379268 (n=4).

Synchrotron X-PCI-CT acquisitions:

3D postmortem multiscale X-PCI-CT was performed (on the collected mouse half brain samples) using several imaging setups available at beamlines of synchrotron-radiation facilities. There, CT scans were performed by rotation of the sample in front of an either quasi-parallel or conic X-ray beam and by continuous acquisition of CT projection images at different angles. For micrometric scans with 3.0^3 to 0.3^3 μm^3 effective voxel size, no sectioning of tissue was necessary, and imaging was performed within the intact mouse half brains. 3.0^3 μm^3 voxel full-organ CT scans were performed with the samples kept within paraffin blocks. For 0.7^3 and 0.3^3 μm^3 voxel micro-CT imaging, most of the paraffin embedding was removed from samples via heating (using hot water heated to ~50 °C), and brain samples were de-embedded from paraffin blocks and put in a PBS and ethanol bath within sealed cylindrical Eppendorf plastic tubes. Nanometric 0.1^3 μm^3 voxel scans were performed in air within a vacuum chamber on ~ 2 x 2 x 4 mm^3-volume tissue biopsies manually excised and containing dorsal CTX and HIP areas.

X-PCI-CT data collection: one half brain X-PCI-CT dataset per animal was measured with the 3.0^3 μm^3 voxel setup (for a total of 15 half-organ 3D datasets). In addition, local tomographic scans aiming CTX and HIP layers were performed with the 0.7^3, 0.3^3 and 0.1^3 μm^3 voxel setups, respectively on 7, 4 and 3 animals. The limited beam-time available at high-resolution synchrotron X-PCI-CT imaging setups was the limiting factor in the collection of the higher-resolution data.

Setup descriptions:

1. **3.0^3 and 0.7^3 μm^3 voxel micro-X-PCI-CT data collection:** the single-distance propagation-based (PBI) X-ray phase-contrast CT imaging setup of the ID17 Biomedical beamline[6] of the European Synchrotron (ESRF - Grenoble, France) was used to collect full-organ mouse brain X-PCI-CT data with effective voxel size of 3.0^3 μm^3, as well as subsequent partial-organ maps with the 0.7^3 μm^3 voxel optics system. All 15 half brain samples from aged animals (7 3xTgAD and 8 B6/129 WT mice) were imaged in their entirety with the 3.0^3 μm^3 setup, and 7 of those samples were imaged with the 0.7^3 μm^3 setup, aiming local CT scans to dorsal and ventral CTX and HIP brain regions. 3.0^3 μm^3 voxel micro-X-PCI-CTs were performed in the imaging hutch of the ID17 beamline, around 150 m away from a wiggler X-ray source, using a quasi-parallel, quasi-monochromatic 30 keV X-ray beam, obtained from a Si double Laue crystal monochromator system[7], and 1 mm Al and 0.8 mm Cu as absorption filters. 0.7^3 μm^3 voxel micro-X-PCI-CTs were instead performed in the so-called MRT hutch of the ID17 beamline, 45 m away from the wiggler, using a pink X-ray beam with peak at 40 keV and a ~20 keV broad spectrum. In this case, movable absorber filters included aluminum (1 mm), copper (0.7 mm) and carbon (1.15 mm). Both setups use a

sCMOS PCO.Edge 5.5 (PCO AG, Germany) detector camera and a YAG-based scintillator, coupled to a x1:2 optic system to obtain the 3.0^3 μm^3 effective voxel size (sample-to-detector distance set to 180 cm), and to a x1:10 optic system to obtain the 0.7^3 μm^3 effective voxel size (sample-to-detector distance set to 50 cm). The 2560 x 2160 2D pixel array of the detector allow a field-of-view (FoV) at 3.0^3 μm^3 of about 7.7 mm x 6.5 mm (H x V), at 0.7^3 μm^3 of about 1.9 mm x 1.5 mm (H x V).

<u>3.0^3 μm^3 voxel CT scan parameters:</u> 300 ms detector exposure time, 2500 projections, standard 180-degree full-tomography with a position of the axis of rotation at the center of the projection images. Two consecutive partially-overlapping CT acquisitions, after vertical displacement of the sample with respect to the X-ray beam, were sufficient to measure the mouse half brains in their entirety. CT scan time: ~ 12 min.

<u>0.7^3 μm^3 voxel CT scan parameters:</u> 100 ms detector exposure time, 3000 projections, 360-degree tomography in half-acquisition mode, with a rotation axis position displaced to near the edge of the projection, almost doubling the horizontal effective CT FoV to ~ 3.5mm x 1.5mm (H x V). Several local-CT scans were performed for each sample. CT scan time: ~ 5 min.

<u>ID17 data CT reconstruction:</u> tomographic reconstructions were performed using the ESRF PyHST2[8] software package via a standard filtered-back projection after application of the single-distance Paganin[9] phase-retrieval algorithm (PyHST2 Paganin-length parameter: 300).

2. **<u>0.3^3 μm^3 micro-X-PCI-CT data collection:</u>** the single-distance PBI X-PCI-CT imaging setup of the TOMCAT beamline[10,11] of the Swiss Light Source (SLS, Paul Scherrer Institute,

Villigen, Switzerland) was used to collect cellular-resolution local brain-tissue X-PCI-CT data using an optical system affording an effective voxel size of 0.3^3 μm^3. Four aged brain samples (2 3xTgAD and 2 WT mice) plus the 1 young brain sample (WT mouse), were imaged by aiming local CT scans at dorsal and ventral CTX and HIP layers. Acquisitions were performed with a 5 cm sample-detector distance, using a quasi-parallel quasi-monochromatic 21 keV X-ray beam, obtained with a W/Si multilayer monochromator, and 100 µm Al and 10 µm Fe filters. A sCMOS PCO.Edge 5.5 detector camera, coupled to an Optique Peter microscope at 20x magnification and a 20 µm-thick LuAG:Ce scintillator, was used to obtain an effective voxel size of 0.325^3 μm^3. The 2560 x 2160 2D pixel array of the detector affords a FoV of ~ 0.86 mm x 0.70 mm (H x V).

<u>0.3^3 μm^3 voxel CT scan parameters:</u> 120 ms detector exposure time, 3000 projections, standard 180 degree full-tomography with central rotation axis position. CT scan time: ~ 6 min.

<u>TOMCAT data CT reconstruction:</u> tomographic reconstructions were performed via filtered-back projection after application of Paganin's single-distance phase-retrieval algorithm, using in-house computing resources available at TOMCAT.

3. **<u>0.1^3 μm^3 nano-X-PCI-CT data collection:</u>** the X-ray nanoholotomography (XNH) imaging setup of the ID16A nano-imaging beamline[12–15] of the ESRF was used to collect local brain-tissue X-PCI-CT data with effective voxel sizes of 0.1^3 μm^3. 3 aged brain samples (2 3xTgAD and 1 WT animals), were imaged by aiming local CT scans at dorsal CTX and HIP layers within dissected 2 x 2 x 4 mm^3-volume tissue biopsies. The ID16A XNH setup[13,14] uses

multilayer-coated Kirkpatrick–Baez mirrors to focus the beam to a high-brilliance ~ 30 x 30 nm^2 spot, which enables imaging with sub-100 nm spatial resolution. The beam energy was set to 17 keV, with a monochromaticity of 1%. At a fixed focal-plane-to-detector distance of ~1.2 m, projections images were recorded with a 4kx4k fast readout and low noise CCD camera (FReLoN, ESRF), binned to 2048 x 2048 pixels and coupled to magnifying optics and a 23 µm thick GGG:Eu scintillator. The voxel size, given by the geometrical magnification, was set to 0.1^3 $µm^3$ and the corresponding field of view was 0.2 mm x 0.2 mm (H x V). Samples were placed in vacuum (~10^{-7} mbar) on a rotating stage downstream of the focal plane. Single-distance low-resolution CT overview-scans with a voxel size of ~200^3 $µm^3$ helped locate the most relevant CTX and HIP regions, within the extended rod-like samples, which were then further imaged at 0.1^3 $µm^3$ voxel size.

0.1^3 $µm^3$ voxel CT scan parameters: sets of angular holograms[16] were recorded at 4 different (pre-defined) propagation distances[12,17,18] (starting with a 40 mm focal-plane-to-sample distance, and then moving the sample progressively closer to the detector) by rotating the sample over 180 degrees. Exposure time for each projection was 250 ms and 2000 projections were recorded for each tomographic sub-scan. Total scan duration was around 4 h.

ID16A data CT reconstruction: projections were normalized, rescaled to the smallest pixel size, and registered via an in-house algorithm based on cross-correlation. Assuming pure phase object samples, a 4-distance contrast-transfer-function-based algorithm for phase-retrieval was applied[18,19], including Wiener regularization[20] to improve low frequencies, using GNU Octave software. The final 3D datasets were obtained from angular phase-maps through filtered-back projection CT reconstruction, using PyHST2[8] software.

X-PCI-CT data post-processing and analysis:

CT artefacts: CT cupping artefacts related to local-tomography acquisitions were removed by flattening reconstructed CT images via normalization against their Gaussian-blurred version (filter sigma size: 50). CT ring artifacts were in large part removed from reconstructed CT images with an ESRF in-house post-processing tool[21].

MIPs: maximum intensity projections (MIPs) were computed by summing 20-100 consecutive CT images via the maximum intensity z-projection function in ImageJ[22]. This approach highlights hyper-intense image features and creates a pseudo-volumetric rendering by projecting the brightest voxels within a 3D stack onto a 2D image.

LUT-recoloring of X-PCI-CT data: different look-up tables (LUTs) available in ImageJ were used in the recoloring of X-PCI-CT images (e.g. 6-shades, Orange Hot, Viridis LUTs).

3D renders: all 3D renderings were obtained using the commercial software VG Studio Max 3.2 (Volume Graphics GmbH, Heidelberg, Germany). Volumetric extraction and segmentation of different tissue features (e.g. hyperdense particles, vasculature, normal parenchyma) were performed with the threshold-based gray-value-range voxel selection option or the region-growing voxel selection option. The threshold-based tool was used for hyperdense (HD) particle features and hypodense vasculature features. Normal brain cells and plaque-like deposits were instead extracted from inverted-gray-level X-PCI-CT maps via manual selection and the region-growing tool. Color-coding was achieved with customized color presets, generally rendering HD particles in white, vasculature in red and normal parenchyma in azure. 3D renderings were then

obtained via either the non-transparent 'Isosurface' or the semi-transparent 'X-ray' rendering algorithms.

Automated 3D segmentation of hyperdensity (HD) within X-PCI-CT data: threshold-based segmentations of HD particles were made automatic by using the auto-thresholding tool in ImageJ and by applying the maximum entropy auto-threshold algorithm[23] (MaxEntropy AutoT), which has already been shown to work on X-PCI-CT data for the segmentation of macroscopic amyloid deposits[2]. We computed the MaxEntropy AutoT algorithm slice-by-slice on every 10th CT image in a volume of interest, and averaged the obtained oscillating threshold values to choose a single threshold level for the entire volume. In this way, differently-sized HD particles could be extracted.

Quantification of hyperdensity (HD) within X-PCI-CT data:

a. **Macroscopic plaque-like deposits**: to obtain a one-off proof-of-principle X-PCI-CT-based Aβ plaque quantification, macroscopic senile plaque-like HD objects (n=27) were segmented-out of X-PCI-CT datasets from one WT and two 3xTgAD mice, using the manual region-growing tool in VG Studio Max. The volume of each extracted plaque-like HD objects, obtained by counting the voxels within each object mask, was used to compute the object's equivalent-sphere diameter. Diameter values from different objects were combined to obtain a distribution and calculate a mean size for these plaque-like HD objects.

b. **HD cell-like particles**: to obtain a systematic quantification of particle-like HD within HIP and CTX layers in 3-0.1 μm pixel X-PCI-CT data, every volume-of-interest (VoI) was

segmented via the automatic threshold-based approach described above, and the automatically-computed threshold value was used as lower limit to compute a volumetric mask of the data. The segmented mask was then analyzed via the '3D Object Counter' ImageJ plugin[24], which extracts individual unconnected 3D particles and computes their volumes. Just as for macroscopic plaques, the equivalent-sphere diameter was calculated from the volume of each individual HD particle as a way to express object sizes, and all computed diameters were combined to obtain the distribution of particle-like HD 3D objects within the VoI. Objects with diameters smaller than twice the pixel size (i.e. non-resolved objects) were excluded from distributions. Moreover, the few outlier objects with diameters greater than 30 μm were also excluded from distributions, because deemed to arise from non-cell-like objects (possibly image artefacts, or larger anatomical brain features unrelated to cellular neurodegeneration). Three different quantitative parameters were extracted from HD particle size distributions:

i. HD particle 3D load: the proportion of brain tissue within a VoI pertaining to HD particles, computed by summing the volumes of all extracted individual particles and then normalizing with respect to the total volume of the VoI. Load was then expressed as the percentage of the total volume occupied by the HD particles.

Tri-modal HD-particle size distributions from 0.7^3, 0.3^3 and 0.1^3 μm^3 voxel-size X-PCI-CT data were further analyzed, by fitting to them a 3-component Gaussian mixture model (GMM) and by using the 'fitgmdist' function in Matlab (MATLAB R2018a, The MathWorks, Inc., Natick, Massachusetts, United States), a function that implements an iterative Expectation-Maximization (EM) algorithm[25]. A 2-component GMM was instead fitted to the bimodal distributions obtained from

the 3.0^3 μm^3 voxel-size X-PCI-CT data. In this way, two additional HD-particle parameters could be obtained:

ii. <u>HD particle populations mean sizes:</u> the 3-component (2-component) GMM fit of the HD-particle diameter distributions returns three (two) Gaussian means (mu parameters), which express the mean sizes of each of the three (two) cell populations.

iii. <u>HD particle population proportions</u>: the 3 (2) mixing proportions returned from the 3 (2)-component GMM fit of HD-particle diameter distributions, which express the proportion of each HD particle cell population within a VoI.

<u>3D virtual tissue sample collection:</u> sub-volumes of interest (sVoI) were collected from each 3D brain X-PCI-CT dataset, in order to sample four specific brain regions, i.e. dorsal CTX (dCTX), dorsal HIP (dHIP), ventral CTX (vCTX) and ventral HIP (vHIP). sVoI centers were positioned at the following bregma-based stereotactic coordinates (in mm), ML being medial-lateral, AP anterior-posterior, and DV dorsal-ventral, as annotated in the Paxinos Atlas[26]:

a. dCTX: center ~ ML +0.7, AP -2, DV +0.7

b. dHIP: center ~ ML +0.7, AP -2, DV +1.5 (HIP CA1sp)

c. vCTX: center ~ ML +4, AP -3, DV +4

d. vHIP: center ~ ML +2.9, AP -3, DV +4 (HIP CA1sp).

sVoIs were sampled from all mice involved in the study and from all the different resolution datasets, affording a total of 90 virtual-histological 3D tissue volumes. The analysis included 60

sVoIs extracted from 3.0^3 μm^3 voxel X-PCI-CTs (all 15 mice were measured at this resolution, so that all 4 regions per animal could be sampled), 16 sVoIs from 0.7^3 μm^3 voxel CTs (7/15 mice measured, dorsal and ventral regions sampled), 8 sVoIs from 0.3^3 μm^3 voxel CTs (4/15 mice measured, dorsal regions sampled) and 6 samples from 0.1 µm pixel CTs (3/15 mice, dorsal regions sampled).

<u>3D Quantification:</u> volumetric HD particle masks were automatically segmented from each of the (90) sampled sVoIs. As discussed above, individual 3D masks (from each sVoI) were analyzed to extract several sample parameters: HD-particle load, mean HD-particle sizes for three different cellular populations, and HD-particle population proportions.

<u>Quantitative group comparisons:</u> Region-based and animal-group-based averaging of the 3 extracted parameters (load, particle sizes, particle proportions) were performed to study potential differences in the quantified levels of HD-based tissue neurodegeneration between different animal types, different treatment types and different brain regions. Group distributions (and group averages) were obtained by combining the individual parameters of all sVoIs pertaining to a specific group. Possible significant differences in parameters between animal groups were tested by one-way ANOVA testing, with Turkey-Kramer's multiple comparison post-hoc test for unequal sample sizes, o by unpaired two-sided two-sample T-test, using SAS software (SAS Studio, SAS University Edition 2.7, SAS Institute Inc., Cary, NC, USA). Group differences related to calculated P-values of less than 0.05 were deemed significant. In addition, differences between samples of data with different voxel-sizes were explored qualitatively (no ANOVA testing performed).

Data displays: Quantification displays (histograms, bar graphs, pie charts) were all produced with the commercially available software GraphPad Prism (GraphPad Prism 8.0.0, GraphPad Software Inc., San Diego, CA, USA).

XFM measurements:

Sample preparation: half brains from one 13 month old WT and one 13 month old 3xTgAD were sliced with a Leica RM2265/LN22 ultra-microtome (Leica Microsystems), using diamond blades, into 6-µm-thick coronal sections. The vertical sample holders consisted of 7x7 mm^2-wide, 200 µm-thick silicon frames with 3x3 mm^2-wide, 500 nm-thick silicon nitride (Si_3N_4) windows (Silson Ltd). Sectioned brain slices were loaded onto the windows after brief soaking of the slices in water.

XFM data collection: 2D scanning nano-XFM measurements were performed in vacuum at room-temperature conditions using the synchrotron setup with sub-cellular resolution available at the ID16A beamline[14,27–30] of the ESRF. The samples were raster scanned with a beam of 17 keV X-ray excitation energy (1% bandwidth), focused to ~30 x 30 nm^2 (H x V). Detected spectra were recorded with a pair of six element silicon drift diode X-ray fluorescence detectors (SDDs, SGX Sensortech, UK). This high-brilliance nanoscopic XFM setup can map trace elemental distributions quantitatively in single cells and intracellular compartments, within tissue slices, in close to native state. It was also possible to follow-up XFM measurements with coherent X-PCI radiographic imaging of the same sample areas, to obtain additional information on cellular density. A visible-light microscope was used to aim XFM scans toward cells within HIP and CTX layers, and a quantitative calibration of elemental area density was performed with a thin film reference

sample (AXO Dresden GmbH) containing mass depositions in the range of ng/mm^2 (1-3 atomic layers).

Coarse XFM raster-scans, acquired to further perfect scan aiming, were performed with a 400x400 nm^2 step size and 100 ms dwell time. Definitive 2D fine raster-scans instead were performed with a 120 x 120 nm^2 step size and 50 ms dwell time. FoVs of the fine-scans were in the order of ~ 35 x 35 µm^2, obtained from ~ 85k individual XFM point spectra. Ten 2D fine scans were collected per brain sample, and approximately equal amounts of cortical and hippocampal cells were measured. For each scan, local intracellular K, S, P, Zn, Fe, Cu, Ca and Br elemental content maps (in ng/mm^2) were obtained in addition to one 2D X-ray phase-map of the same area.

<u>XFM data post-processing:</u> The quantitative elemental area density maps were obtained from individual point spectra, by fitting and normalizing the data using the PyMCA-software for XFM spectral analysis[31] available at the ESRF, including an attenuation correction (significant for low Z elements), which assumed a 500 nm-thick silicon nitride sample matrix. The quantification is based on a calibrated fit using the PyMCA library. The energy calibration (10eV per channel) was verified with PyMCA on the sum spectrum of several XRF maps. No emission peak shift due to strong scattering peaks was observed, since the tissue sections were very thin (~6 microns), the regime of high counting rates was systematically avoided, the detector geometry was optimized and the experiment was performed under vacuum. LUT-based recoloring of the quantitative elemental XFM maps was performed with ImageJ, using the Magenta Hot LUT for P & S and the Green-Fire-Blue LUT for Ca/Fe maps. Co-localization XFM maps were recolored with the Magenta (for P), Yellow (for S), Red (for Ca), and Green (for Fe only) LUTs.

XFM image analysis and quantification: Normalized and calibrated XFM elemental density data were used to extract individual 2D cell masks, by manual selection of intracellular areas in ImageJ of 22 normal cells and 11 ICHD-bearing cells. The same 2D mask was used for all the different elemental density maps, and the phase-map, pertaining to a specific cell. The labels 'normal' vs. 'ICHD-bearing' were assigned to cells by manual reader inspection of the gray-levels and morphology visualized in the phase maps. For each masked cell and for each interrogated element, the mean intracellular 2D elemental density was computed using ImageJ. By combining measurements from all cells in each of the two groups, we obtained distributions of intracellular elemental content for each interrogated element. Possible significant differences in the average elemental density between the two cell groups were tested by unpaired two-sided two-sample Wilcoxon Test, using SAS software (SAS Studio, SAS University Edition 2.7, SAS Institute Inc., Cary, NC, USA). Group differences related to calculated P-values less than 0.05 were deemed significant. Bar graphs were produced with commercially available software GraphPad Prism (GraphPad Prism 8.0.0, GraphPad Software Inc., San Diego, CA, USA).

High-field MRI measurements:

Sample preparation: PBS-stored hydrated half brains from one 13 month old WT and one 13 month old 3xTgAD were embedded in 2% agarose gel in PBS and each placed in a 2 ml Eppendorf Safe-Lock tube. After an initial non-contrast-enhanced (non-CE) high-field MRI-imaging session, the agarose gel was removed and the samples were prepared for contrast-enhanced (CE) MRI (following a reported contrast-enhancement approach[32]). Briefly, samples were first incubated for 24 hrs in PBS containing 0.1% gadoterate meglumine (Dotarem, 0.5 mmol Gd/mL; Guerbet, Aulnaysous-Bois, France); then, the buffer was replaced and the incubation continued for

another 72 hrs. Finally, the brains were placed again in 2 ml Eppendorf Safe-Lock tubes filled with Fomblin (Fomblin® Y; Sigma-Aldrich, Munich, Germany).

MRI data acquisition: a preclinical 9.4 T MRI scanner (BioSpec 94/21 USR; Bruker, Ettlingen, Germany) was used for data collection. Coronal brain images were collected using the manufacturer's transceiver cryogenic quadrature RF surface probe (CryoProbe, catalog number Z125365, Bruker Biospin, Ettlingen, Germany). The images were acquired with the following parameters:

A. Non-CE MRI: 3D FISP sequence; repetition time (TR), 29 ms; echo time (TE), 12 ms; excitation pulse angle 20°; FOV, 8 x 8 x 8 mm^3; acquisition matrix, 440 x 440 x 440; spatial resolution, 18.2 x 18.2 x 18.2 μm^3; number of averages, 22; oversampling slice direction, 30%; bandwidth, 35714 Hz; scan time, 44 hrs 36 min.

B. CE MRI: 3D FISP sequence; repetition time (TR), 28 ms; echo time (TE), 11 ms; excitation pulse angle 40°; FOV, 6 x 7 x 10 mm^3; acquisition matrix, 307 x 358 x 512; spatial resolution, 20 x 20 x 20 μm^3; number of averages, 9; bandwidth, 32467 Hz; scan time, 15 hrs 22 min.

TEM measurements:

2 x 2 x 4 mm^3 biopsies of CTX and HIP layers from one 13-months old 3xTgAD and one 13-months old WT animal, already used for 0.1^3 voxel X-PCI-CT, were dehydrated through a graded ethanol series (50/70/90/100%), substituted with a mixture of resin and 100% ethanol (50/50 vol/vol),

embedded in Embed 812 resin (EPON substitute, EMS) overnight at RT, and then put for 2 days at 65°C for polymerization. Ultrathin sections (100 nm or 200 nm) were cut with a Leica EM UC7 ultra-microtome (Leica Microsystems), collected on formvar-coated copper grids and counterstained with uranyl acetate 2% and lead citrate 2%. Sections were observed using a Tecnai12 G2 Spirit Bio Twin (ThermoFischer) microscope operating at 120 kV using an Orius SC1000 CCD camera (Gatan).

Histology and immunohistochemistry:

Coronal sections (10 µm) were cut on a microtome and processed for Thioflavin-S staining. Briefly, sections were washed in PBS and stained with Thioflavin-S (Sigma-Aldrich, St. Louis, MO) at 1% (w/vol) in 70% ethanol for 10 min. Finally, sections were washed in 50% ethanol and in water, then dried and cover-slipped with Anti-fade mounting media with Dapi for nuclear staining. For immunohistochemistry, sections were deparaffinized and rehydrated by standard procedures. After antigen retrieval in citrate buffer (10 mM, pH 6.0) for 15 min, samples were pretreated with 3% H_2O_2/3% methanol for 10 min to block endogenous peroxide activity, and then blocked in 5% normal serum and 0.1% Triton-X100 in PBS for 2 hrs. Sections were incubated with the following primary antibodies: anti-β-Amyloid 17-24 clone 4G8 made in mouse (1:100; Biolegend, S. Diego, CA; RRID: AB_2564633), anti-phospho-tau pSer404 made in rabbit (1:100, Sigma-Aldrich, S. Louis, MO; RRID: AB_261759), anti-NeuN made in rabbit (1:200, Millipore, Darmstadt, Germany; RRID: AB_10807945) and anti-NeuN made in mouse (1:200, Millipore, Darmstadt, Germany; RRID: AB_2298772) for double immuno-labeling. Sections were then rinsed and incubated for 2 hrs at room temperature with secondary antibodies: Cy3-conjugated donkey anti-mouse IgG (1:200, Jackson ImmunoResearch, West Grove, PA: RRID: AB_2340818) or donkey

anti-rabbit (1:200, Jackson ImmunoResearch, West Grove, PA: RRID: AB_2307443); biotinylated horse anti-mouse IgG (1:200, Vector Laboratories, Burlingame, CA; RRID: AB_2313581), or biotinylated horse anti-rabbit IgG (1:200, Vector Laboratories, Burlingame, CA; RRID: AB_2336201) followed by Streptavidin Alexa Fluor 488 (1:200, Molecular Probes, the Netherlands). Finally, samples were mounted with Vectashield with Dapi (Vector Laboratories, Burlingame, CA) and examined under a Zeiss 780 confocal laser scanning microscope. We used a 488 nm argon laser to excite Alexa 488, and 543 nm HeNe laser to excite Cy3.

Statistical Analysis:

All statistical analysis was performed using the SAS software (SAS Studio, SAS University Edition 2.7, SAS Institute Inc., Cary, NC, USA). All graphs were then produced with commercially available software GraphPad Prism (GraphPad Prism 8.0.0, GraphPad Software Inc., San Diego, CA, USA).

1. The XFM data (**Figure 5e**) was pre-processed as described in methods section "**XFM measurements**". Two animals were used in the analysis (one 3xTgAD at 13 months, one WT at 13 months). No outlier values were removed. Data represent mean intracellular elemental content in two groups (normal cells vs. ICHD cells) for elements P, S, Ca and Fe, and are presented in a bar graph, which plots mean 2D elemental density (bars) ± SD (error bars). Sample sizes in the two groups are n=22 cells for normal cells, n=11 cells for ICHD cells. Statistically significant differences were tested by unpaired two-sided two-sample Wilcoxon Test, with P-values less than 0.05 considered significant.

2. The quantification workflow for HD particles was performed as described in methods section "**X-PCI-CT data post-processing and analysis**". Experimental animal groups (13

month old mice) included WT treated with saline (n=4 animals), WT treated with LY379268 (n=4), 3xTgAD treated with saline (n=3), 3xTgAD treated with LY379268 (n=4). For each selected VoI (90 VoIs in total), HD objects with diameters smaller than twice the pixel size (non-resolved objects) and few outlier HD objects with diameters greater than 30 µm (non cell-like objects) were excluded from distributions. Data in **Suppl. Figure 12e** represent two example HD particle size distributions from one cortical and one hippocampal tissue volumes, presented in violin plots, which graph the sphere-equivalent diameter of each extracted HD particle within the two example tissue VoIs. **Figure 6b** plots the volumetric size of all the individual VoIs used in the analysis, and voxel-size group means (bars) ± SD (error bars). **Figure 6c** plots a bar graph of an example tri-modal HD particle size distribution extracted from one of the VoIs, fitted with a 3-component Gaussian mixture model to model HD particles as nucleoli, glia and ICHD neuron populations. **Figure 6d** presents pie charts of average HD-population proportions for each experimental group. **Figure 6e** presents a bar graph for experimental group mean particle sizes for each HD-population (bars) ± SD (error bars). **Figure 6f-g** present bar graphs of experimental group mean HD-particle 3D % load in (**f**) CTX vs. HIP and (**g**) dCTX vs. vCTX (bars) ± SD (error bars). Statistically significant differences in **Fig. 6d-g** were tested by one-way ANOVA with Turkey-Kramer's multiple comparison post-hoc test, with P-values less than 0.05 considered significant. **Suppl. Fig. 13b** presents a box plot quantifying the sphere-equivalent diameter of n=27 macroscopic extracted plaque-like HD clusters (plotted are minimum, maximum, median, first and third quartiles). **Suppl. Figure 14**

expands the data presentation in **Figure 6** on the quantification of intracellular HD in the four experimental animal groups, performing the same statistical testing.

SUPPLEMENTARY RESULTS:

Suppl. Fig. 8:

The morphological information in **Suppl. Fig. 8** suggests a likely difference in the biological nature of ICHD deposits in WT vs. 3xTgAD mice: on one side likely normal age-related accumulations in WT brains, and on the other side neurodegenerative protein accumulation leading to cell-death in the 3xTgAD mice.

Fig. 4:

The double IHC for Aβ and NeuN, observed especially in 13 month old 3xTgAD mouse brains (**Fig. 4b**), formed distinctive ring-like patterns around neuron nuclei, which match reported IHC work on 3xTgAD mice[33–35]. Similarly to other IHC studies on the 3xTgAD mouse[34–37], the double IHC for p-tau and NeuN (**Fig. 4d**), displayed little somatic p-tau involvement (hardly any co-localization of p-tau and NeuN signals), whereas strong p-tau-positivity within neuron dendrites and axons (p-tau IHC signal present in appendages of neuron somas).

Fig. 5:

Sample preparation (tissue fixation, paraffin embedding) prior to the XFM measurements may have affected the absolute 2D-density values of some of these elemental distributions, especially in the case of Ca[38], making them differ from in situ values. In the case of Fe, too, histochemical

methodological procedures are known to affect iron content, and to lead to content reductions in iron-rich brain areas and complete loss in brain areas of low iron content[39]. Overall, the XFM-detecting significantly higher density of P and S, Ca and Fe within ICHD-bearing cells may suggest an intracellular accumulation of either protein aggregates (likely due to normal aging processes in WT animals, and possibly due to hyper-phosphorylated p-tau fibrils or Fe-trapping Aβ oligomers in the 3xTgAD model) or the occurrence of other downstream cellular aging or neurodegenerative processes possibly elicited by the protein-build-up itself (e.g. cell condensation, apoptosis, necrosis, dystrophic mineralization of cell bodies or ferrugination). The identification and localization of iron within different brain regions is fundamental for the study of iron in neurodegenerative diseases where iron is involved in the pathophysiology.

Suppl. Fig. 11:

MRI is known to permit whole-brain mapping, from which cellular-level microstructures can be inferred[40]. MRI signal hypo-intensity is known to correlate within amyloid agglomerates, reportedly due to localized Fe trapped within the protein aggregates, and is used both postmortem and *in-vivo*[41,42] as a morpho-functional marker for AD-linked neurodegenerative processes in the brain of animal models[43] (including the 3xTgAD mouse[44]) and in humans[45].

Differently from X-PCI-CT, TEM involves use of heavy metal salt staining, embedding in Epon and sectioning of brain-tissue in 100-200 nm thick slices. In tissues from aged WT (**Suppl. Fig. 11d-e**) and aged 3xTgAD animals (**Suppl. Fig. 11f**), both techniques visualize euchromatin-rich hypodense nuclei with smooth spherical nuclear envelopes and dense nucleoli.

Fig. 6:

Merging results from all sample-volumes, we obtained distributions of each parameter for each experimental group (respectively **Fig 6d, 6e, 6f-g**). The volumetric load of ICHD-particles was studied both overall between animal groups (**Suppl. Fig. 14**), as well as regionally (in CTX and HIP layers, **Fig. 6f**) and sub-regionally (in dorsal and ventral CTX layers, **Fig. 6g**).

The influence of different image voxel sizes in the dataset of this multiscale analysis was found to, naturally, affect object-density calculations: higher resolving powers allowed the detection of smaller, and thus of more objects per unit-volume. This did not invalidate the calculations of morphological (particle size) and histopathological (3D load) parameters, which afforded similar results at all imaging-system resolutions (**Suppl. Fig. 14**).

SUPPLEMENTARY DISCUSSION:

Possible artifacts in the X-PCI-CT data:

X-PCI-CT scans, especially at the highest spatial resolutions, deposit conspicuous energy locally (from hundreds to thousands of milligrays, depending on the used pixel size), which may lead to sample heating, local dehydration and shrinkage. Moreover, X-PCI-CT data of fixed paraffin-embedded samples may suffer the same differential neuropil shrinkage artifacts typical of histological work.

Non-specificity of the ICHD signal in X-PCI-CT maps vs. other techniques:

Image-contrast in most of the phase-retrieved X-PCI-CT datasets presented here, after application of the Paganin phase-retrieval algorithm[9], is only semi-quantitative. In terms of

relative density levels, the ICHD-bearing particles appeared as denser than adjacent normal brain parenchyma, but also as less dense than typical high-Z bio-materials (e.g. bones, iron-rich blood-filled vessels, or calcifications[46]), and of a comparable density to that of neuron nucleoli. This signal intensity suggests a likely protein-based composition for the abnormal aggregates along with possible mild metal involvement (e.g. protein-bound Ca or Fe), all intracellular contents expected in aged and neuro-degenerating brains.

X-PCI-CT-measured signal within ICHD neurons is non-specific and could be a reporter of different cellular conditions, both AD-linked and non-AD-linked[47]. This is not dissimilar to the signal aspecificity of other known histological markers of acute cellular neurodegeneration, e.g. 'dark' argyrophilic Gallyas-silver-positive neurons, which aspecifically report cellular neurotoxicity[48,49], or Fluoro-Jade-positive, which aspecifically report degenerating neurons[50,51], or even dark-stained neurons in toluidine blue histological sections, which aspecifically report likely cellular condensation[52–54]. Overall, the ICHD tissue markings in our data on 3xTgAD mice closely resemble tissues stained with a Campbell-Switzer impregnation, a semi-specific dye for AD neuropathology[54].

Morphology of ICHD vs. biological considerations:

The correlation of morphological observations to known AD micro-biology enables the association of different ICHD traits in 3xTgAD mice with different expressions of aging-related AD neurodegeneration: the ring-like patterns of cytoplasmic ICHD accumulations match immuno-labeling patterns typical of cellular amyloidopathy[33]; dendritic and axonal ICHD, instead,

resemble classic patterns of cytoskeletal tauopathy[55], which predominantly feature the fibrillization of axonal microtubules (NFT formations). Observed cell-shrinkage, blebbing, and nuclear laminopathy, for their part, are likely linked to protein-induced downstream mechanisms of cell death, which involve somatic condensation, e.g. cell pyknosis during apoptosis or dystrophic mineralization (calcification and ferrugination) during cell necrosis. Glial involvement, for its part, suggests possible occurred local gliosis, i.e. another process typically present in aging and degenerating brains. In sum, most of the distinctive morphological features of ICHD-bearing cells can be connected to known mechanisms of Aβ and p-tau toxic protein clumping during AD neurodegeneration or to neuronal aging.

Interest of cellular-level AD imaging in animal models:

Cellular-level visualizations of the smallest micrometric intracellular lesions in early stages of AD, even in animal models, are valuable to both studies on mechanism and on pharmacological treatment of AD. As it pertains to disease mechanism, AD animal models have long been used to help characterize early disease phases, even though e.g. mice have a short lifespan without a clear-cut neurodegenerative phenotype. As it pertains to AD-drug discovery, preclinical research on mouse models has highlighted how beneficial effects can arise with early treatment[56], a realization which has shifted the focus of some new drug tests toward the pre-symptomatic disease phase and on patients at risk for AD (e.g. solanezumab[57] drug-trial programs).

Aβ and p-tau IHC in the 3xTgAD mouse:

The patterns of protein deposition observed in the collected IHC are unsurprising, since the 3xTgAD mouse model is known[58] to present co-localized intracellular p-tau and Aβ toxic fibrils.

Extra- and intracellular Aβ deposits are reported to arise first, starting in CTX layers and then spreading to HIP ones. P-tau fibrillary buildup, instead, was proved successive to the amyloidopathy, accumulating intracellularly, first in HIP and then in CTX, and often co-localizing with the Aβ-positive cells. More generally, tauopathy and amyloidopathy are known to coexist in age-related processes of neurodegeneration in many animal models.

More on the XFM data:

The collected XFM data (**Fig. 5**) showed significant excess P and S 2D-density in both somatic and dendritic compartments of ICHD-bearing cells compared to normal cells. These higher concentrations are indicative of either intracellular agglomeration of dense protein deposits, or more simply of cell shrinkage of aged or degenerated cells, with a redistribution of the elements within smaller cell volumes.

Significant excess in cytoplasmic P and S content leads to an increase in overall local intracellular density, to which X-PCI-CT is sensitive[16], and may be indicative of intracellular agglomeration of dense AD-linked protein deposits. The intracellular hyper-accumulation of high-Z elements, such as Ca and especially Fe metal, also contributed to an increase in local intracellular electron density and concurred to produce the observed ICHD.

Within normal cells, postmortem XFM likely detects mostly Ca and Fe that is bound to protein (eg. Ca to calbindin, Fe to ferritin) or other biomolecules (e.g. Fe to dopamine)[27,29], since Ca^{2+} ions mostly leak out of cells post-fixation and redox metals (such as Fe) do not appear free in solution within normal cells (this would cause toxic oxidative stress). Within abnormal ICHD-bearing cells,

instead, postmortem XFM could detect both free Fe or Fe trapped within larger protein clumps, and is a clear indication of the occurrence of intracellular dis-homeostasis or neurodegeneration.

Of note is how nucleoli, regulation centers of ribosomal neurogenesis, neuronal growth and stress response[59], and recognizable as single dense and rounded intra-nuclear structures, feature P, Ca and Fe elemental hyperdensity in both ICHD-bearing cells and normal cells (**Fig. 4**). The smaller round-shaped deposits are normal and physiological, since P is a constituent of ribosomal DNA, Ca signals regulate normal gene transcription[60] and Fe reveals itself in the nucleoli of many normal CAsp neurons[61]. The more extended and diffuse intra-nuclear and near-nucleolar P, Ca and Fe areas within ICHD-bearing cells (**Fig. 4**), instead, seem pathologic/abnormal, likely driven by nuclear or nucleolar dysfunction and leading to neurodegeneration[59].

It is known that Fe recycling occurs also within lysosomes, which therefore generally contain relatively large amounts of redox-active iron[62]. Studies indicate that the formation of neuron-aging-related lipofuscin granula can be due to the oxidative alteration of macromolecules by oxygen-derived free radicals generated in reactions catalyzed by redox-active iron of low molecular weight[1]. Fe thus clearly plays an important role in both aging and neurodegenerative processes, and interestingly appeared as a significantly hyper-abundant metal in our XFM datasets on ICHD-cells in aging and AD mice. Overall, the XFM data suggest that both non-metallic and metallic intracellular elemental hyperdense deposits are contributing to the increase in intracellular electron density measured within ICHD-bearing cells via X-PCI-CTs.

Novel fixation and embedding protocols established for X-ray spectroscopy and microscopy[63,64] may lead to more reliable absolute measurements of metal content within biological tissues in similar future XFM measurements.

More on TEM results:

The observed nuclear membrane infoldings, in literature referred to as 'nucleoplasmic reticula', can occur either in certain types of healthy cells[65], or in cells suffering from neurodegenerative laminopathy, which leads to aberrant forms of nucleoskeletal invagination, to heterochromatin relaxation and to neuronal death. Interestingly, tau-induced nuclear envelope invagination[66] and lamin dysfunction have been observed in several tauopathy models[67], including AD models[68,69].

Role of group II metabotropic glutamate receptors:

The role of group-II metabotropic glutamate receptors (mGlu2 and mGlu3 receptors) in influencing mechanisms of neurodegeneration is debated, since activation of mGlu2/3 receptors by selective ligand agonists (e.g. LY379268 or LY354740) has downstream effects that could lead both to deceleration and to acceleration of neurodegeneration. mGlu3 receptors, for instance, have the potential to trigger neuroprotective effects and possibly decelerate neurodegeneration, since they can mediate the production of neurotrophic factors (e.g. transforming growth factor-β (TGF-β) and glial cell line-derived neurotrophic factor (GDNF)[70–76]) and limit the production of Aβ peptide by stimulating α-secretase activity[77]. Instead, selective activation of mGlu2 receptors has been shown to amplify neuron death processes in culture[72,76] and in experimental models of transient global ischemia[78]. Within microglial cells, mGlu2 receptors are known to activate the release of neurotoxic cytokines[79,80].

ICHD signal intensity and interpretation:

ICHD deposits exhibit levels of X-PCI-CT image gray-level hyper-intensity, at which normal cyto-architecture is still well visible. This observation implies that the two different density levels are somewhat comparable, and suggests an endogenous near-to-normal biological origin for the ICHD. More extreme levels of hyperdensity, e.g. within metals, would instead likely overpower X-PCI-based image contrast and hide the relatively-hypodense neuroanatomy of a normal nervous tissue. This is not the case, though, for the ICHD observed here. Therefore, an interpretation of the ICHD signal as protein aggregation seems fitting to its signal-level.

P vs. Fe contributions to X-PCI-CT signal:

Quantitatively, a higher absolute value of the mean P intracellular area density (~25 ng/mm^2) was observed within ICHD-bearing cells, compared to the value of the mean Fe intracellular area density (~1.4 ng/mm^2). This observation may seem to point to a stronger link between X-PCI-CT-detected ICHD and P accumulation, than between ICHD and Fe deposition, and therefore to strengthen an interpretation of the ICHD signal as of peptidic over metallic origin. Nevertheless, since X-PCI-CT phase maps are directly related to δ, i.e. the refractive index decrement in the X-ray complex index of refraction formula $n = 1- \delta + i\beta$, with δ proportional to the local electron density $\rho_{e,}$ and $\rho_e = Z/V = Z * \rho * N_a/A$ (Z atomic number, V volume of reference, ρ macroscopic mass density, N_A Avogadro's number and A atomic weight), an equivalent volumetric amount of a high-Z material, such as Fe, will contribute almost twice to X-PCI hyper-intensity compared to a low-Z material such as P (Z_{Fe}/Z_P=1.73). For this reason, the atomic properties of Fe make it a key player in the generation of the X-PCI-CT-observed ICHD signals.

Impact of sample shrinkage on data quantification:

Moderate systematic dehydration-related sample shrinkage, either due to sample-preparation (fixation, paraffination) or during X-PCI-CT acquisitions, may account for the slight discrepancy between literature and measured HD-particle sizes. Since all extracted brains were subject to the same sample-preparation workflow and X-ray irradiation imaging protocols, the comparative analysis of particle size between different animal groups still holds. Actually, 3D tissue virtual analyses (such as the X-PCI-CT-based one applied here), which avoid the lossy traditional histological approach (often leading to partial section analysis and interpolation) hold the promise of more complete and trustworthy neuropathological evaluations compared to more traditional approaches.

More on the results of the cellular-level quantification:

Also the measurements of HD-particle sizes generally matched literature values for cellular sizes in the mouse brain: diameters of nucleoli, glia and normal neuron somas are reportedly around 2 µm[81], 4-8 µm[82,83], and 12-18[84–86] µm in size respectively, compared to our X-PCI-CT measurements of about 2, 4 and 8 µm diameters, with the last value possibly suggesting mild somatic shrinkage of ICHD neurons.

SUPPLEMENTARY FIGURES & LEGENDS:

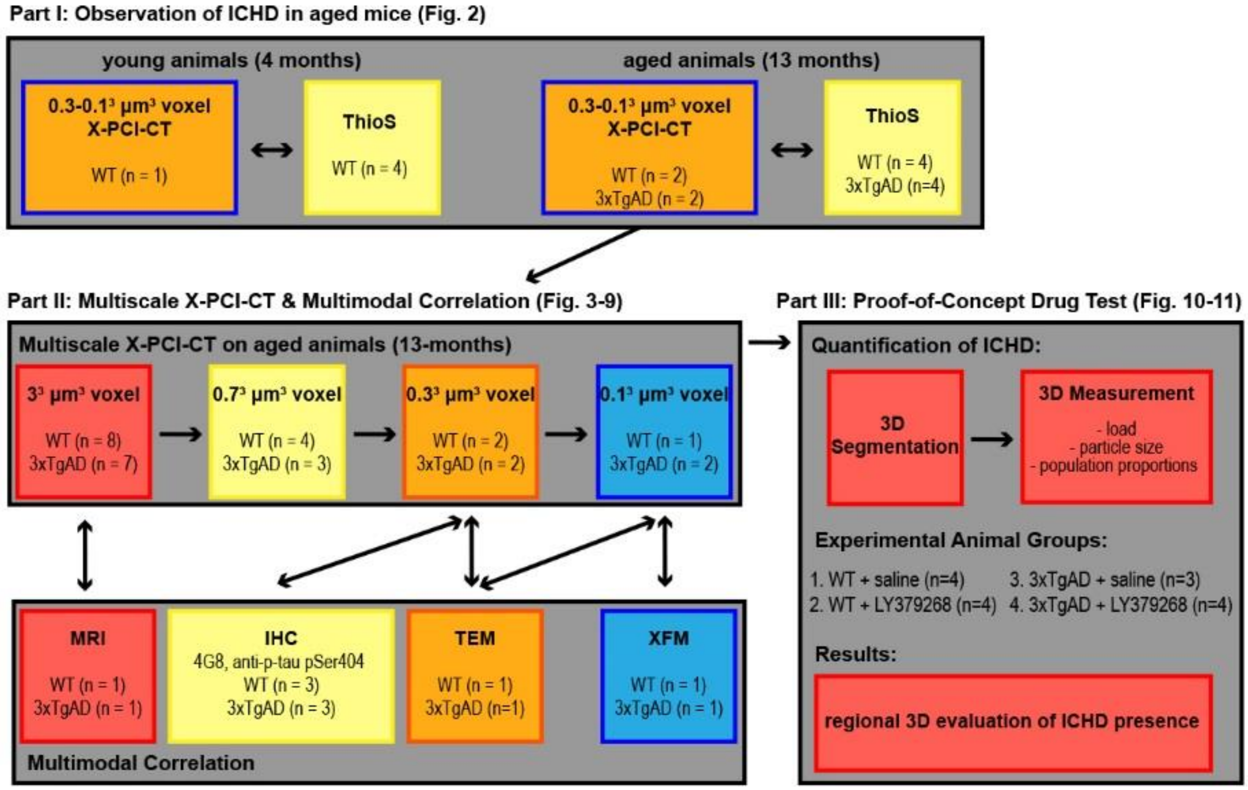

Suppl. Figure 1 | Study design flow diagram. Part I: ***Figure 1 | Study design flow diagram. Part I:*** *comparison of X-PCI-CT and Thioflavin S (ThioS) data from a young WT mouse (4 months old, n = 1) vs. aged WT and 3xTgAD mice (13 months old, WT n =2, 3xTgAD n =2). Only one (n=1) 4 month old WT mouse brain hemisphere was imaged at $0.3\text{-}0.1^3$ mm^3 voxel size, since the ThioS histological analysis showed homogeneity in several different animals (n=4).* ***Part II:*** *subsets of 13 months old aged WT (n=8) and 3xTgAD (n = 7) mice were analyzed via multiscale X-PCI-CT at four different spatial resolutions (3.0^3, 0.7^3, 0.3^3 and 0.1^3* μm^3 *voxels) and imaging results were compared to more well-established similarly-resolving neuroimaging techniques (MRI, immunohistochemistry/IHC, TEM) and to X-ray fluorescence microscopy (XFM).* ***Part III:*** *the same animals from* ***Part II*** *were divided into 4 experimental groups, after alternative treatment with LY379268 or saline solution, in order to showcase a proof-of-concept drug test and quantification of cellular hyperdensity based on the multiscale X-PCI-CT imaging approach explored in* ***Part I*** *and* ***Part II***.

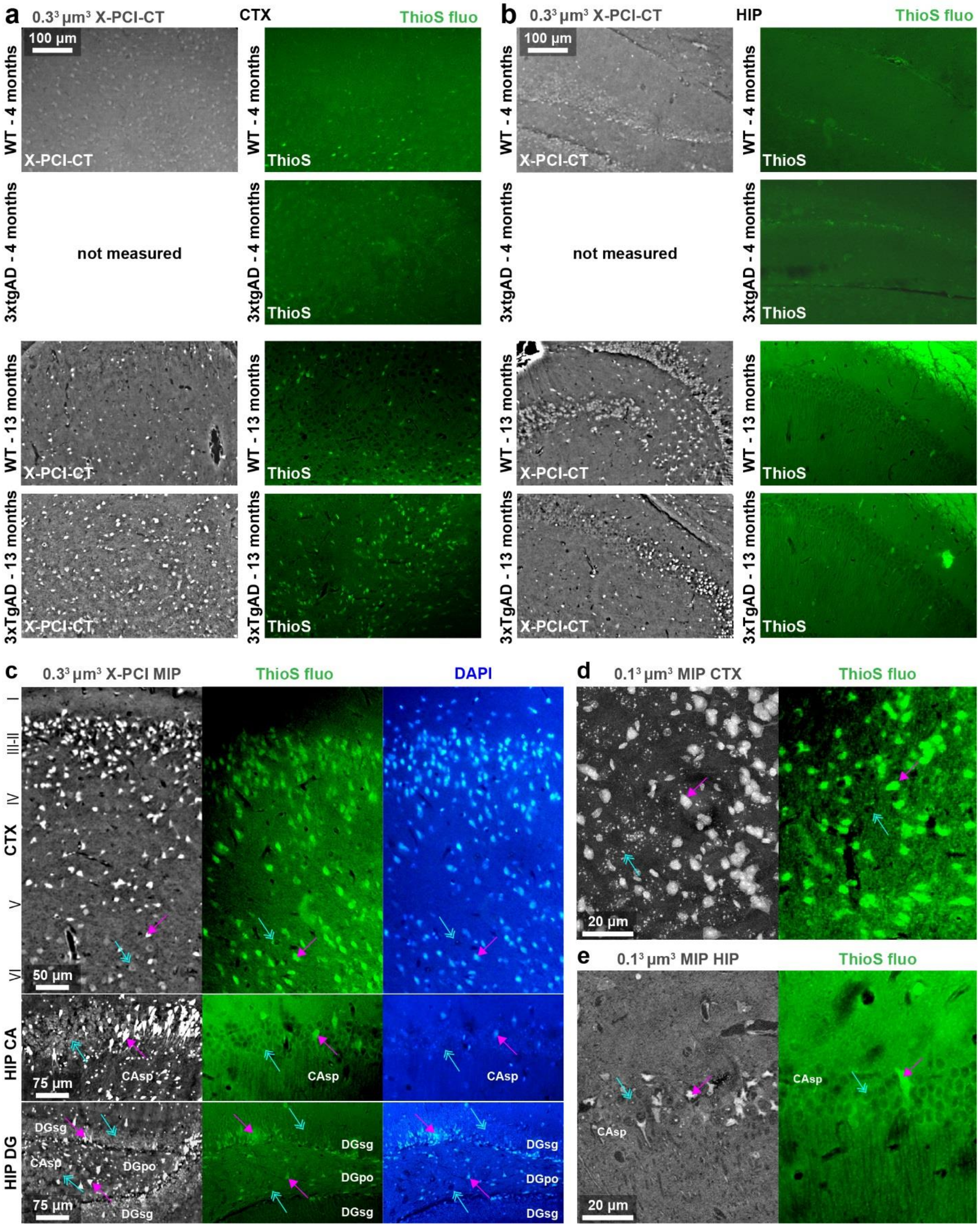

Suppl. Fig. 2 | Extended X-PCI-CT vs. ThioS fluorescence comparison. a-b, 0.3^3 µm^3 voxel X-PCI-CT vs. Thioflavin-S fluorescence (ThioS fluo) of comparable (**a**) CTX and (**b**) HIP cell layers in brain samples extracted from 4 month old WT and 3xTgAD mice, and from 13 month old WT and 3xTgAD mice. Patterns of HD-particles and ThioS-positivity

are similar. **c,** 0.3^3 µm^3 voxel X-PCI-CT vs. ThioS and DAPI fluo within comparable CTX, HIP CA and HIP DG cell layers from a 13 month old 3xTgAD mouse brain. **d-e,** 0.1^3 µm^3 voxel X-PCI-CT vs. ThioS fluo within comparable (**d**) CTX and (**e**) HIP cell layers from a 13 month old 3xTgAD mouse brain. In **c-e**, individual ICHD-bearing CTX I to VI neurons, and ICHD-bearing neurons populating CAsp and DGsg layers (magenta arrows) correlate well with ThioS-positive neuron populations, which strongly co-localize with (**c**) the DAPI nuclear counterstain. Normal hypodense neurons correlate well to ThioS-negative cells (azure arrows).

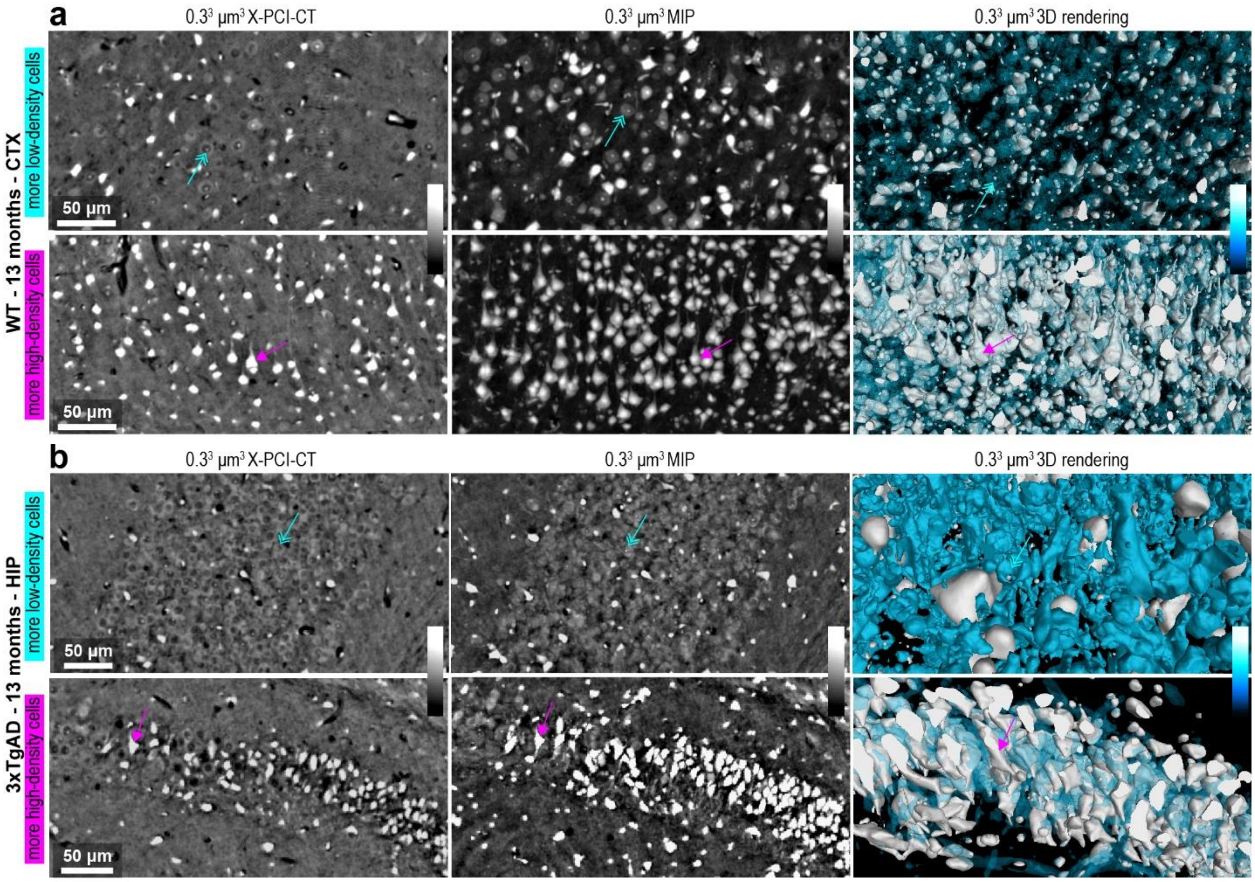

Suppl. Fig. 3 | X-PCI-CT-detected ICHD vs. normal cells. a-b, 0.3^3 µm^3 voxel X-PCI-CT maps, MIP maps, and 3D renderings of (**a**) CTX layers within the extracted brain of a 13 month old WT mouse and of (**b**) HIP layers within the extracted brain of a 13 month old 3xTgAD mouse. The presented layers highlight different levels of ICHD abundance (low to high ICHD prevalence over normal low-density neurons). The gray-scale calibration bars specify low-to-high (physical) tissue density. In the 3D renderings, the high gray-levels pertaining to ICHD-bearing cells are recolored as white, lower gray-levels pertaining to normal neuron somas as azure/blue (see Calibration Bar). Magenta and azure arrows point respectively to ICHD-bearing and normal pyramidal neurons.

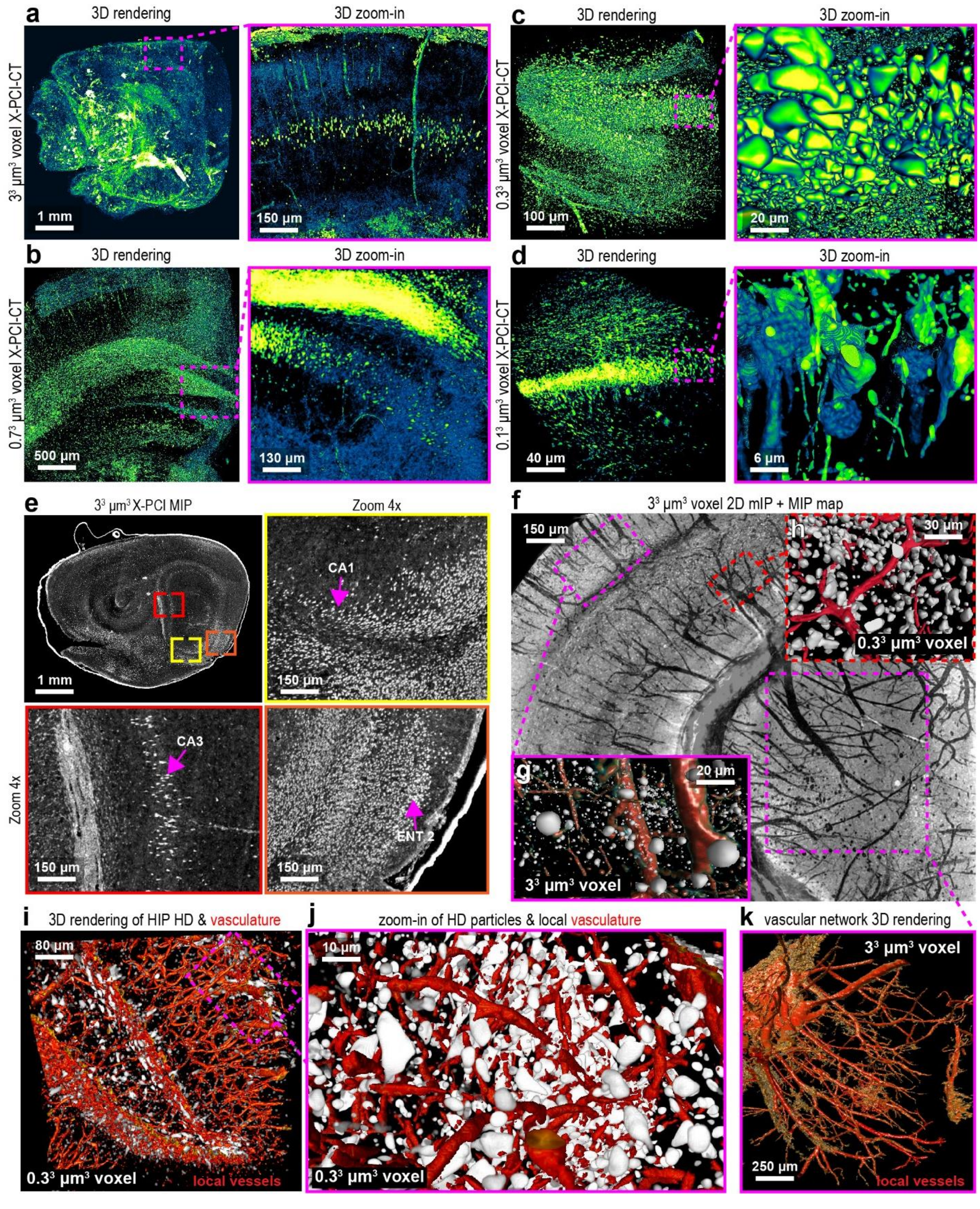

Suppl. Fig. 4 | Multiscale 3D X-PCI-CT. a-d, 3D renderings of (**a**) 3.0³ µm³, (**b**) 0.7³ µm³, (**c**) 0.3³ µm³ and (**d**) 0.1³ µm³ voxel X-PCI-CT data, after threshold-based gray-level segmentation and GFB LUT recoloring, showcasing the volumetric morphology of HD-particles at the organ- to intracellular level. **e,** 3.0³ µm³ voxel sagittal MIP (50 consecutive CT-slices), enhances HD neuronal populations, e.g. within CTX ENT 2, HIP CA1sp and CA3sp layers. **f,** coronal 0.3³ µm³ voxel MIP + minimum intensity projections (mIP, 200 consecutive CT-slices) highlighting hypodense

vasculature development alongside cellular HD deposition within the cerebrum in pseudo-3D. **g-k,** 3D rendering of HD particles (white) with cell-like morphology, alongside local vasculature (red), in (**g, k**) 3.0^3 μm^3 and (**h-j**) 0.3^3 μm^3 voxel X-PCI-CT datasets.

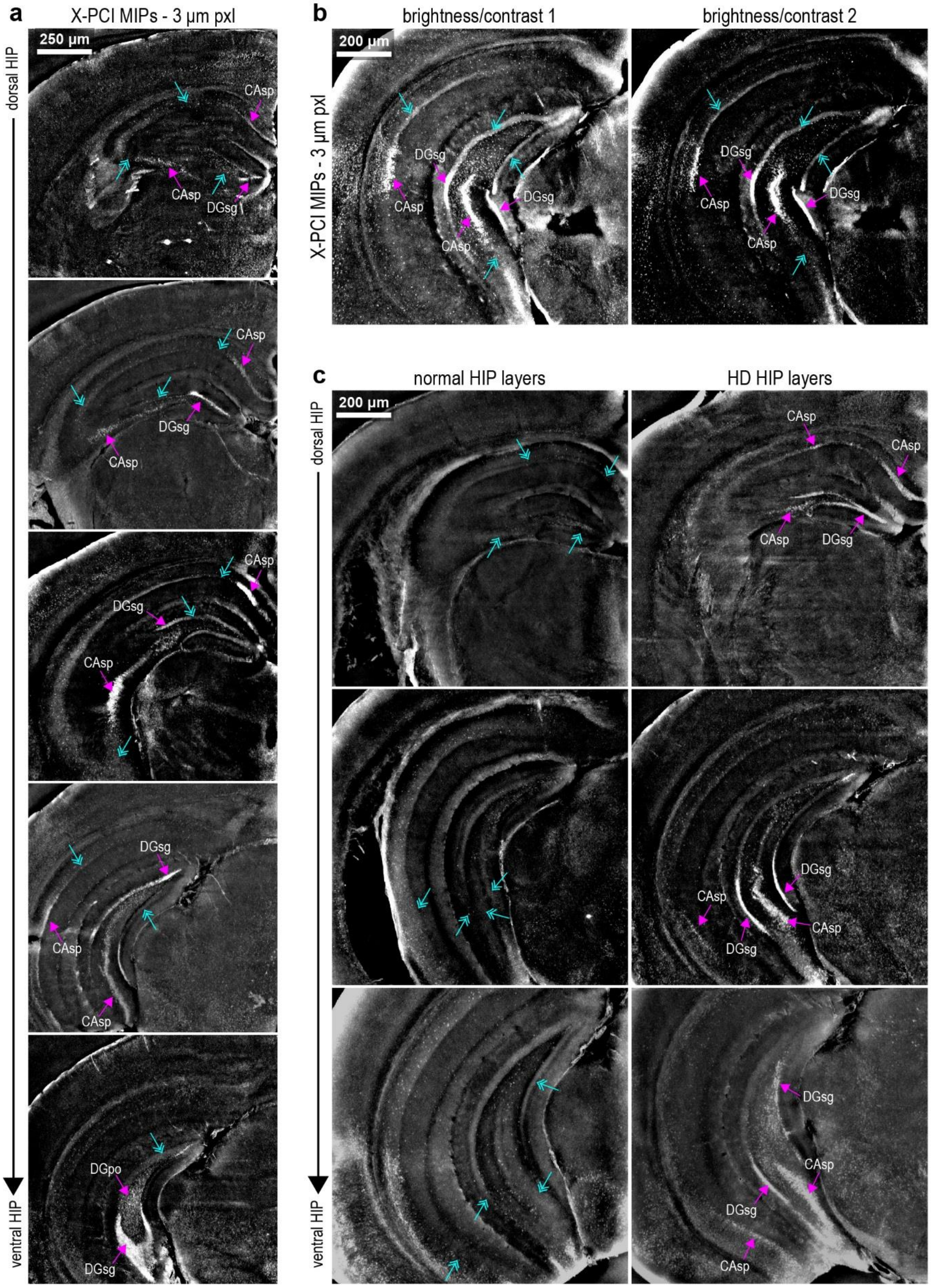

a
X-PCI MIPs - 3 µm pxl
250 µm
dorsal HIP
ventral HIP
CAsp
DGsg
DGpo
b
brightness/contrast 1
brightness/contrast 2
200 µm
X-PCI MIPs - 3 µm pxl
c
normal HIP layers
HD HIP layers
200 µm

Suppl. Fig. 5 | MIPs of the hippocampal formation highlight HD in CAsp and DGsg. a-c, 3.0^3 μm^3 voxel coronal MIPs of the HIP region, collected from several 13 month old mouse brains. **a,** dorsal to ventral HIP slices show layer-level HD cell-groups (magenta arrows) in CAsp and DGsg, compared to contiguous less-dense same-layer cell-groups (azure arrows). **b,** Different choices of gray-level windowing (brightness/contrast 1 vs. 2) increasingly enhance the visualization of local cell-layer HD (magenta arrows) in MIPs, compared to contiguous less-dense cell-groups (azure arrows). **c,** MIP-slices of HD-bearing HIP CAsp and DGsg cell-layers (magenta arrows), compared to same-level slices, bearing normal CAsp and DGsg cell-layers, that are relatively more HD-free (azure arrows).

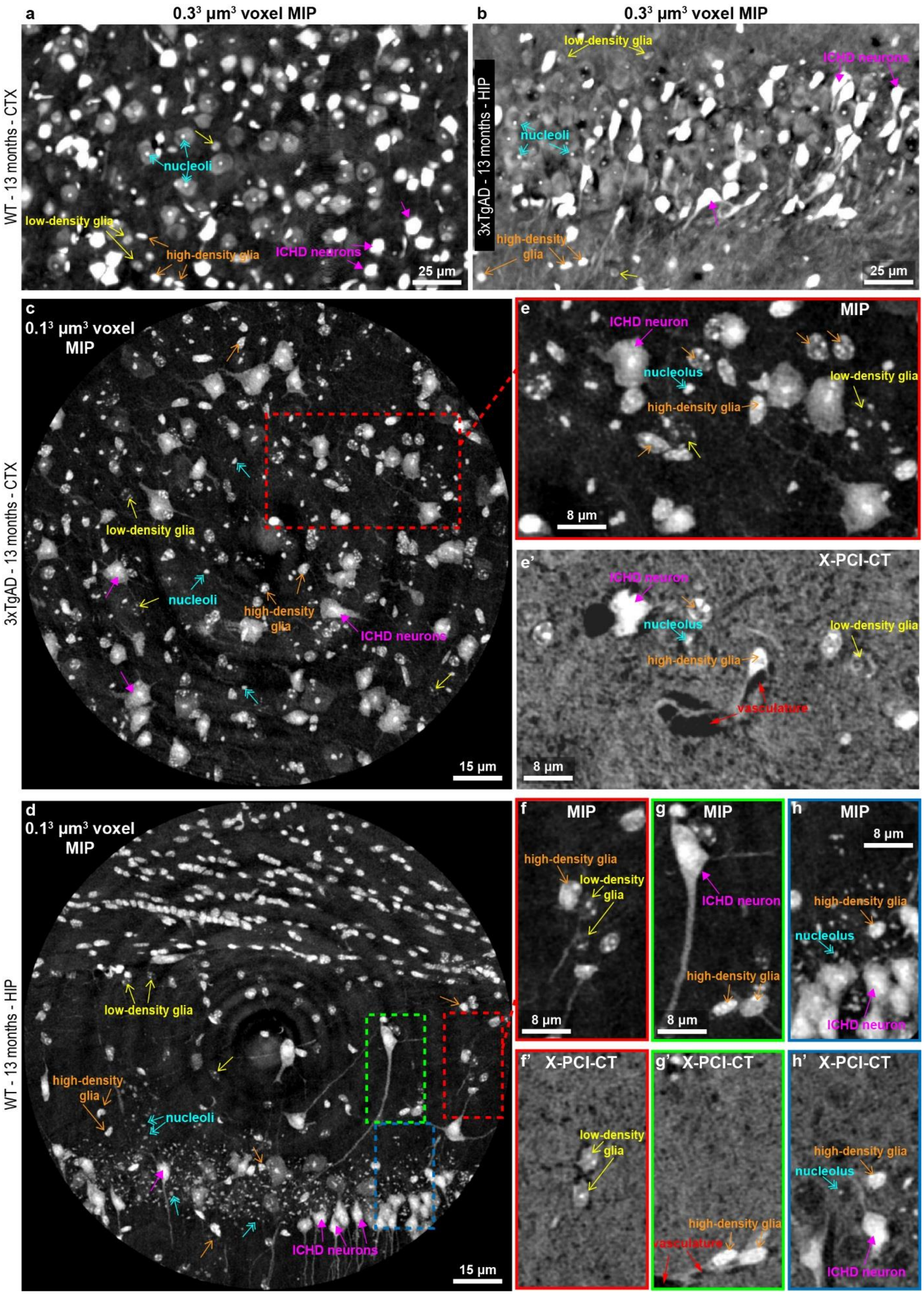

a
0.3³ µm³ voxel MIP
b
0.3³ µm³ voxel MIP
WT - 13 months - CTX
nucleoli
low-density glia
high-density glia
ICHD neurons
25 µm
3xTgAD - 13 months - HIP
low-density glia
ICHD neurons
nucleoli
high-density glia
25 µm
c
0.1³ µm³ voxel MIP
3xTgAD - 13 months - CTX
low-density glia
nucleoli
high-density glia
ICHD neurons
15 µm
e
MIP
ICHD neuron
nucleolus
high-density glia
low-density glia
8 µm
e'
X-PCI-CT
ICHD neuron
nucleolus
high-density glia
low-density glia
vasculature
8 µm
d
0.1³ µm³ voxel MIP
WT - 13 months - HIP
low-density glia
high-density glia
nucleoli
ICHD neurons
15 µm
f
MIP
high-density glia
low-density glia
8 µm
g
MIP
ICHD neuron
high-density glia
8 µm
h
MIP
8 µm
high-density glia
nucleolus
ICHD neuron
f'
X-PCI-CT
low-density glia
g'
X-PCI-CT
high-density glia
vasculature
h'
X-PCI-CT
high-density glia
nucleolus
ICHD neuron

Suppl. Fig. 6 | HD particles represent ICHD-bearing neurons, glia & normal nucleoli. a-d, (**a**,**b**) 0.3^3 μm^3 vs. (**c**,**d**) 0.1^3 μm^3 voxel-size cellular-level X-PCI-CT-based MIP data, showing morphology- and size-based annotations of visible HD particles in (**a**,**c**) CTX and (**b**,**d**) HIP layers. At least three main differently-sized neuronal and non-neuronal cellular populations are present: (1) strongly ICHD-bearing neurons (largest HD-particles, magenta arrows); (2) variedly-dense glial cells (intermediate size, yellow and brown arrows); (3) dense nucleoli at the center of smooth-circular hypodense nuclei in normal neurons (smallest HD-particles, azure arrows). Differences in both morphology and density within mid-sized non-neuronal cells, likely pertaining to different glial species, are visible: low-density glia presenting hypodense somas (yellow arrows, likely astrocytes), compared to higher-density glia presenting signs of abundant nuclear chromatin clumping (brown arrows, likely oligodendrocytes or microglia). **e** vs. **e'**, **f** vs. **f'**, **g** vs. **g'** and **h** vs. **h'** compare respectively MIPs vs. single-slice X-PCI-CTs of zoomed data from (**c-d**), containing HD-particles from all labelled cellular populations described in (**a-d**).

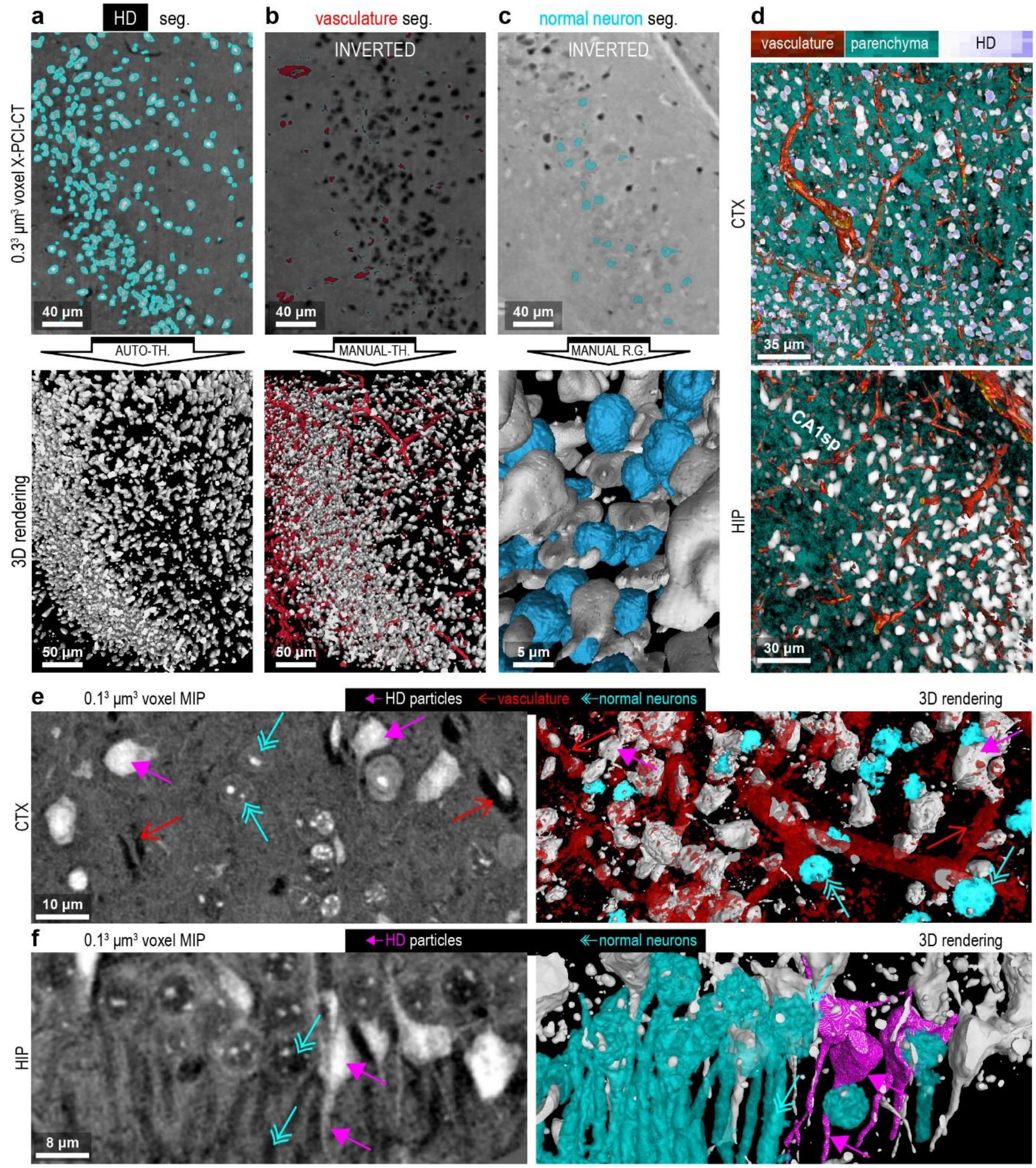

Suppl. Fig. 7 | Segmentation methods for X-PCI-CT-measured neuro-morphology. a-c, Representative (**a**) automated threshold-based (AUTO-TH), (**b**) manual threshold-based (MANUAL-TH) or (**c**) manual region-growing (MANUAL R.G.) segmentations (segmentation borders highlighted) obtained from (**a**) normal- or (**b-c**) inverted-contrast 0.3^3 μm^3 voxel X-PCI-CT images pertaining to HIP CA cells. Below the 3D rendering of (**a**) ICHD-bearing (white in 3D), (**b**) + vasculature (red in 3D) or (**c**) + normal cells (azure in 3D), obtained after each type of segmentation. **d,** 3D rendering of 0.3^3 μm^3 X-PCI-CT data visualizing CTX, HIP CA and HIP DG regions, including individual ICHD-bearing cells alongside local micro-vasculature, obtained after application of the segmentation methodology described in (**a-c**). High gray-levels pertaining to ICHD-bearing cells are visualized in white/magenta, lower gray-levels pertaining to normal brain parenchyma in azure, and the very low gray-levels pertaining to vascular features in red/yellow (See Calibration Bar). **e-f,** 2D 0.1^3 μm^3 voxel MIPs vs. 3D renderings of ICHD-bearing cells (white in **e**, white or magenta in

f), vasculature (red), and normal cells (azure), within volumes containing CTX or HIP cell-groups, after application of the segmentation methodology described in (**a-c**). Magenta, red and azure arrows point to respectively ICHD-bearing neurons, vasculature and normal neurons. In (**f**), arrows point to the same cells and cellular processes rendered in pseudo-3D (left, MIP map) vs. 3D (right, 3D rendering).

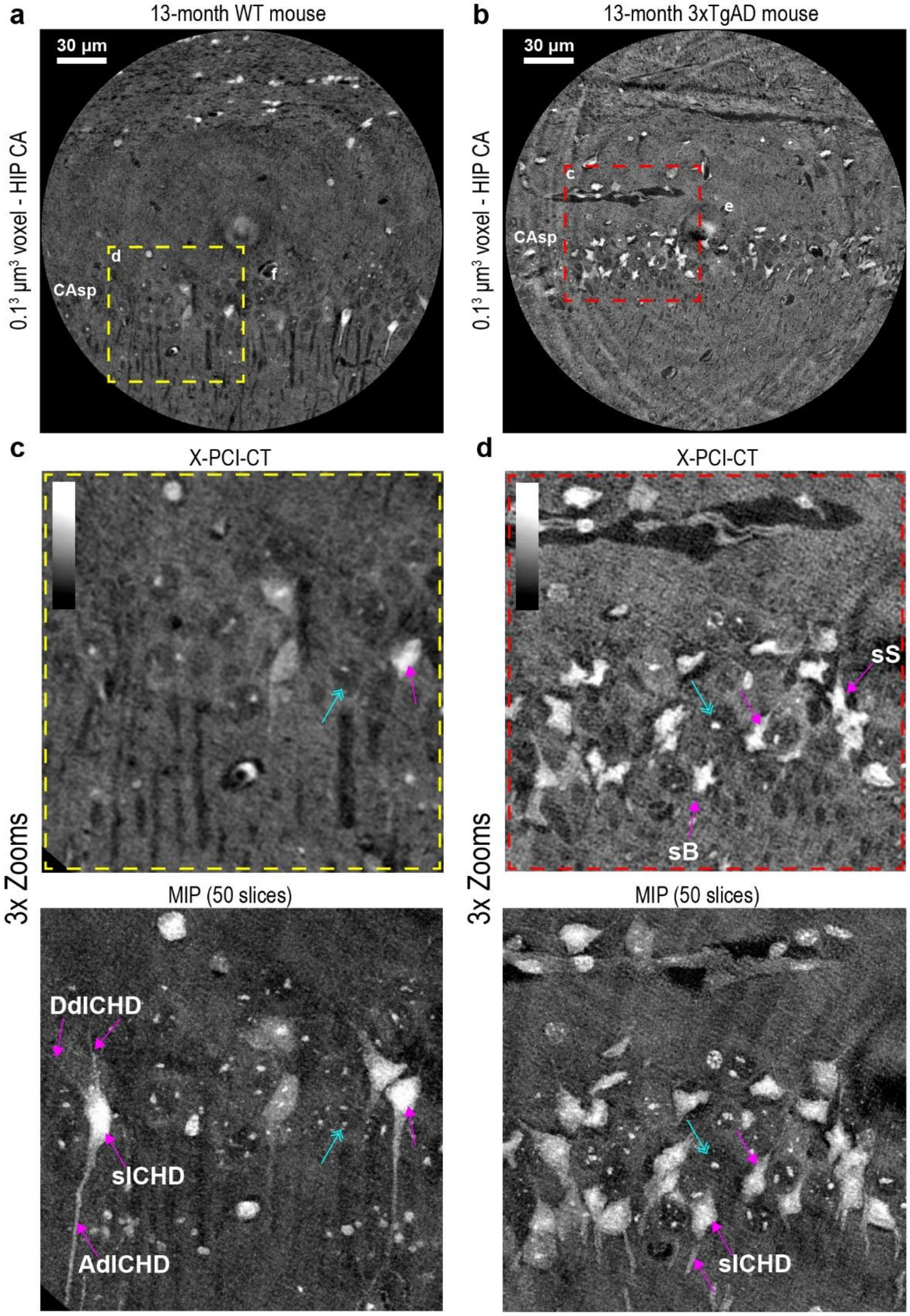

Suppl. Fig. 8 | Intracellular nano-anatomy of ICHD-bearing neurons in WT vs. 3xTgAD mice. a-b, Label-free 0.1^3 μm^3 voxel X-PCI-CT visualization of HIP CAsp pyramidal neurons in the brain of (**a**) a 13 month old WT and (**b**) a 13 month old 3xTgAD mice. **c-d,** 3x Zooms of X-PCI-CTs in (**a,b**) and their MIPs (50 contiguous CT slices) visualize local intracellular nano-anatomy. Individual ICHD-bearing neurons (magenta arrows), compared to normal relatively more hypodense cells (azure arrows), are visible in both aged WT (**c**) and aged 3xTgAD mice (**d**). In WT samples (**c**) ICHD-bearing somas appear well-rounded (sICHD), and both axonal (AdICHD) and dendritic (DdICHD) ICHD depositions are visible, recognizable as hyper-intense branch-like extensions departing the HD somas. In 3xTgAD samples (**d**) ICHD-bearing somas present pathological cyto-morphology, signs of cell blebbing (sB) and shrinkage (sS), typical features of cellular neurodegeneration.

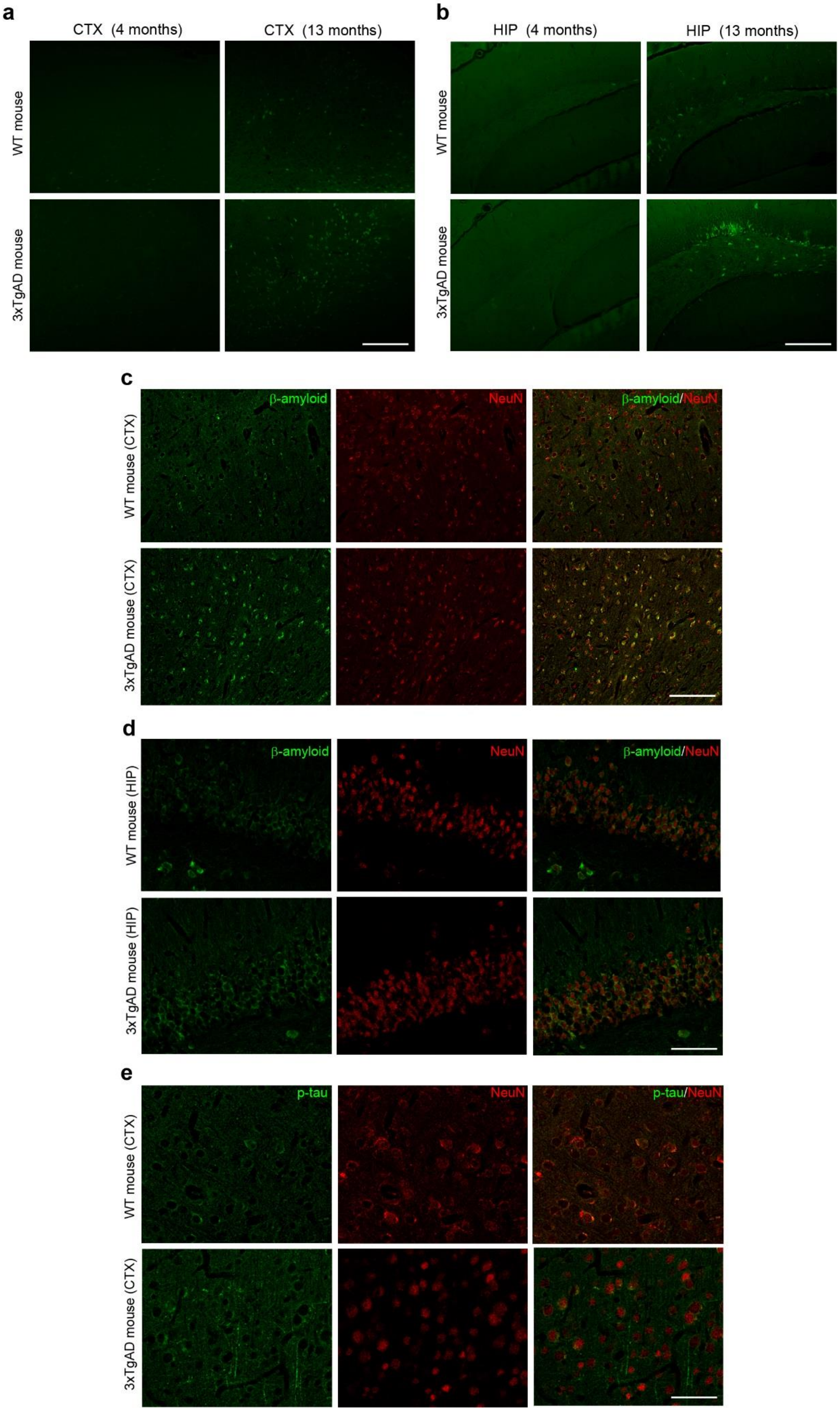
a
CTX (4 months)
CTX (13 months)
WT mouse
3xTgAD mouse
b
HIP (4 months)
HIP (13 months)
WT mouse
3xTgAD mouse
c
β-amyloid
NeuN
β-amyloid/NeuN
WT mouse (CTX)
3xTgAD mouse (CTX)
d
β-amyloid
NeuN
β-amyloid/NeuN
WT mouse (HIP)
3xTgAD mouse (HIP)
e
p-tau
NeuN
p-tau/NeuN
WT mouse (CTX)
3xTgAD mouse (CTX)

Suppl. Figure 9 | Thioflavin-S staining matches immunoreactivity for Aβ and p-tau in the cerebral cortex and hippocampus. a,b Thioflavin S fluorescence microscopy data of cerebral cortex and hippocampus of 13 month old WT and 3xTgAD mice collected from contralateral brains hemispheres. In (**c-d**), representative cerebral cortex (**c**) and hippocampus (**d**) IHC data collected from contralateral-hemisphere doubly stained for Aβ/NeuN (anti-Aβ 4G8 fluorescence in green, anti-NeuN in red) from 13 month old WT and 3xTgAD mice, at different magnifications. **e**, representative cerebral cortex IHC data collected from contralateral-hemisphere doubly stained for p-tau/NeuN (anti-p-tau pSer404 fluorescence in green, anti-NeuN in red) from 13 month old WT and 3xTgAD mice. These data indicate that Aβ and p-tau immunoreactivity in 3xTgAD mice correlates well with the detected ThioS-positive cell-shaped clusters (**a,b**), which in turn match well the dense particles detected by X-PCI-CT (**Fig. 2-5**), suggesting that the intracellular deposits observed by X-PCI-CT in 3xTgAD are very likely composed of Aβ and p-tau. Scale bars: 100 µm in **a**,**b**, 50 µm in **c** and 25 µm in **d,e**.

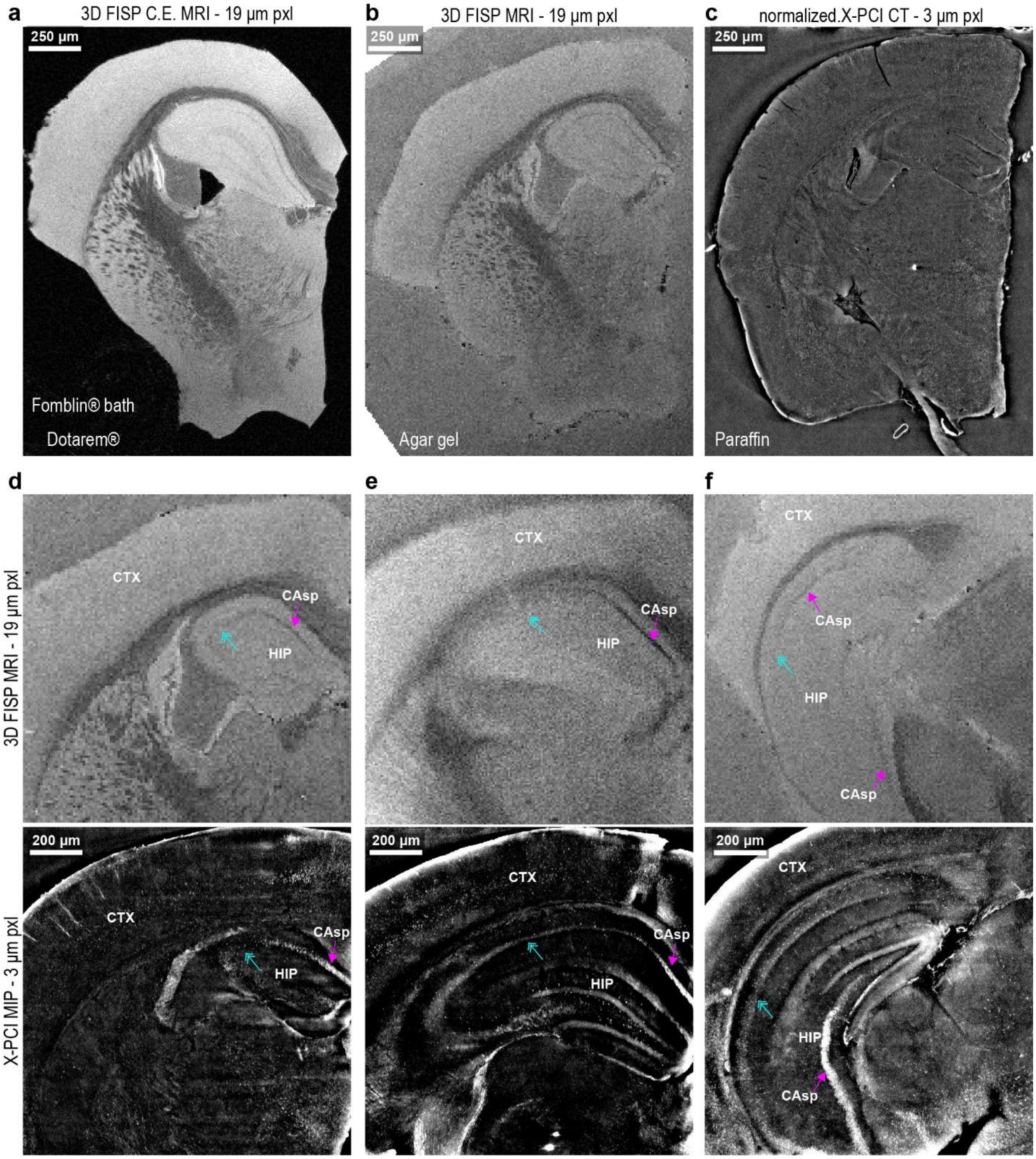

Suppl. Fig. 10 | X-PCI-CT vs. 9.4 T MRI. a-b, Comparative imaging of aged 3xTgAD half brain samples, using either (**a-b**) high-field 9.4 Tesla (T) volumetric (19^3 µm^3 voxel) MRI, measured after placement of the excised brain sample in either (**a**) a contrast-enhancing Dotarem® bath (Gadolinium-based MRI contrast agent) and then in an artifact-reducing Fomblin® bath or (**b**) only within Agarose gel, or (**c**) 3.0^3 µm^3 voxel X-PCI-CT, measured with the excised brain sample embedded in paraffin. **d-f,** Local dorsal (**d**) to ventral (**f**) CTX and HIP zooms of MRI data, measured using the same sequence and sample preparation as in (**b**), vs. X-PCI-CT MIP data of comparable CTX/HIP layers, measured as in (**c**). In CTX layers, little sign of aberrant hypo-density is visible at this resolution via MRI, compared to some sparse CTX ICHD visible in X-PCI-based MIPs. In HIP layers, instead, layer-like hypo-density within cell-groups

in the key AD-linked CAsp layer (magenta arrows), compared to contiguous less-hypodense same-layer cells (azure arrows), are visible in the MRI data. Similar patterns of CAsp layer morphology, featuring layer-like ICHD (magenta arrows), compared to contiguous more-hypodense CAsp layer areas populated by normal pyramidal cells (azure arrows), are visible in the X-PCI-based MIPs.

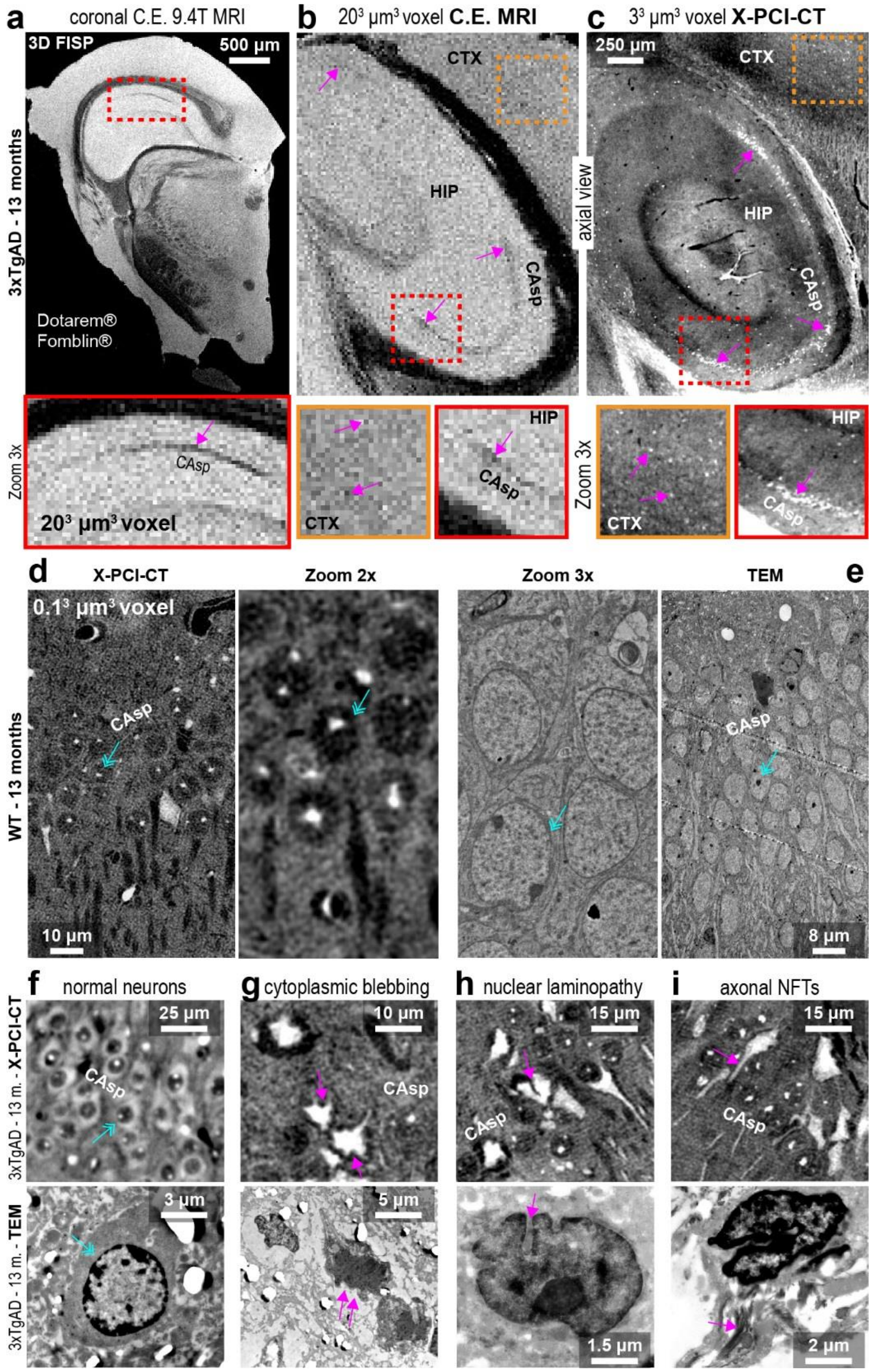

Suppl. Figure 11 | X-PCI-CT vs. MRI and TEM. a-c, 20^3 μm^3 voxel contrast-enhanced (C.E.) coronal (**a**) and axial (**b**) 9.4 T postmortem MRI vs. (**c**) axial 3^3 μm^3 voxel postmortem X-PCI-CT of comparable CTX and HIP layers within a

3xTgAD half brain sample. Intra-layer hypo-density (magenta arrows) is visible via MRI in HIP CAsp (see Zooms 3x) and matches layer-like HD-signal in the X-PCI-CT of HIP CAsp. Sparse CTX ICHD-bearing cells are well-resolved via X-PCI-CT, only barely visible via MRI. In the MRI acquisitions, Dotarem® was used as contrast-agent, Fomblin® for artifact reduction. **d-e,** 0.1^3 µm^3 voxel (**d**) X-PCI-CT vs. (**e**) TEM visualizations (and Zooms) of normal-morphology HIP CAsp pyramidal neurons (azure arrows) within comparable WT brain tissues. **f-i,** cellular-level X-PCI-CT vs. TEM brain-tissue data from one 13 month old 3xTgAD mouse. Both techniques visualize (**f**) normal neurons (azure arrows) with spherical cytoplasms, intact nuclear envelopes, and low-density axons; ICHD-bearing cells (magenta arrows) morphologically match TEM-based visualizations of likely-neurodegenerating neurons, bearing signs of cytoplasmic blebbing (**g**), nuclear-envelope laminopathy (**h**), and abnormal hyperdense axonal cyto-architecture (probable NFTs) (**i**).

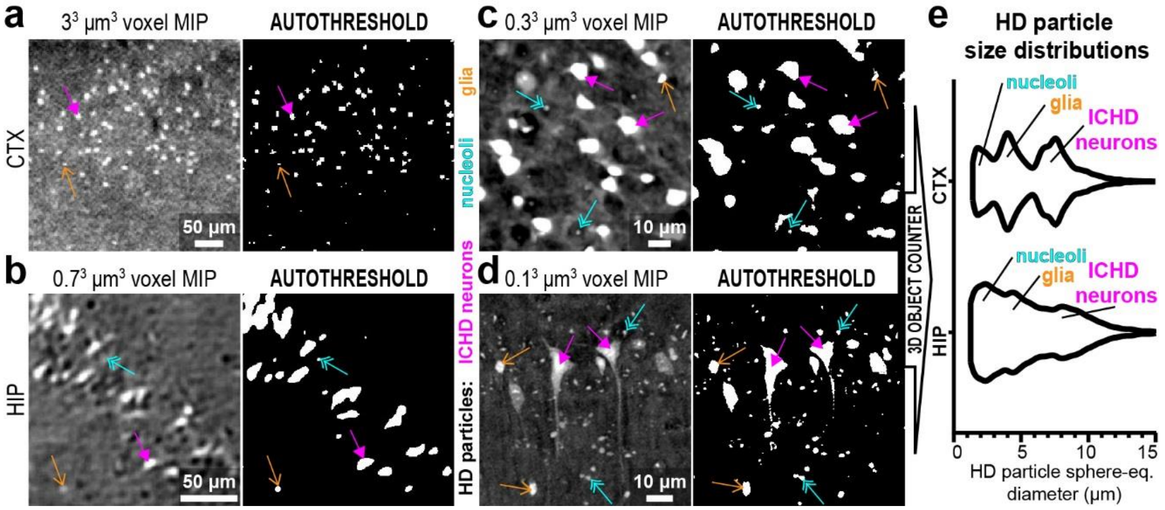

Suppl. Figure 12 | 3D Segmentation & quantification of HD particles. a-d, Representative results of systematic auto-threshold segmentations of HD particles in (**a**) 3.0^3 µm^3, (**b**) 0.7^3 µm^3, (**c**) 0.3^3 µm^3 and (**d**) 0.1^3 µm^3 CTX and HIP X-PCI-CT volumes of interest (VOI). Segmented HD-particles in (**b-d**) correspond to three cell-populations: ICHD-bearing neurons (largest particles, magenta arrows), HD glia (intermediate-size, brown arrows) and nucleoli of normal neurons (smallest, azure arrows). Nucleoli are not resolved in (**a**). **e,** Computation of HD-particle volumes within the auto-segmented 3D-masks (**a-d**), using the ImageJ 3D Object Counter plug-in, resulted in the extraction of HD particle size distributions (size expressed as equivalent-sphere diameter). Distributions exhibit tri-modal shapes, in good agreement with a three-population HD particle model.

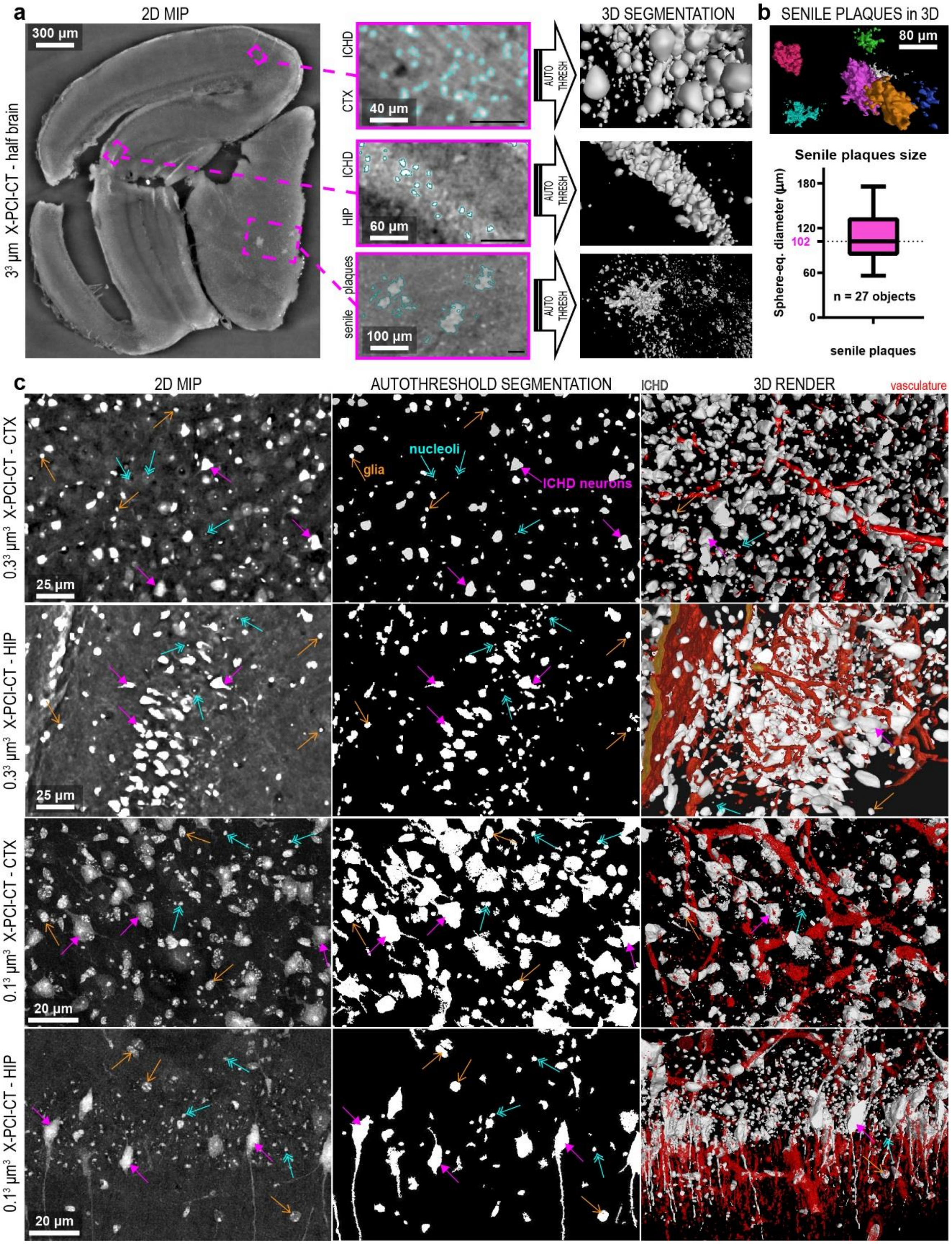

Suppl. Fig. 13 | Segmentation & quantification of plaque-like HD vs. auto-segmentation of ICHD. a, preliminary auto-threshold segmentation of 3.0^3 μm^3 voxel X-PCI-CT half brain data, with extraction of 1. ~10 µm-big ICHD with particle-like morphology within CTX and HIP layers, and 2. of macroscopic ~ 100 µm large HD clusters of plaque-like morphology. Black scale-bars measure 50 µm. **b,** One-off quantification of individual macroscopic plaque-like HD clusters (n=27) within three 13 month old mice, including 1 WT and 2 3xTgAD mice, after manual region-growing

segmentation. Data in the box-plot are minimum, maximum, median, first and third quartiles. **c,** Representative panels of auto-threshold segmentations and 3D renderings of HD particles within HIP and CTX layers, using 0.3^3 μm^3 and 0.1^3 μm^3 voxel X-PCI-CT data and Max-Entropy-based auto-threshold segmentation methodology. 3D renderings visualize HD particles (white) and local hypodense vasculature (red), instead extracted with a manual threshold-based segmentation. HD particles pertain to three different cellular populations, (1) larger ICHD-bearing cells (magenta arrows), (2) intermediate-size HD glial cells (brown arrows) and (3) small dense nucleoli in the nucleus of normal hypodense neurons (azure arrows).

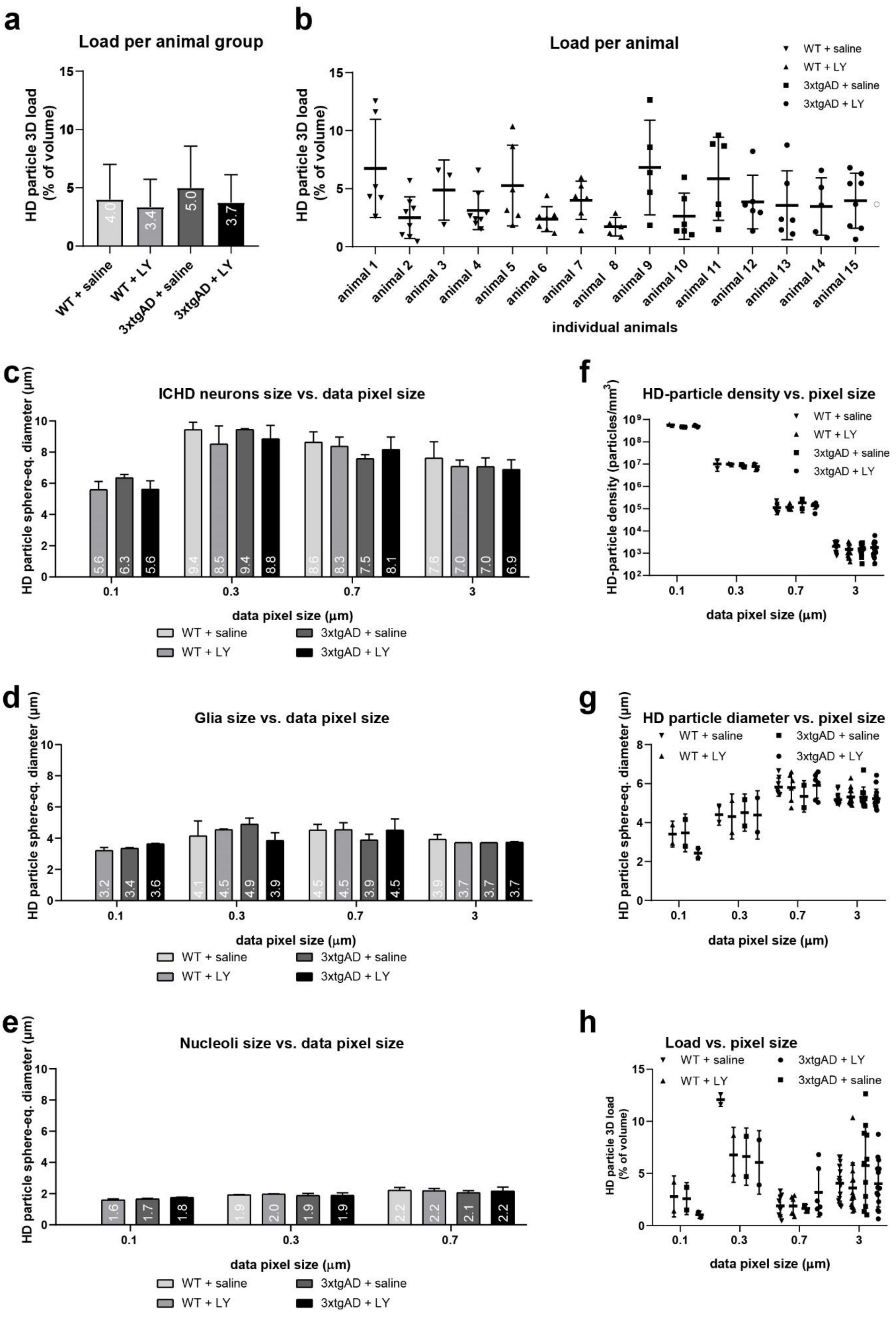
a
Load per animal group
HD particle 3D load (% of volume)
0
5
10
15
4.0
3.4
5.0
3.7
WT + saline
WT + LY
3xtgAD + saline
3xtgAD + LY
b
Load per animal
WT + saline
WT + LY
3xtgAD + saline
3xtgAD + LY
HD particle 3D load (% of volume)
animal 1
animal 2
animal 3
animal 4
animal 5
animal 6
animal 7
animal 8
animal 9
animal 10
animal 11
animal 12
animal 13
animal 14
animal 15
individual animals
c
ICHD neurons size vs. data pixel size
HD particle sphere-eq. diameter (µm)
5.6
6.3
5.6
9.4
8.5
9.4
8.8
8.6
8.3
7.5
8.1
7.6
7.0
7.0
6.9
0.1
0.3
0.7
3
data pixel size (µm)
WT + saline
WT + LY
3xtgAD + saline
3xtgAD + LY
f
HD-particle density vs. pixel size
HD-particle density (particles/mm³)
WT + saline
WT + LY
3xtgAD + saline
3xtgAD + LY
data pixel size (µm)
d
Glia size vs. data pixel size
HD particle sphere-eq. diameter (µm)
3.2
3.4
3.6
4.1
4.5
4.9
3.9
4.5
4.5
3.9
4.5
3.9
3.7
3.7
3.7
data pixel size (µm)
WT + saline
WT + LY
3xtgAD + saline
3xtgAD + LY
g
HD particle diameter vs. pixel size
WT + saline
WT + LY
3xtgAD + saline
3xtgAD + LY
HD particle sphere-eq. diameter (µm)
data pixel size (µm)
e
Nucleoli size vs. data pixel size
HD particle sphere-eq. diameter (µm)
1.6
1.7
1.8
1.9
2.0
1.9
1.9
2.2
2.2
2.1
2.2
data pixel size (µm)
WT + saline
WT + LY
3xtgAD + saline
3xtgAD + LY
h
Load vs. pixel size
WT + saline
WT + LY
3xtgAD + LY
3xtgAD + saline
HD particle 3D load (% of volume)
data pixel size (µm)

Suppl. Fig. 14 | More on the proof-of-principle drug-test with the group II metabotropic glutamate receptor agonist LY379268. a, group mean HD-particle 3D load (bar-graph shows group mean total HD-particle volume as % of total sample-volume ± SD) in all four experimental groups, using all extracted sample-volumes independently of tissue area of origin. No significant difference in load between animal groups could be calculated by way of ANOVA testing (test details as in **Fig. 8**). **b,** HD-particle 3D load (graph shows individual sample-volume values and mean total-HD-particle volume % of total sample-volume ± SD) for each animal used in the study. Note the high load variability within individual animals. **c-e**, comparison of group mean particle size (bar-graphs show group mean HD-particle size ± SD) for each of the three HD-particle populations, i.e. (**c**) ICHD neurons, (**d**) microglia and (**e**) normal neuron nucleoli, with sample-volumes further subdivided by effective pixel size of the sample data. No significant difference in mean particle size between animal groups, or for different pixel sizes, could be calculated by way of ANOVA testing (test details as in **Fig. 8**). **f-h,** analysis of (**f**) HD-particle density (graph shows individual sample-volume values and mean HD-particle density ± SD), (**g**) HD particle sizes (equivalent-sphere diameters, data as individual sample-volume values and group mean HD-particle eq.-sphere diameter ± SD) and (**h**) HD-particle 3D load (graph shows individual sample-volume values and mean total-HD-particle volume % of total sample-volume ± SD) in the four animal groups, further sub-divided by the effective pixel size of the sample-volume data. Note how smaller imaging-system pixel sizes portray higher densities (**f**), since they detect more, though smaller on average (see (**g**)), HD particles.

SUPPLEMENTARY VIDEOS (Legends):

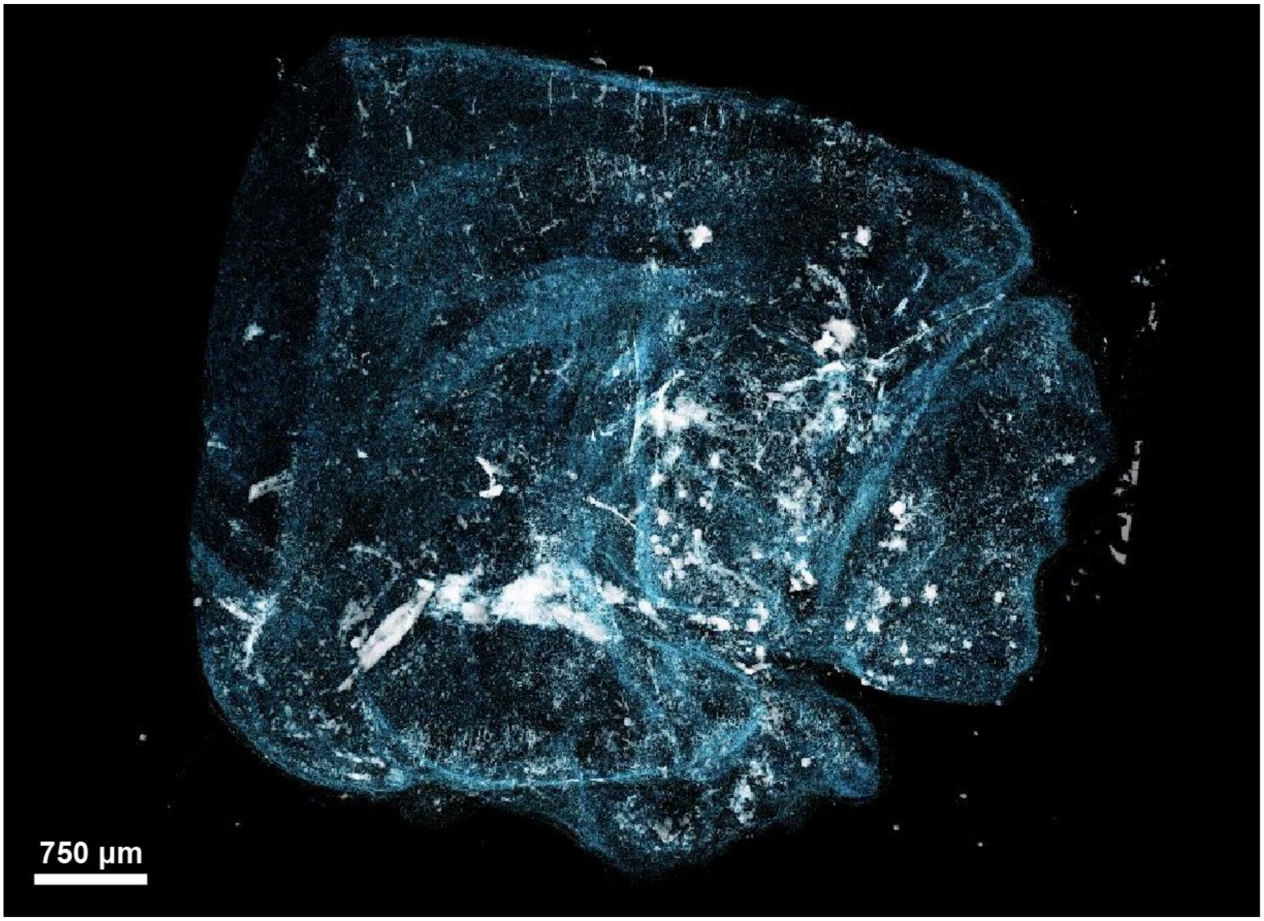

Suppl. Video 1 | 3D rendering of a 3.0^3 μm^3 voxel X-PCI-CT dataset from a 13 month old WT mouse. A half murine brain is visualized.

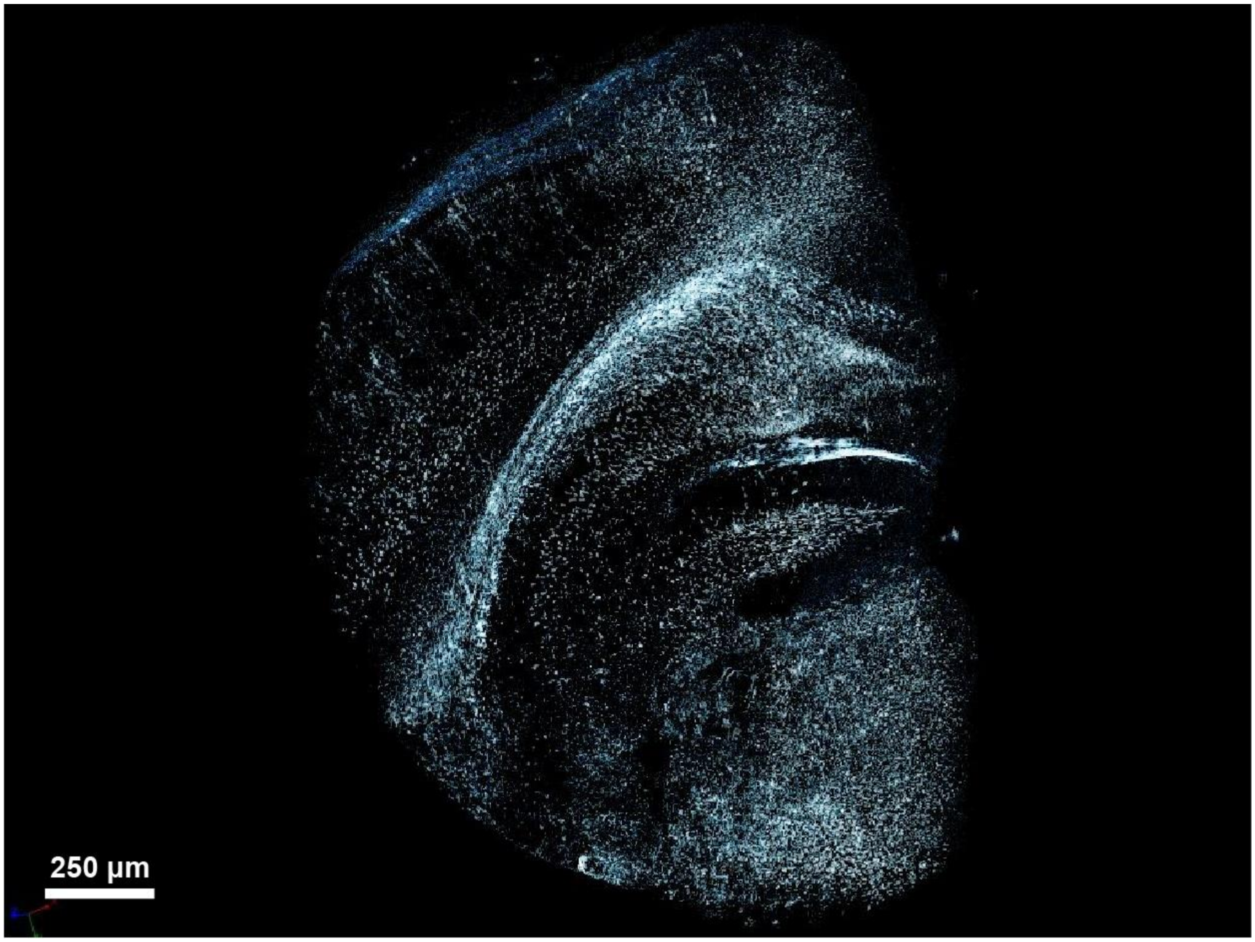

Suppl. Video 2 | 3D rendering of a 0.7^3 μm^3 voxel X-PCI-CT dataset from a 13 month old WT mouse. Cortical and hippocampal areas are visualized.

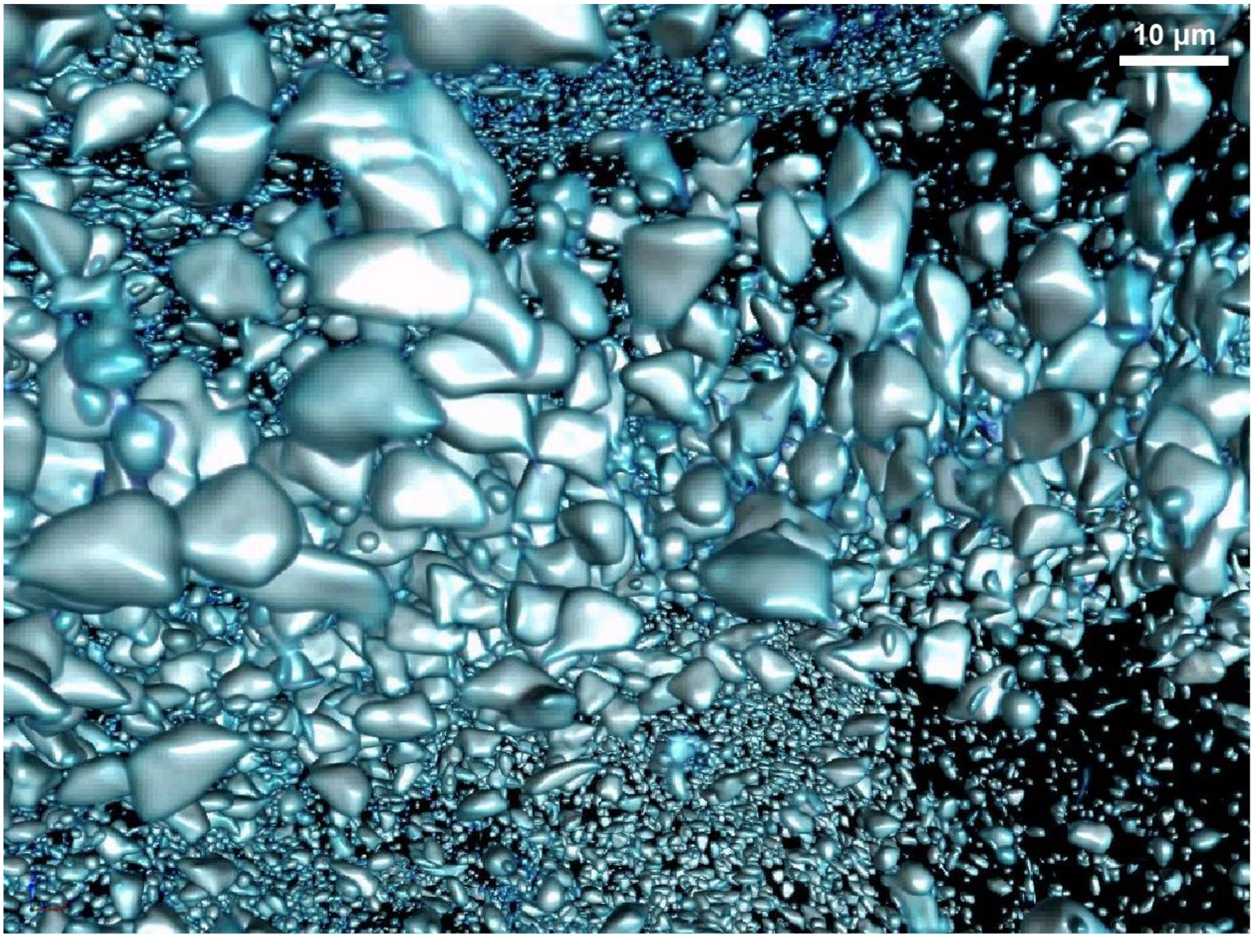

Suppl. Video 3 | 3D rendering of a 0.3^3 μm^3 voxel X-PCI-CT dataset from a 13 month old WT mouse. Hippocampal layers are visualized.

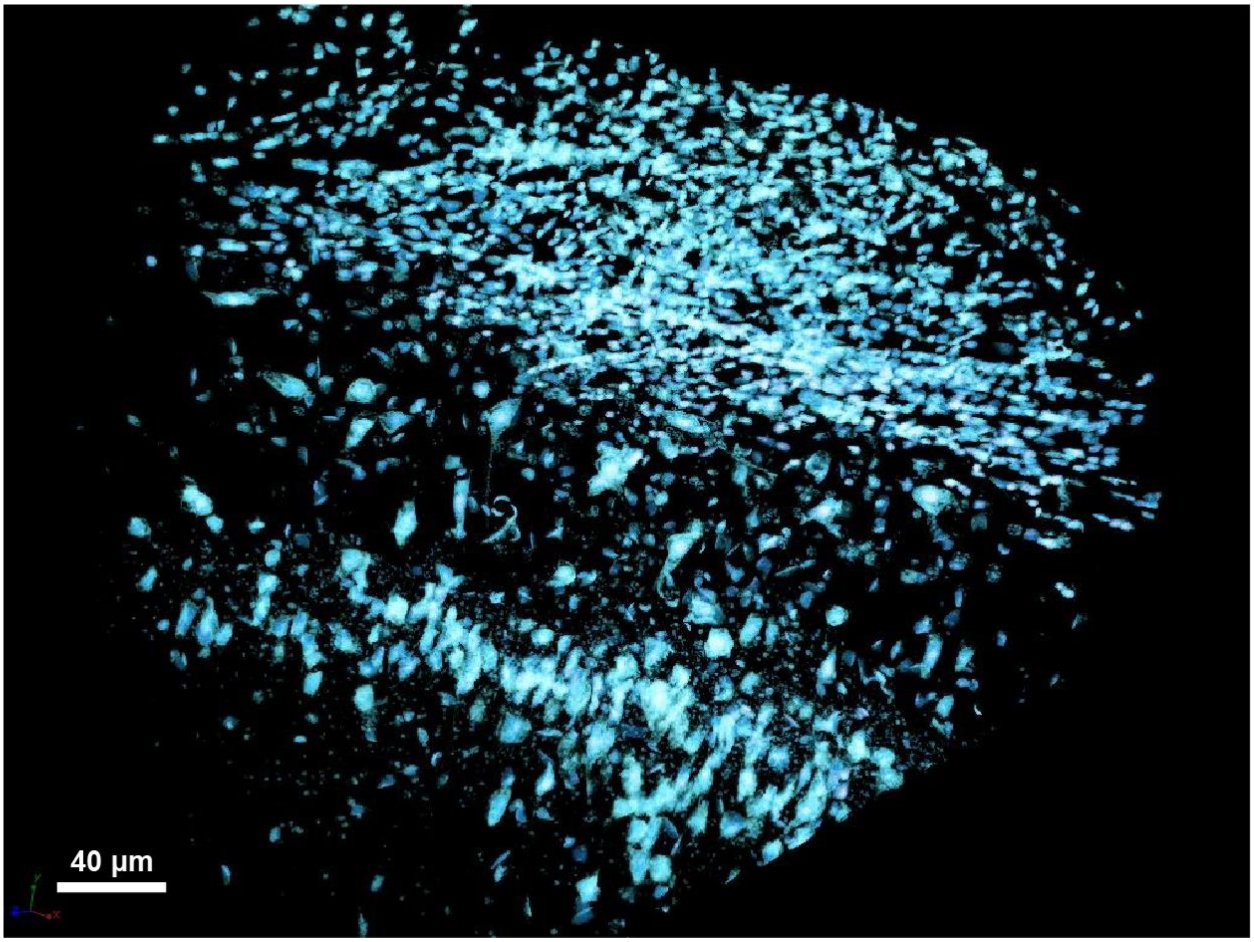

Suppl. Video 4 | 3D rendering of a 0.1^3 μm^3 voxel X-PCI-CT dataset from a 13 month old WT mouse. Hippocampal cells are visualized.

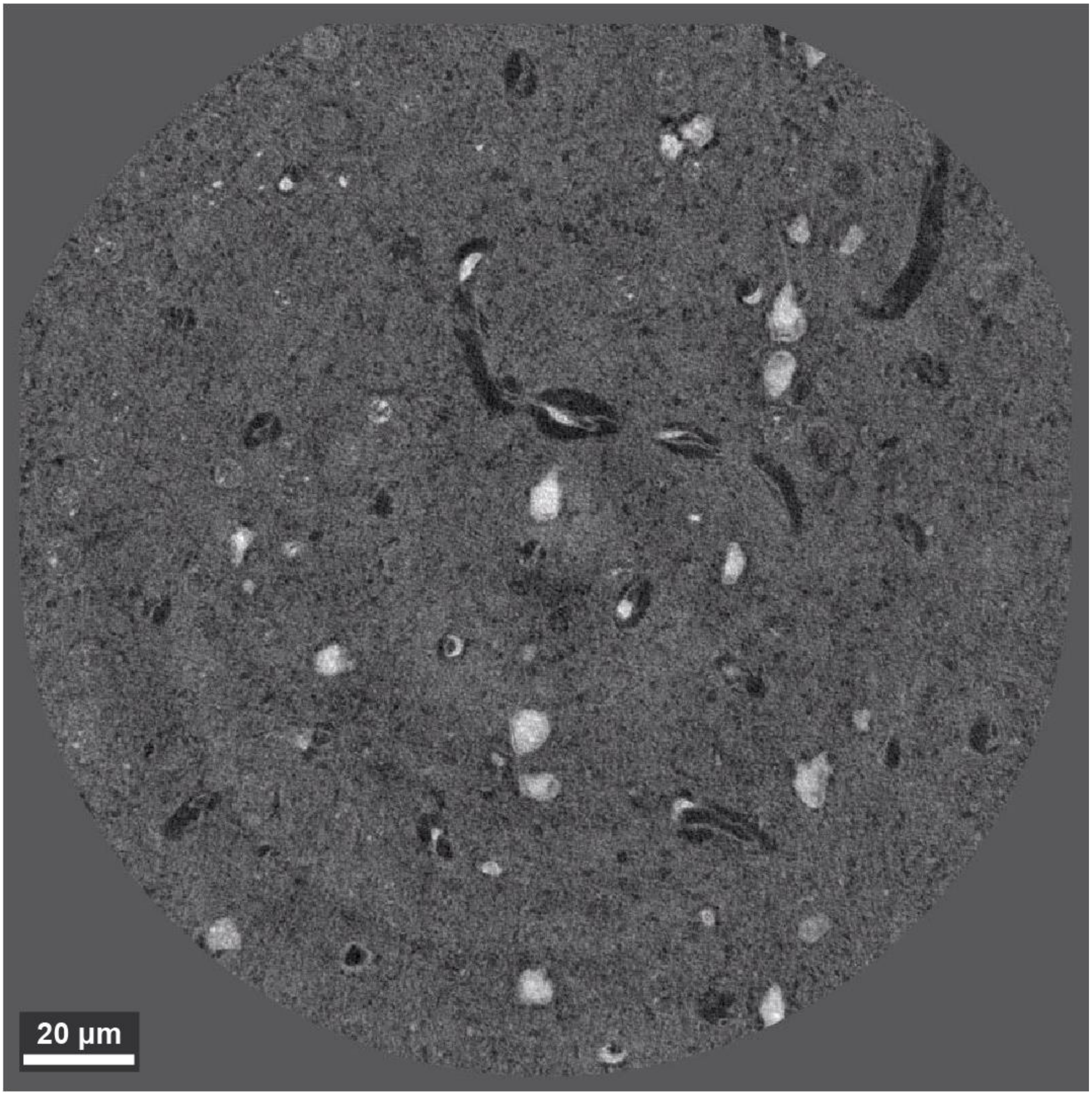

Suppl. Video 5 | CT image stack of a 0.1^3 μm^3 voxel X-PCI-CT dataset from a 13 month old 3xtgAD mouse. A dorsal cortical layer is visualized.

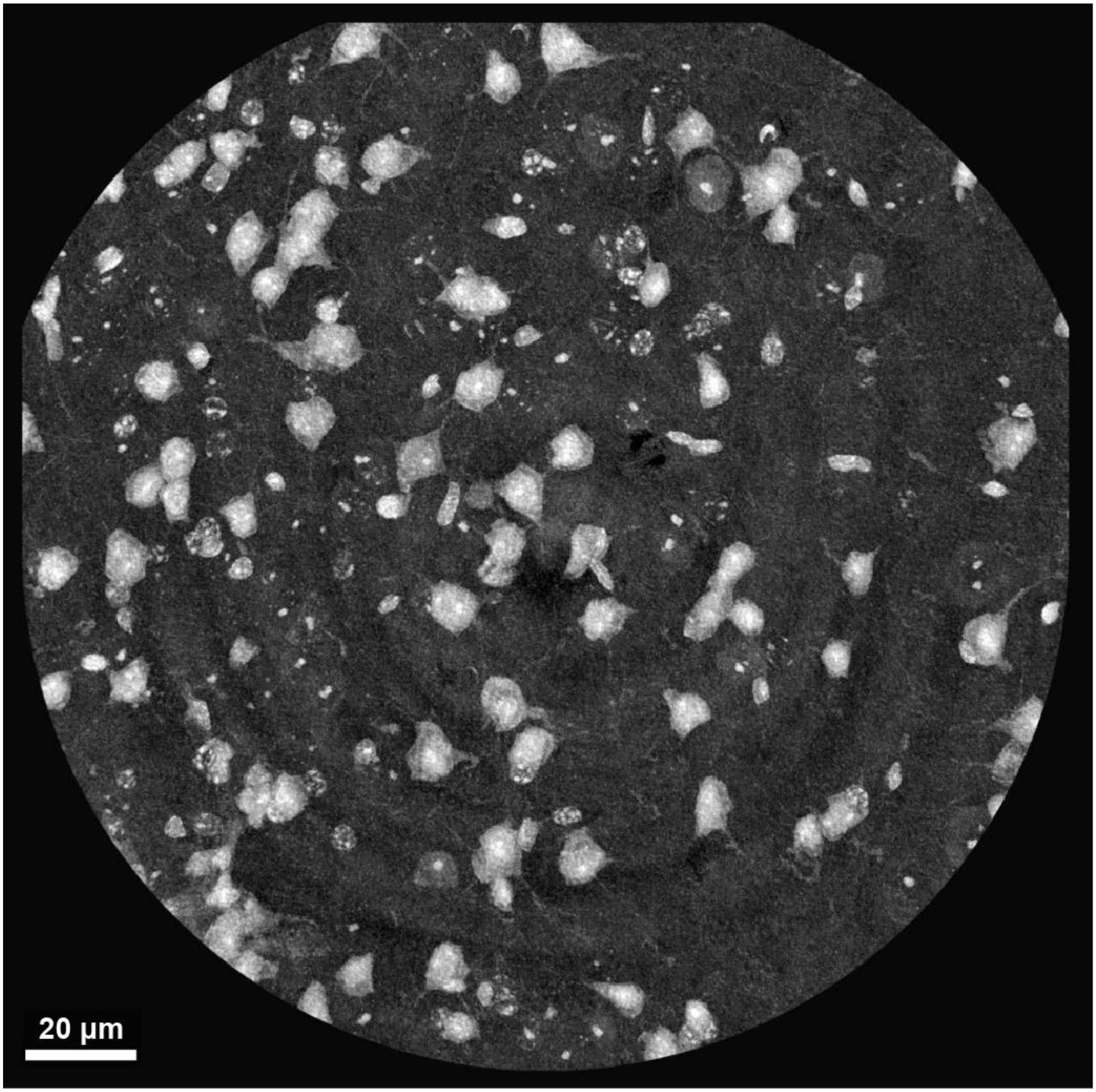

Suppl. Video 6 | Stack of consecutive 0.1^3 μm^3 voxel X-PCI-CT cortical MIPs, computed with the dataset in Suppl. Video 5.

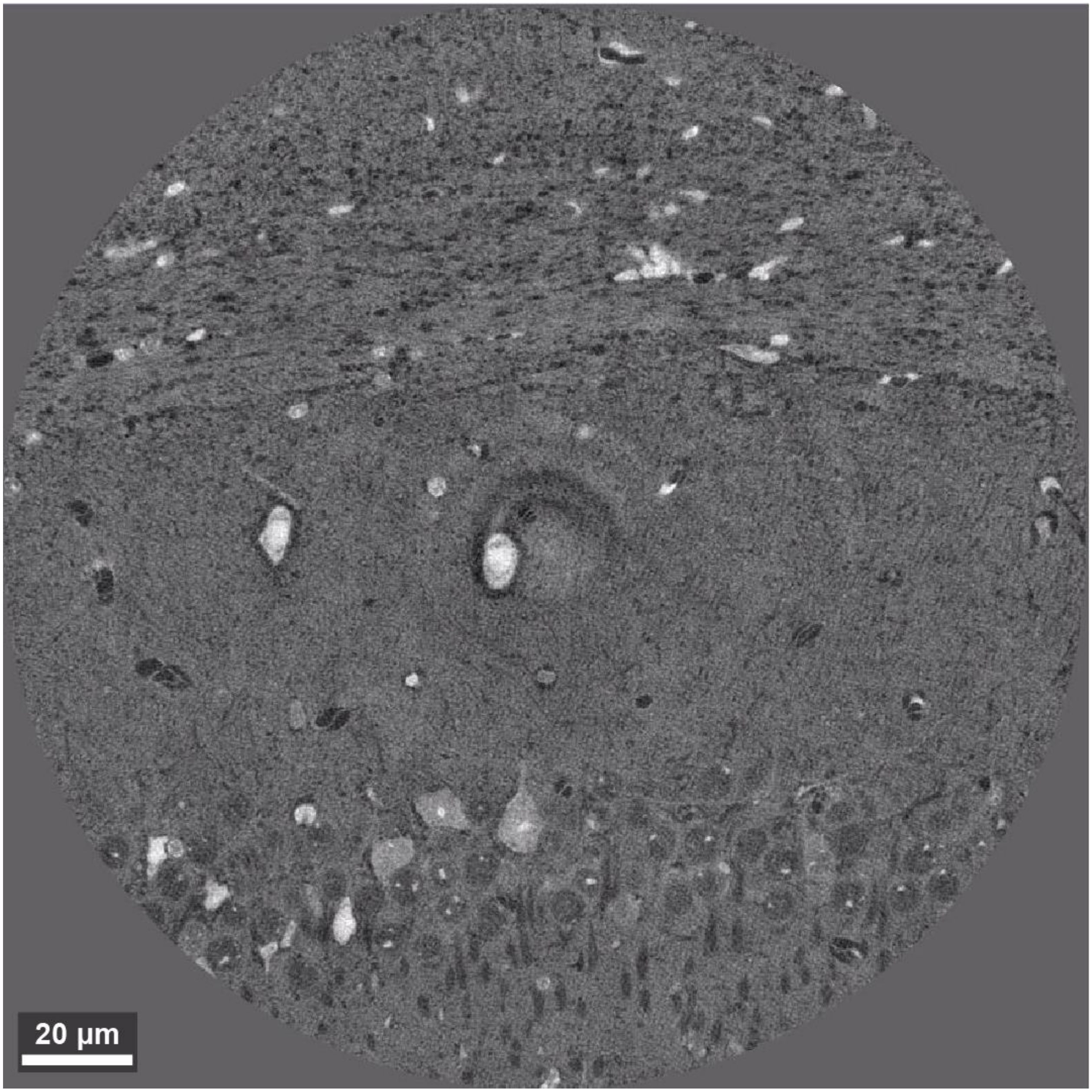

Suppl. Video 7 | CT image stack of a 0.1^3 μm^3 voxel X-PCI-CT dataset from a 13 month old WT mouse. A hippocampal CAsp layer is visualized.

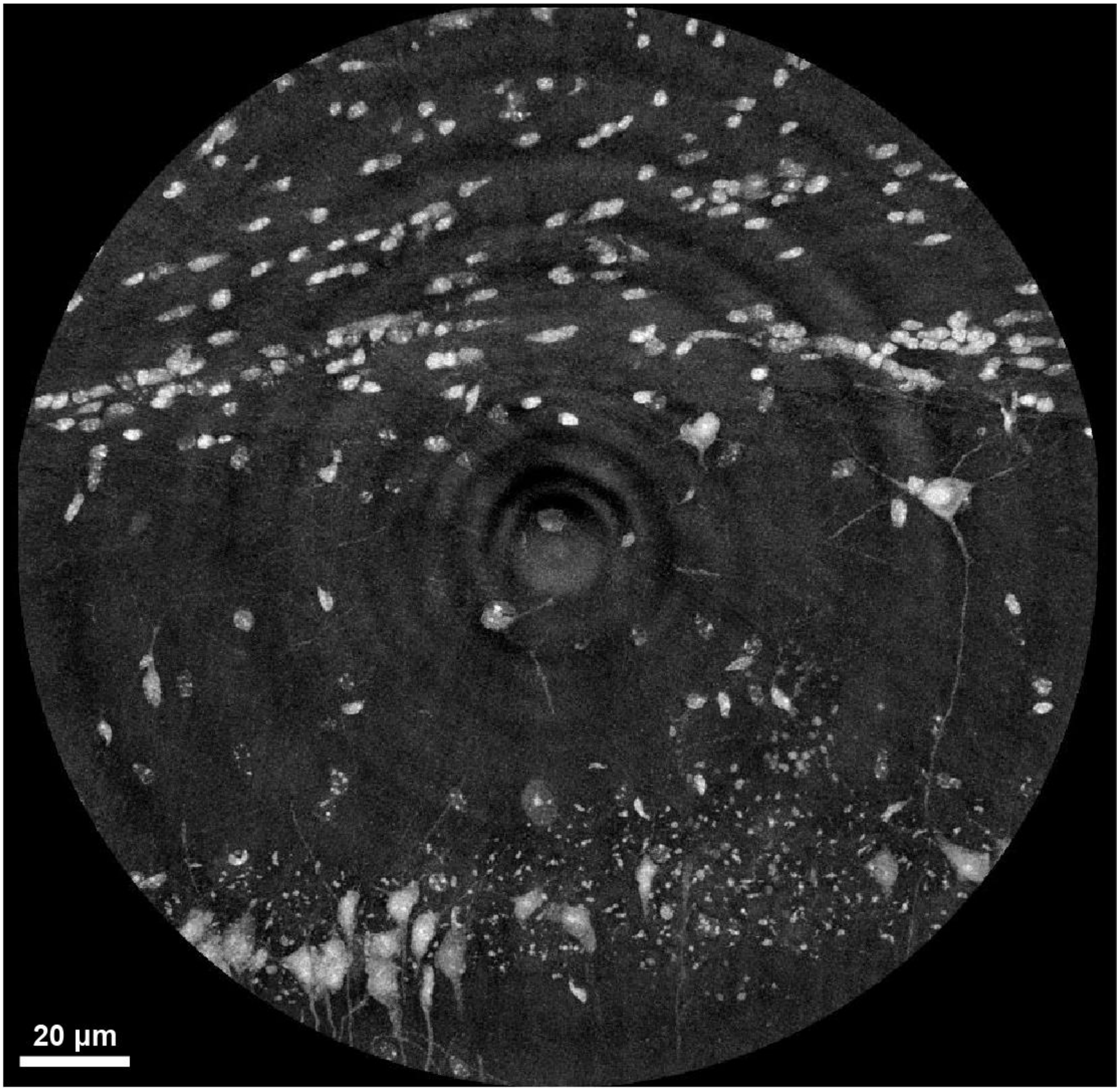

Suppl. Video 8 | Stack of consecutive 0.1^3 μm^3 voxel X-PCI-CT hippocampal MIPs, computed with the dataset in Suppl. Video 7.

ADDITIONAL REFERENCES:

1. Terman, A. & Brunk, U. T. Lipofuscin: mechanisms of formation and increase with age. *APMIS* **106**, 265–276 (1998).

2. Pinzer, B. R. *et al.* Imaging brain amyloid deposition using grating-based differential phase contrast tomography. *Neuroimage* **61**, 1336–1346 (2012).

3. Massimi, L. *et al.* Exploring Alzheimer's disease mouse brain through X-ray phase contrast tomography: From the cell to the organ. *Neuroimage* **184**, 490–495 (2019).

4. Noda-Saita, K. *et al.* Quantitative analysis of amyloid plaques in a mouse model of Alzheimer's disease by phase-contrast X-ray computed tomography. *Neuroscience* **138**, 1205–1213 (2006).

5. Töpperwien, M., van der Meer, F., Stadelmann, C. & Salditt, T. Correlative x-ray phase-contrast tomography and histology of human brain tissue affected by Alzheimer's disease. *Neuroimage* **210**, 116523 (2020).

6. Mittone, A. *et al.* Characterization of a sCMOS-based high-resolution imaging system. *J. Synchrotron Radiat.* **24**, 1226–1236 (2017).

7. Suortti, P. *et al.* Fixed-exit monochromator for computed tomography with synchrotron radiation at energies 18-90keV. *J. Synchrotron Radiat.* **7**, 340–347 (2000).

8. Mirone, A., Brun, E., Gouillart, E., Tafforeau, P. & Kieffer, J. The PyHST2 hybrid distributed code for high speed tomographic reconstruction with iterative reconstruction and a priori knowledge capabilities. *Nucl. Instruments Methods Phys. Res. Sect. B* **324**, 41–48 (2014).

9. Paganin, D., Mayo, S. C., Gureyev, T. E., Miller, P. R. & Wilkins, S. W. Simultaneous phase and amplitude extraction from a single defocused image of a homogeneous object. *J. Microsc.* **206**, 33–40 (2002).

10. Mader, K. *et al.* High-throughput full-automatic synchrotron-based tomographic microscopy. *J. Synchrotron Radiat.* **18**, 117–124 (2011).

11. Stampanoni, M. *et al.* TOMCAT: A beamline for TOmographic Microscopy and Coherent rAdiology experimenTs. *AIP Conf. Proc.* **879**, 848–851 (2007).

12. Khimchenko, A. *et al.* Hard X-Ray Nanoholotomography: Large-Scale, Label-Free, 3D Neuroimaging beyond Optical Limit. *Adv. Sci.* **5**, 1700694 (2018).

13. Mokso, R., Cloetens, P., Maire, E., Ludwig, W. & Buffiere, J.-Y. Nanoscale zoom tomography with hard x rays using Kirkpatrick-Baez optics. *Appl. Phys. Lett.* **90**, 144104 (2007).

14. Da Silva, J. C. *et al.* Efficient concentration of high-energy x-rays for diffraction-limited imaging resolution. *Optica* **4**, 492–495 (2017).

15. Pacureanu, A., da Silva, J. C., Yang, Y., Bohic, S. & Cloetens, P. Nanoscale three-dimensional imaging of biological tissue with x-ray holographic tomography. in *Proceedings of the SPIE* **10711**, 107112B (2018).

16. Cloetens, P., Barrett, R., Baruchel, J., Guigay, J.-P. & Schlenker, M. Phase objects in synchrotron radiation hard x-ray imaging. *J. Phys. D Appl. Phys.* **29**, 133–146 (1996).

17. Bleuet, P. *et al.* A hard x-ray nanoprobe for scanning and projection nanotomography. *Rev. Sci. Instrum.* **80**, 56101 (2009).

18. Bartels, M., Krenkel, M., Cloetens, P., Möbius, W. & Salditt, T. Myelinated mouse nerves studied by X-ray phase contrast zoom tomography. *J. Struct. Biol.* **192**, 561–568 (2015).

19. Cloetens, P. *et al.* Holotomography: Quantitative phase tomography with micrometer resolution using hard synchrotron radiation x rays. *Appl. Phys. Lett.* **75**, 2912–2914 (1999).

20. Zabler, S., Cloetens, P., Guigay, J.-P., Baruchel, J. & Schlenker, M. Optimization of phase contrast imaging using hard x rays. *Rev. Sci. Instrum.* **76**, 73705 (2005).

21. Lyckegaard, A., Johnson, G. & Tafforeau, P. Correction of ring artifacts in X-ray tomographic images. *Int. J. Tomogr. Stat.* **18**, 1–9 (2011).

22. Schneider, C. A., Rasband, W. S. & Eliceiri, K. W. NIH Image to ImageJ: 25 years of image analysis. *Nat.*

Methods **9**, 671–675 (2012).

23. Kapur, J. N., Sahoo, P. K. & Wong, A. K. C. A new method for gray-level picture thresholding using the entropy of the histogram. *Comput. Vision, Graph. Image Process.* **29**, 273–285 (1985).

24. Bolte, S. & Cordelières, F. P. A guided tour into subcellular colocalization analysis in light microscopy. *J. Microsc.* **224**, 213–232 (2006).

25. McLachlan, G. & Peel, D. *Finite Mixture Models*. (John Wiley & Sons, Inc., 2000). doi:10.1002/0471721182

26. Paxinos, G. & Franklin, K. B. J. *Paxinos and Franklin's The mouse brain in stereotaxic coordinates*. (Academic Press, 2001).

27. Sanchez-Cano, C. *et al.* Synchrotron X-Ray Fluorescence Nanoprobe Reveals Target Sites for Organo-Osmium Complex in Human Ovarian Cancer Cells. *Chemistry* **23**, 2512–2516 (2017).

28. De Samber, B. *et al.* Nanoscopic X-ray fluorescence imaging and quantification of intracellular key-elements in cryofrozen Friedreich's ataxia fibroblasts. *PLoS One* **13**, e0190495 (2018).

29. Ortega, R., Cloetens, P., Devès, G., Carmona, A. & Bohic, S. Iron storage within dopamine neurovesicles revealed by chemical nano-imaging. *PLoS One* **2**, e925–e925 (2007).

30. Que, E. L. *et al.* Quantitative mapping of zinc fluxes in the mammalian egg reveals the origin of fertilization-induced zinc sparks. *Nat. Chem.* **7**, 130 (2014).

31. Solé, V. A., Papillon, E., Cotte, M., Walter, P. & Susini, J. A multiplatform code for the analysis of energy-dispersive X-ray fluorescence spectra. *Spectrochim. Acta Part B At. Spectrosc.* **62**, 63–68 (2007).

32. Ullmann, J. F. P. *et al.* Segmentation of the C57BL/6J mouse cerebellum in magnetic resonance images. *Neuroimage* **62**, 1408–1414 (2012).

33. Hussain, R. Z. *et al.* Laquinimod has no effects on brain volume or cellular CNS composition in the F1 3xTg-AD/C3H mouse model of Alzheimer's disease. *J. Neuroimmunol.* **309**, 100–110 (2017).

34. Iulita, M. F. *et al.* Intracellular Aβ pathology and early cognitive impairments in a transgenic rat

overexpressing human amyloid precursor protein: a multidimensional study. *Acta Neuropathol. Commun.* **2**, 61 (2014).

35. Rosenberg, R. N., Fu, M. & Lambracht-Washington, D. Active full-length DNA Aβ(42) immunization in 3xTg-AD mice reduces not only amyloid deposition but also tau pathology. *Alzheimers. Res. Ther.* **10**, 115 (2018).

36. Castillo-Carranza, D. L. *et al.* Tau Immunotherapy Modulates Both Pathological Tau and Upstream Amyloid Pathology in an Alzheimer's Disease Mouse Model. *J. Neurosci.* **35**, 4857 LP – 4868 (2015).

37. Boche, D. *et al.* Reduction of aggregated Tau in neuronal processes but not in the cell bodies after Aβ42 immunisation in Alzheimer's disease. *Acta Neuropathol.* **120**, 13–20 (2010).

38. Wróbel, P. M. *et al.* Feasibility study of elemental analysis of large population of formalin fixed paraffin embedded tissue samples – preliminary results. *Spectrochim. Acta Part B At. Spectrosc.* **173**, 105971 (2020).

39. Morris, C. M., Candy, J. M., Oakley, A. E., Bloxham, C. A. & Edwardson, J. A. Histochemical distribution of non-haem iron in the human brain. *Acta Anat. (Basel).* **144**, 235–257 (1992).

40. Wei, H. *et al.* Imaging whole-brain cytoarchitecture of mouse with MRI-based quantitative susceptibility mapping. *Neuroimage* **137**, 107–115 (2016).

41. Meadowcroft, M. D., Connor, J. R., Smith, M. B. & Yang, Q. X. MRI and histological analysis of beta-amyloid plaques in both human Alzheimer's disease and APP/PS1 transgenic mice. *J. Magn. Reson. Imaging* **29**, 997–1007 (2009).

42. Vanhoutte, G., Dewachter, I., Borghgraef, P., Van Leuven, F. & Van der Linden, A. Noninvasive in vivo MRI detection of neuritic plaques associated with iron in APP[V717I] transgenic mice, a model for Alzheimer's disease. *Magn. Reson. Med.* **53**, 607–613 (2005).

43. Delatour, B., Epelbaum, S., Petiet, A. & Dhenain, M. In vivo imaging biomarkers in mouse models of Alzheimer's disease: are we lost in translation or breaking through? *Int. J. Alzheimers. Dis.* **2010**, 604853 (2010).

44. Tafoya, M. A., Madi, S. & Sillerud, L. O. Superparamagnetic nanoparticle-enhanced MRI of Alzheimer's disease plaques and activated microglia in 3X transgenic mouse brains: Contrast optimization. *J. Magn. Reson. Imaging* **46**, 574–588 (2017).

45. Atrophy, N. Seven-Tesla MRI and neuroimaging biomarkers for Alzheimer's disease. **39**, (2015).

46. Barbone, G. E. *et al.* Micro-imaging of Brain Cancer Radiation Therapy Using Phase-contrast Computed Tomography. *Int. J. Radiat. Oncol. Biol. Phys.* **101**, 965–984 (2018).

47. Kroemer, G. *et al.* Classification of cell death: recommendations of the Nomenclature Committee on Cell Death 2009. *Cell Death Differ.* **16**, 3–11 (2009).

48. Gallyas, E., Zoltay, G. & Dames, W. Formation of 'dark' (argyrophilic) neurons of various origin proceeds with a common mechanism of biophysical nature (a novel hypothesis). *Acta Neuropathol.* **83**, 504–509 (1992).

49. Switzer, R. C. Application of Silver Degeneration Stains for Neurotoxicity Testing. *Toxicol. Pathol.* **28**, 70–83 (2000).

50. Schmued, L. C. & Hopkins, K. J. Fluoro-Jade B: a high affinity fluorescent marker for the localization of neuronal degeneration. *Brain Res.* **874**, 123–130 (2000).

51. Schmued, L. C., Stowers, C. C., Scallet, A. C. & Xu, L. Fluoro-Jade C results in ultra high resolution and contrast labeling of degenerating neurons. *Brain Res.* **1035**, 24–31 (2005).

52. Kovalenko, T. N., Ushakova, G. A., Osadchenko, I., Skibo, G. G. & Pierzynowski, S. G. THE NEUROPROTECTIVE EFFECT OF 2-OXOGLUTARATE IN THE EXPERIMENTAL ISCHEMIA OF HIPPOCAMPUS. *J. Physiol. Pharmacol.* **62**, 239–246 (2011).

53. Sivaraman, D., Panneerselvam, P. & Muralidharan, P. Revealing hallmark histology of hippocampus neurons in beta-amyloid induced alzheimer ' s mice and investigation of neuroprotective effect of Ipomoea aquatic forsk , an Indian medicinal herb. *J. Chem. Pharm. Res.* **7**, 424–434 (2015).

54. Alexandru, A. *et al.* Selective Hippocampal Neurodegeneration in Transgenic Mice Expressing Small Amounts of Truncated Abeta Is Induced by Pyroglutamate – Abeta Formation. *J. Neurosci.* **31**, 12790–12801 (2011).

55. Beattie, B. L. & Mackenzie, I. R. A. Dementia diagnosis: Case presentations and neuropathology. *BC Med. J.* **47**, 480–486 (2005).

56. Oddo, S., Billings, L., Kesslak, J. P., Cribbs, D. H. & LaFerla, F. M. Abeta Immunotherapy Leads to Clearance of Early, but Not Late, Hyperphosphorylated Tau Aggregates via the Proteasome. *Neuron* **43**, 321–332 (2004).

57. Honig, L. S. *et al.* Trial of Solanezumab for Mild Dementia Due to Alzheimer's Disease. *N. Engl. J. Med.* **378**, 321–330 (2018).

58. Oddo, S. *et al.* Triple-Transgenic Model of Alzheimer ' s Disease with Plaques and Tangles : Intracellular Aβ and Synaptic Dysfunction. *Neuron* **39**, 409–421 (2003).

59. Hetman, M. & Pietrzak, M. Emerging roles of the neuronal nucleolus. *Trends Neurosci.* **35**, 305–314 (2012).

60. Lyons, M. R. & West, A. E. Mechanisms of specificity in neuronal activity-regulated gene transcription. *Prog. Neurobiol.* **94**, 259–295 (2011).

61. Sukhorukova, E. G., Grigoriev, I. P., Kirik, O. V, Alekseeva, O. S. & Korzhevskii, D. E. Intranuclear localization of iron in neurons of mammalian brain. *J. Evol. Biochem. Physiol.* **49**, 370–372 (2013).

62. Kurz, T., Eaton, J. W. & Brunk, U. T. The role of lysosomes in iron metabolism and recycling. *Int. J. Biochem. Cell Biol.* **43**, 1686–1697 (2011).

63. Everett, J., Brooks, J., Collingwood, J. F. & Telling, N. D. Nanoscale chemical speciation of β-amyloid/iron aggregates using soft X-ray spectromicroscopy. *Inorg. Chem. Front.* **8**, 1439–1448 (2021).

64. Everett, J. *et al.* Biogenic metallic elements in the human brain? *Sci. Adv.* **7**, eabf6707 (2021).

65. Cebrián-Silla, A. *et al.* Unique Organization of the Nuclear Envelope in the [3natal] Quiescent Neural Stem

Cells. *Stem cell reports* **9**, 20^{3}–216 (2017).

66. Cornelison, G. L., Levy, S. A., Jenson, T. & Frost, B. Tau-induced nuclear envelope invagination causes a toxic accumulation of mRNA in Drosophila. *Aging Cell* **18**, e12847 (2019).

67. Paonessa, F. *et al.* Microtubules Deform the Nuclear Membrane and Disrupt Nucleocytoplasmic Transport in Tau-Mediated Frontotemporal Dementia. *Cell Rep.* **26**, 582-593.e5 (2019).

68. Frost, B., Bardai, F. H. & Feany, M. B. Lamin Dysfunction Mediates Neurodegeneration in Tauopathies. *Curr. Biol.* **26**, 129–136 (2016).

69. Frost, B. Alzheimer's disease: An acquired neurodegenerative laminopathy. *Nucleus* **7**, 275–283 (2016).

70. Bruno, V. *et al.* The Neuroprotective Activity of Group-II Metabotropic Glutamate Receptors Requires New Protein Synthesis and Involves a Glial–Neuronal Signaling. *J. Neurosci.* **17**, 1891 LP – 1897 (1997).

71. Bruno, V. *et al.* Neuroprotection by Glial Metabotropic Glutamate Receptors Is Mediated by Transforming Growth Factor-β. *J. Neurosci.* **18**, 9594 LP – 9600 (1998).

72. Corti, C. *et al.* The Use of Knock-Out Mice Unravels Distinct Roles for mGlu2 and mGlu3 Metabotropic Glutamate Receptors in Mechanisms of Neurodegeneration/Neuroprotection. *J. Neurosci.* **27**, 8297 LP – 8308 (2007).

73. Battaglia, G. *et al.* Activation of mGlu3 receptors stimulates the production of GDNF in striatal neurons. *PLoS One* **4**, e6591–e6591 (2009).

74. Battaglia, G. *et al.* Activation of mGlu3 metabotropic glutamate receptors enhances GDNF and GLT-1 formation in the spinal cord and rescues motor neurons in the SOD-1 mouse model of amyotrophic lateral sclerosis. *Neurobiol. Dis.* **74**, 126–136 (2015).

75. Di Liberto, V., Bonomo, A., Frinchi, M., Belluardo, N. & Mudò, G. Group II metabotropic glutamate receptor activation by agonist LY379268 treatment increases the expression of brain derived neurotrophic factor in the mouse brain. *Neuroscience* **165**, 863–873 (2010).

76. Caraci, F. *et al.* Targeting Group II Metabotropic Glutamate (mGlu) Receptors for the Treatment of Psychosis Associated with Alzheimer's Disease: Selective Activation of mGlu2 Receptors Amplifies β-Amyloid Toxicity in Cultured Neurons, Whereas Dual Activation of mGlu2 . *Mol. Pharmacol.* **79**, 618 LP – 626 (2011).

77. Durand, D. *et al.* Astroglial mGlu3 receptors promote alpha-secretase-mediated amyloid precursor protein cleavage. *Neuropharmacology* **79**, 180–189 (2014).

78. Motolese, M. *et al.* Targeting type-2 metabotropic glutamate receptors to protect vulnerable hippocampal neurons against ischemic damage. *Mol. Brain* **8**, 66 (2015).

79. Taylor, D. L., Diemel, L. T., Cuzner, M. L. & Pocock, J. M. Activation of group II metabotropic glutamate receptors underlies microglial reactivity and neurotoxicity following stimulation with chromogranin A, a peptide up-regulated in Alzheimer’s disease. *J. Neurochem.* **82**, 1179–1191 (2002).

80. Taylor, D. L., Jones, F., Kubota, E. S. F. C. S. & Pocock, J. M. Stimulation of Microglial Metabotropic Glutamate Receptor mGlu2 Triggers Tumor Necrosis Factor α-Induced Neurotoxicity in Concert with Microglial-Derived Fas Ligand. *J. Neurosci.* **25**, 2952 LP – 2964 (2005).

81. Singleton, M. K. *et al.* MeCP2 is required for global heterochromatic and nucleolar changes during activity-dependent neuronal maturation. *Neurobiol. Dis.* **43**, 190–200 (2011).

82. Davis, B. M., Salinas-Navarro, M., Cordeiro, M. F., Moons, L. & De Groef, L. Characterizing microglia activation: a spatial statistics approach to maximize information extraction. *Sci. Rep.* **7**, 1576 (2017).

83. Landfield, P. W., Braun, L. D., Pitler, T. A., Lindsey, J. D. & Lynch, G. Hippocampal aging in rats: A morphometric study of multiple variables in semithin sections. *Neurobiol. Aging* **2**, 265–275 (1981).

84. Benavides-Piccione, R., Hamzei-Sichani, F., Ballesteros-Yáñez, I., DeFelipe, J. & Yuste, R. Dendritic Size of Pyramidal Neurons Differs among Mouse Cortical Regions. *Cereb. Cortex* **16**, 990–1001 (2005).

85. Komulainen, E. *et al.* JNK1 controls dendritic field size in L2/3 and L5 of the motor cortex, constrains soma size, and influences fine motor coordination. *Front. Cell. Neurosci.* **8**, 272 (2014).

86. Flood, D. G. & Coleman, P. D. Neuron numbers and sizes in aging brain: Comparisons of human, monkey, and rodent data. *Neurobiol. Aging* **9**, 453–463 (1988).
